# Carotenoid-based immune response in sea cucumbers relies on newly identified coelomocytes—the carotenocytes

**DOI:** 10.3389/fimmu.2025.1668167

**Published:** 2025-11-06

**Authors:** Noé Wambreuse, Estelle Bossiroy, Frank David, Céline Vanwinge, Laurence Fievez, Fabrice Bureau, Sylvain Gabriele, Tania Karasiewicz, Cyril Mascolo, Ruddy Wattiez, Igor Eeckhaut, Guillaume Caulier, Jérôme Delroisse

**Affiliations:** 1 Biology of Marine Organisms and Biomimetics Unit, Research Institute for Biosciences, University of Mons, Mons, Belgium; 2 Direction Générale Déléguée à la Recherche, l’Expertise, la Valorisation et l’Enseignement (DGD REVE), Muséum National d’Histoire Naturelle (MNHN), Station Marine de Concarneau, Concarneau, France; 3 Flow Cytometry Platform, GIGA Research Institute, University of Liège, Liège, Belgium; 4 Laboratory of Cellular and Molecular Immunology, GIGA Research Institute, University of Liège, Liège, Belgium; 5 SYMBIOSE Lab, Research Institute for Biosciences, CIRMAP, University of Mons, Mons, Belgium; 6 Proteomics and Microbiology Unit, Research Institute for Biosciences, University of Mons, Mons, Belgium; 7 Belaza Marine Station (IH.SM-UMONS-ULIEGE), Toliara, Madagascar

**Keywords:** immune cell, antioxidant, Echinodermata, haemocyte, deuterostome, reactive oxygen species, hydrovascular system, gene expression

## Abstract

Sea cucumbers are marine deuterostomes possessing a complex innate immune system composed of a wide diversity of immune cells—coelomocytes—making them compelling models for exploring the evolution of immunity. This study investigates the functional specialisation of coelomocytes within the two main echinoderm body fluids, namely, the perivisceral fluid (PF) from the perivisceral cavity and the hydrovascular fluid (HF) from the hydrovascular–ambulacral system. Given their distribution restricted to the HF, haemocyte-like cells (HELs) are particularly investigated. In echinoderms, haemocytes have been described as reddish cells containing haemoglobin and thus presenting a function in oxygen transport. Using an integrative approach that combines cell morphological analyses, pigment profiling, and multi-omics technologies, we demonstrate in the sea cucumber *Holothuria forskali* that HELs harbour exceptionally high concentrations of carotenoids, primarily canthaxanthin and astaxanthin, potent antioxidant molecules responsible for their pigmentation. Transcriptomics and proteomics analyses reveal that HELs express candidate genes involved in the carotenoid metabolism pathway as well as catalase, an antioxidant enzyme. Additionally, spectral flow cytometry assays reveal that HELs do not produce reactive oxygen species (ROS) in contrast to most coelomocyte types, reinforcing the hypothesis of their antioxidant function. HELs also contribute to the formation of large red bodies (i.e., coelomocyte aggregates) and increase in concentration following lipopolysaccharide injections, indicating an active role in immunity. Given these results, we hypothesise that these cells act after the culmination of the immune response, forming an antioxidant shell around the cellular aggregates to mitigate oxidative stress from ROS produced while encapsulating pathogens, thus protecting the host tissues. The discovery of carotenoid-carrying coelomocytes constitutes the first report of pigmented coelomocytes in sea cucumbers (except respiratory pigments), challenging the long-standing assumption that these cells contain haemoglobin. Therefore, we propose renaming haemocytes into carotenocytes, at least in this species. However, we believe that this newly described coelomocyte type has been misidentified as haemoglobin-containing cells in many previous studies and may be present in many other holothuroid species. Our findings thus establish a new paradigm in the study of coelomocytes in echinoderms, as well as in the function of the hydrovascular system, which is unique to this phylum.

## 1 Introduction

Echinoderms represent a phylum of marine deuterostomes that share many molecular features with chordates (1–3). These metazoans display a complex innate immune system that primarily relies on coelomocytes—specialised circulating immune cells suspended in the fluids that fill the coelomic cavities (4–7). These include the hydrovascular and perivisceral fluids, which could, roughly speaking, be considered functional analogues of blood (6; 8). The hydrovascular fluid (HF) fills the hydrovascular system, a system unique to echinoderms, which performs a wide range of functions, including locomotion, transport of metabolites, and support of body structure (9–12). The perivisceral fluid (PF) is the fluid surrounding the organs in the general cavity and is known to play critical functions in the transport of metabolites and humoral factors as well as water balance regulation (8, 13). Over the past decades, coelomocytes have been shown to play a large range of immune functions, including the recognition and elimination of foreign materials and pathogens, phagocytosis, aggregation, encapsulation, and the production of a wide variety of humoral factors (7, 14, 15). In addition, the sequencing of the genome of the sea urchin *Strongylocentrotus purpuratus* has revealed a wide variety of genes encoding pathogen recognition receptors, making these organisms very interesting models for investigating the evolution of innate immunity (2). In this context, numerous studies have examined the transcriptomics response of coelomocytes to various stressors (16–19), highlighting, among other things, the expression of homologues of complement system components (1, 20), known to interact with the adaptive immune systems of vertebrates and to contribute to opsonisation. This example demonstrates that the study of the immune system of echinoderms offers cutting-edge information on the evolution of the immune system in deuterostomes. Nevertheless, while PF coelomocytes have been the subject of in-depth studies (e.g., 16, 21), HF coelomocytes have received comparatively little attention. This could be partly due to the technical difficulty of collecting sufficient HF, especially in sea urchins or brittle stars, in which the rigid endoskeleton limits access to the hydrovascular appendages containing a low volume of fluid. Sea cucumbers, on the other hand, with their prominent HF appendages, including the large Polian vesicle(s) (e.g., 22, 23), and their soft bodies, represent an appropriate model for studying the hydrovascular system in general and, in particular, the function of hydrovascular coelomocytes.

Sea cucumbers are also of considerable interest for multiple reasons. First, many species play a pivotal role in marine ecosystems by representing important components of benthic macrofauna and by participating in sediment bioturbation (24, 25). Secondly, some species possess a high commercial value due to their exploitation in traditional Chinese pharmacopoeia and gastronomy (26). To this purpose, some species are farmed under aquaculture conditions with complete life cycle control (27, 28). The model species of this study, the European sea cucumber *Holothuria forskali*, has been considered in particular in the context of integrated multi-trophic aquaculture (IMTA) (29, 30). Thirdly, sea cucumbers are a source of bioactive compounds extensively explored for the development of drugs with antibiotic and anticancer properties, among others (31–34). For instance, the farmed Chinese sea cucumber *Apostichopus japonicus* is rich in astaxanthin, a powerful antioxidant used in many pharmaceutical products (35). These various characteristics highlight that sea cucumbers warrant research interest from both a fundamental and applied point of view.

Like vertebrates, sea cucumbers have different immune cell types and appear to have the highest diversity among the five echinoderm classes (7), with between six and nine types depending on the classification (e.g., six according to Chia and Xing (36) and Smith et al. (7); eight in Hetzel (5); and nine in Queiroz and Custódio (37)). The most accepted cell types are phagocytes, spherule cells, progenitor cells, fusiform cells, crystal cells, and haemocytes. Nevertheless, it should be noted that the presence of these cells is highly species-dependent and that many different names have also been used to refer to them in the literature (reviewed by Queiroz and Custódio (37)), which has caused much confusion. Coelomocyte classifications are mainly based on morphological criteria; however, functional information on the different cell types is scarce. For example, only one study provides transcriptomics data on coelomocyte subsets in sea cucumbers (38), and these subsets themselves constitute a mixture of “spherical cells” and “lymphoid-like cells” (i.e., not directly related to any previous morphological classification). Among the coelomocyte types, the function and distribution of haemocytes, a type of coloured cell thought to contain haemoglobin (6), remain particularly enigmatic (22). While previous reports claim that they have a function in oxygen transport and are limited to the holothuroid orders Molpadida and Dendrochirotida (5, 6, 39, 40), recent research has shown that they have a wider distribution than previously described, also occurring in the order Holothuriida (formerly within a larger taxon, the Aspidochirotida) (22). Furthermore, it has been shown that these cells can participate in the encapsulation process, and it is suggested that their haemoglobin would release reactive oxygen species (ROS) during the immune response (22, 23, 41). These studies have also shown that the HF of several species, including *H. forskali*, is particularly rich in this cell type, thus offering a promising opportunity to deepen our knowledge about these pigmented coelomocytes.

The present study, therefore, seeks to investigate the differences in the immune response of circulating coelomocytes between HF and PF, the two main body fluids of sea cucumbers, using an integrative approach combining morphological analyses, pigment profiling, and multi-omics technologies. With their particular distribution, mainly localised in the HF, the function of cells similar to haemocytes (i.e., colourful reddish coelomocytes) is specifically studied. These cells are referred to as haemocyte-like cells (HELs) in this study to avoid any confusion and prior functional assumptions related to the presence of haemoglobin. Overall, this study provides new insights into the immune response of sea cucumbers and establishes new paradigms on the function of HELs, which we describe here as a new functional coelomocyte type.

## 2 Materials and method

### 2.1 Coelomocyte collection and morphological characterisation

#### 2.1.1 Collection and maintenance of organisms

Adult specimens of *Holothuria forskali* Delle Chiaje, 1824 (42 individuals in total), were obtained by the collection service of the Roscoff Biological Station, from where they were initially collected by scuba diving in Morlaix Bay (Brittany, France) just before their shipment. As soon as they were delivered to the University of Mons (Belgium), the specimens were kept in a closed-circuit tank containing 400 L of filtered seawater, with a substrate made of small pebbles, at a temperature varying between 14 °C and 17 °C throughout the year and salinity between 33 and 35 psu. An artificial circadian rhythm was recreated using neon lighting set at a constant exposure time from 8 a.m. and 8 p.m. The specimens were fed once a week with a mix of dried algae in agar-agar-based gel. Before any experiments, the specimens were acclimatised for at least 2 weeks in the tanks.

#### 2.1.2 Coelomocyte harvesting from the two body fluids

For each specimen, coelomocytes were collected from the two body fluids of interest: PF from the general cavity and HF from the Polian vesicle as per Wambreuse et al. (18). Briefly, a longitudinal incision was first made on the *bivium*, from the anterior to the posterior part of the animal, using a scalpel to open the integument between two radial canals (to avoid any contamination with HF), allowing PF to be harvested in a 15-ml tube. The incision was then extended anteriorly to access the Polian vesicle, which was then placed over another 15-ml tube to pierce it and harvest the dropping HF. Body fluids were systematically placed on ice before subsequent analyses to avoid coelomocyte aggregation.

#### 2.1.3 Establishment of cell concentration and proportion

Typically, 20 µl of each body fluid was pipetted directly in the collected HF and PF and mixed at an equivalent volume with calcium- and magnesium-free artificial seawater containing EDTA (CMFSW + EDTA: 460 mM NaCl; 10.7 mM KCl; 7 mM Na_2_SO_4_; 2.4 mM NaHCO_3_; 20 mM HEPES; 70 mM EDTA; pH = 7.4) to avoid cell aggregation (21). Cells were then counted using a Neubauer hemacytometer within the hour post-body fluid collection. To do this, 10 µl was placed on the hemacytometer, and the 16 squares corresponding to a total volume of 0.1 mm³ were photographed under a microscope (Axio Imager A1, Zeiss). Cells were counted manually using ImageJ software V1.54f, and their concentrations per millilitre were calculated using the following formula:


Concentration (cells/ml) = n × dilution factor × 10,000

Once concentrations were obtained, the proportion could also be calculated following the formula:


$$\begin{array}{c} Proportion\;\left(\%\right)\;=\;(concentration\;of\;the\;cell\;population\;of\;interest/\\ concentration\;of\;all\;coelomocyte\;populations)\times 100 \end{array}$$

Note that if spermatozoa were present, which is common during coelomic fluid harvesting in holothuroids (see Caulier et al. (22)), they were not assimilated into a coelomocyte population (i.e., not included in the coelomocyte counts). Cell populations were identified based on previous studies on holothuroid coelomocytes (36, 37). To highlight significant differences in the concentration and proportion of cell populations between the two body fluids, a Wilcoxon signed-rank test was performed in R V.4.4.2 (α = 0.05).

#### 2.1.4 Microscopic analysis of coelomocytes and macroscopic pictures

Depending on the needs, cells were observed using different microscopy techniques, including light microscopy, fluorescence microscopy, and scanning electron microscopy (SEM). Light microscopy and fluorescence microscopy were performed on fresh samples using an epifluorescence microscope (Axio Imager A1, Zeiss) with or without an activated laser, depending on the type of microscopy desired. Three fluorescent filters could be used, yielding three different excitation wavelengths: 350 nm (corresponding to violet), 495 nm (corresponding to cyan), and 589 nm (corresponding to yellow). For the cell type description, the cell sizes were measured in the ImageJ software V1.54f, and the mean ± standard deviations (SD) were calculated for each cell type (n = 5–10). The SEM protocol was taken from Wambreuse et al. (18), which was initially adapted from Smith et al. (21). Briefly, this protocol includes an incubation phase during which cells can settle on the slide in a humid chamber for 30 min before being fixed with a glutaraldehyde solution. They are then dehydrated with successive baths of ethanol and chemically dried with successive baths of hexamethyldisilazane and coated with a thin layer of a mixture of palladium and gold (60:40%). Samples were then observed under a scanning electron microscope (JSM-7200F, JEOL). For the tissue samples, the same protocol was used, but the tissues were immersed directly in the fixation solution containing glutaraldehyde. Finally, some macroscopic pictures were taken to show certain parts of interest in the anatomy of the organisms; these were produced using a Leica M28 binocular camera or an Olympus TG-6 digital camera.

### 2.2 Monitoring of coelomocyte activity by time-lapse imaging

PF and HF were collected as described above (section 2.1.2) and placed directly on ice. 25 min later, 100 µl of the fluid to be analysed was deposited on a slide already mounted on the microscope and without a cover glass. After 5 min on the glass for the cells to settle, time-lapse images were captured at 30-s intervals for 30 min at 200**×** magnification (Axio Imager A1 microscope, Zeiss). The image sequences obtained were then loaded into ImageJ software V1.54f to build the videos. To quantify cell aggregation, the pixel threshold was automatically modified to obtain a binary value for each pixel. After adjusting the resolution to the actual pixel size, a “particle analysis” was carried out—the first targeting particles with a surface area between 4 and 50 µm^2^, and the second targeting particles with a surface area greater than 4 µm^2^. The output of these analyses includes the number of particles on each image and the mean particle area. These two analyses were used to count isolated HELs and follow the mean particle size (including cells and aggregates) over time, respectively. Finally, a linear least squares regression was applied to these time series, and a Mann–Kendall test was run in R V4.4.2 to check for a relation over time (α = 0.05).

### 2.3 Immunostimulation using lipopolysaccharide injection

In order to study the response of coelomocytes from both fluids to immunological stress, lipopolysaccharide (LPS) injections were performed 24 h prior to harvesting the body fluids. These injections consisted of 200 µl of sterile CMFSW without EDTA, containing 5 mg/ml of LPS from *Escherichia coli* O111:B4 (L2630; Merck). As a control, injections of CMFSW without LPS were used to avoid injection stress bias. In total, seven individuals received an LPS injection, and six received a control injection. These injections were carried out using 1-ml syringes with 23-g needles in the right anterior part of the animal. Immediately afterwards, the inoculated individuals were isolated in their tanks until the following day. Coelomocytes from both body fluids were collected, as previously explained (see section 2.1.2). Importantly, during the dissections, the sex of the individuals was systematically noted to avoid misinterpretation of results due to an unequal sex distribution between the conditions (identified based on the gonadal aspect according to Tuwo and Conand (42)). For each fluid sample, no more than 2 ml of fluid was used for the RNA extraction, and 20 µl was systematically retained for cell counting. The concentration and proportion of each coelomocyte population were calculated as above (see section 2.1.3). It should be noted that although contaminating spermatozoa were not included in the cell count after LPS challenge, they were counted for gene expression analysis to estimate the percentage of contamination. The samples selected for omics analyses were those with the lowest proportion of contamination. To reveal significant differences between control-injected and LPS-injected individuals in terms of concentration and proportion, a Mann–Whitney U test was performed in R V4.4.2 (α = 0.05).

### 2.4 Transcriptomics analysis using RNA-sequencing

#### 2.4.1 RNA extraction, library preparation, and sequencing

For RNA extraction, freshly collected body fluids were centrifuged at 500 × g and 4 °C for 5 min to pellet cells. The volume used for RNA extractions was noted and, combined with cell counts, allowed us to know exactly how many cells were in the pellet and the proportions of the different cell populations. RNA extractions were carried out using the Qiagen RNeasy mini kit according to the manufacturer’s instructions. Other samples were also prepared for RNA-sequencing (RNA-seq) to be used in other projects; these consisted of a stone canal and podia collected from other individuals, and their RNA extraction was performed using a TRI reagent kit according to the manufacturer’s instructions (Merck; T9424). The concentration and purity of extracted RNA were determined using a NanoDrop spectrophotometer (DeNovix DS11), and the RNA integrity value (RIN) was assessed using the Agilent 2100 Bioanalyzer (Agilent RNA 6000 Nano Kit). Only the three samples per condition showing the best RNA quality of HF and PF were retained for RNA-seq (note that HF and PF extracts were selected from the same individuals to allow intraindividual comparisons). Preparation of the cDNA libraries and sequencing were carried out by the Beijing Genomics Institute (BGI, Hong Kong). Briefly, the cDNA libraries were assembled as follows: mRNAs were isolated from total RNA using the oligo(dT) method; purified mRNAs were fragmented and reverse transcribed into the first cDNA strand, prior to synthesis of the second cDNA strand; double-stranded cDNA fragments were end-repaired, 3'-adenylated, and ligated to sequencing adapters; cDNA fragments of appropriate size were selected and enriched by PCR; PCR products were heat-denatured; and single-stranded DNA was cyclised by oligo splint and DNA ligase. The libraries were then sequenced on the BGISEQ-500 platform.

#### 2.4.2 Raw data filtering, *de novo* assembly, and gene expression level

Before assembly, raw data were filtered to eliminate adapter-polluted reads, reads containing more than 5% unknown bases, and low-quality reads (i.e., reads comprising more than 40% of bases with a quality value below 20). As no reference genome exists for *H. forskali*, the transcriptome was assembled *de novo* using Trinity software (V2.5.1). The obtained transcripts were then grouped using Tgicl software (V2.5.1) to eliminate redundancies and obtain the final sequences referred to as unigenes. The unigenes can either form clusters comprising several unigenes with more than 70% overlapping or singletons (i.e., single unigenes). To obtain the expression level of each unigene (or cluster), reads were mapped onto the transcriptome using Bowtie2 (V.2.2.5) and the unigene expression level was calculated using RSEM (V.1.2.12). The result is expressed as “fragments per kilobase of the transcript, per million mapped reads” (FPKM). As the sequence length is a proxy of the assembly quality, the size distribution of unigenes was represented.

#### 2.4.3 Functional annotation of unigenes

To assess the completeness of the assembled transcriptomes, BUSCO annotation was performed for each sequenced library and the merged transcriptome using the tool BUSCO in the Galaxy server (https://usegalaxy.eu; V5.4.6) against the metazoan_odb10 dataset (954 BUSCOs). The BUSCO metric attempts to provide a quantitative assessment of the completeness of genomics data by classifying orthologs into the following four categories: complete and single-copy, complete and duplicated, fragmented, or missing BUSCOs (43). Moreover, to obtain an initial indication of unigene function, the sequence of each unigene was aligned with several protein databases, including NCBI NT, NCBI NR, GO – Gene Ontology, KOG – EuKaryotic Orthologous Groups, KEGG – Kyoto Encyclopedia of Genes and Genomes, SwissProt and InterPro using Blast (V2.2.23), Diamond (V0.8.31), Blast2GO (V2.5.0), and InterProScan5 (V5.11-51.0). The unigene annotation provides an E-value that quantifies the degree of similarity with the annotation: only annotations with an E-value <10^−5^ were taken into account.

#### 2.4.4 Differential expression analysis

Differential expression analyses were performed to answer two main questions: 1) “What are the differentially expressed genes (DEGs) between control and LPS-injected individuals?” and 2) “What are the DEGs between HF and PF?” The first question was assessed for the PF (PF analysis; n = 3), HF (HF analysis; n = 3), and all fluids together (merged fluid analysis; n = 6). For this last analysis, we are aware that sampling is not completely independent, as samples from the same individuals are part of the same condition. Nevertheless, we have chosen to retain it as support for the results obtained on the two fluids separately. The second question was assessed for control individuals (CON analysis; n = 3), LPS-injected individuals (LPS analysis; n = 3), and all individuals together regardless of the stress condition (merged condition analysis; n = 6). In addition, differential expression analyses were performed between male and female samples to reveal a potential sex-specific expression in PF, HF (n = 2 in males and n = 4 in females), and merged fluids (n = 4 in males and n = 8 in females). Differential expression analyses were performed using the DESeq2 package. The output of these analyses consists mainly of two metrics, a fold change value (FC) and a false discovery rate (FDR; corresponding to a Wald statistical test p-value adjusted following the Benjamini–Hochberg procedure). Only unigenes having a |log_2_(FC) value| ≥1 and an FDR ≤0.05 were considered as significantly differentially expressed.

Heat maps were carried out using the package “Pheatmap” to illustrate the result based on normalised read count. Principal component analyses (PCA) were also generated to illustrate the heterogeneity in gene expression between different samples (based on log-transformed FPKM values and filtered by quantile-quantile to keep only the 25% most informative DEGs) in the MetaboAnalyst (V6.0) server (https://www.metaboanalyst.ca/). In addition, Venn diagrams were performed to highlight DEGs shared between the differential expression analyses. They were produced using the web tool “Venn” on the server https://bioinformatics.psb.ugent.be/webtools/Venn/.

#### 2.4.5 Functional enrichment analysis

Functional enrichment analyses were performed using KEGG annotation to reveal the most differentially expressed metabolic pathways. We chose to present, for each analysis, the 20 most enriched pathways and to group all these pathways in a table to compare the most enriched pathways between the different analyses (i.e., analyses comparing stress conditions and body fluids). For each pathway, various measures are provided: the pathway ranking (from 1 to 20), the q-value (Q) represented by a colour scale (the lower the q value, the more significant the differential expression of the pathway), the rich factor (RF) representing the percentage ratio between the number of annotated DEGs over the number of all annotated unigenes, the number of annotated DEGs in the pathways, and the ratio between up- and down-regulated unigenes within these DEGs.

### 2.5 Pigment identification and quantification by high-performance liquid chromatography and spectrophotometry

To obtain a first identification of the nature of the coelomocyte pigments, pigments were extracted using liquid–liquid phase extraction. This was conducted on lyophilised cell pellets from HF and PF of seven individuals of *H. forskali* to compare the distribution and amount of pigment between the two fluids. To estimate the number of cells in the pellet, the concentration of the fluid, as well as the volume, was determined prior to the centrifugation (as in section 2.1.3). In addition to holothuroid body fluids, two samples of bovine blood (*Bos taurus*), acquired from the conventional meat industry, were included in the analysis to serve as a positive control for the presence of haemoglobin, following previous hypotheses on haemocyte pigmentation (39, 44). Body fluids were centrifuged at 500 **×** g and 4 °C for 5 min to isolate the cells from the supernatant. Rapidly after the centrifugation, the different samples and positive controls were flash-frozen in liquid nitrogen before being freeze-dried under a vacuum at −60 °C (using a CHRIST Alpha 1–2 LDplus freeze-dryer) for 24 h. Dried samples were stored and protected from light at 6 °C until their processing. In order to determine the pigment affinity, several solvents were used to dissolve the lyophilised samples, including methanol, chloroform, and distilled water (volume ratio of 2:3:2.7) and separate pigments in different phases. First, 2 ml of ice-cold methanol (HPLC grade; ≥ 99.9%) was added, followed by 700 µl of ice-cold distilled water, before vortexing the samples for 1 min. Next, the cell membranes were disrupted using an ultrasonic homogeniser (U50 control, IKA Labortechnik) set to cycle 1 and 50% amplitude for 45 s. Once completed, 1.5 ml of ice-cold chloroform (HPLC grade; ≥ 99.9%) was added, and the samples were vortexed again for 1 min before being agitated on ice at 300 rpm for 10 min. After adding again 1.5 ml of ice-cold chloroform as well as 2 ml of ice-cold distilled water, the samples were centrifuged at 1,500 **×** g for 30 min at 4 °C. The resulting polar (water + methanol) and apolar (chloroform) phases were separated: 2 ml of each phase was collected in separate tubes and then centrifuged at 10,000 **×** g for 5 min to ensure that all debris were removed. The supernatants were then collected, and the absorption spectrum, from 220 to 750 nm of excitation wavelength, was obtained for each sample using a nanodrop spectrophotometer (DeNovix DS-11) with quartz cuvettes. Note that some samples of HF had to be diluted for the measurement to avoid the saturation of the device. After confirming that the red pigmentation could be assigned to carotenoids (see HPLC results; section 3.6), a quantification of the mass of carotenoids per million cells in the apolar phase was performed using a pure canthaxanthin standard (canthaxanthin_trans; Merck; 11775) of known concentration to obtain a calibration curve. This was done by measuring the absorbance of the standard at increasing dilutions in chloroform (maximum absorbance at 490 nm in chloroform; **Supplementary Figure 1A**). This allows us to quantify the mass of the carotenoid for each sample. As the dry masses of the HF and PF pellets were insufficient to be weighed, the abundance of pigments was normalised according to the number of cells in the pellet and expressed in µg of carotenoid per million cells. In order to highlight a putative significant difference between the two body fluids, a Wilcoxon signed-rank test was carried out in R V4.4.2 (α = 0.05). Moreover, knowing the number of HELs in each sample, a Pearson's correlation test was performed to see if the mass of carotenoids in each sample is correlated with the number of HELs (α = 0.05). This was done considering two body fluids (HF and PF; n = 14) and only considering HF samples (n = 7), and after checking the normality of data using the Shapiro–Wilk normality test in R V4.4.2 (α = 0.05). If the data normality criterion was not respected, Spearman’s correlation test was used instead (α = 0.05).

In order to obtain a better identification of carotenoid pigments, high-performance liquid chromatography (HPLC) was performed, using samples of HF, PF, and Polian vesicles (i.e., remaining Polian vesicle tissue after harvesting the HF, still containing large red aggregates; see section 3.7), which were collected from four individuals. Body fluid and Polian vesicle samples were prepared as explained in the spectrophotometry protocol. Once dried, the mass of Polian vesicles was measured using a high-precision balance (Sartorius Extend). For the extraction, samples were dissolved in a 1.5-ml extraction solution consisting of 95% methanol and 2% ammonium acetate with an additional 20 µg of trans-β-Apo-8'-carotenal (commercial standard, Roth^®^) that was added as an internal standard for mass quantification. Samples were then homogenised using beads at 30 Hz for 10 min with the Mixer Mill MM 400 (Retsch) and filtered with 0.2-µm mesh filters. Pigments were finally identified using an Agilent 1260 Infinity HPLC system following the methodology developed by David et al. (45), adapted from Brotas and Plante-Cuny (46). The relative abundance of pigments in each sample was determined by measuring the areas under the peaks (AUP) of the curve at a wavelength of 470 nm to target the carotenoid pigments (45). The AUPs of different pigments were standardised with the AUP of the known mass of the commercial standard (trans-β-Apo-8'-carotenal). The abundance of pigments in Polian vesicles was normalised according to their mass, whereas the abundance of pigments in the body fluid samples was converted into µg per million cells. To identify the chemical nature of the pigments, the resulting HPLC peaks were compared with peaks of commercial carotenoid compounds such as astaxanthin, canthaxanthin, and all-trans-echinenone (DHI Laboratory Products) that were also analysed by HPLC (**Supplementary Figure 1B**).

### 2.6 Analysis of coelomocyte autofluorescence using spectral flow cytometry

In order to quantify the autofluorescence of cells, samples of PF and HF were analysed by spectral flow cytometry. The two fluids were collected as previously described and directly mixed with an equivalent volume of CMFSW + EDTA to avoid clotting. Every step was carried out on ice to prevent cell deterioration. Samples were filtered with a cell strainer CellTrics equipped with a 100-µm mesh (Sysmex), and 10 µl was pipetted to establish the cell concentration. The samples were then centrifuged for 5 min and 4 °C at 500 × g to resuspend the pellet in the flow cytometry buffer (3× concentrated PBS buffer containing 20 mM HEPES and 50 mM EDTA; pH = 7.4; inspired by Smith et al. (21)) to obtain a final concentration of 10^7^ cells per ml. The Sony ID7000™ Spectral Cell Analyser, equipped with five lasers (355, 405, 488, 561, and 637 nm), was used for data acquisition. The gating strategy is shown in **Supplementary Figure 2**. 

### 2.7 Pigment analysis of HELs purified by fluorescent-activated cell sorting

HF and PF from one individual were sampled and prepared similarly as described for spectral flow cytometry (see section 2.6). The two fluids were then analysed using the BD FACSAria™ III cell sorter. The HEL population was easily identified by comparing the profile of the cells in the two body fluids. This population was sorted using fluorescence-activated cell sorting (FACS) on the basis of autofluorescence criteria, previously determined using the Sony ID7000™ Spectral Cell Analyser. Once sorted, the purity of the sample was calculated by analysing the sample again, using the same parameters. Their presence and purity were also assessed visually under the microscope (Axio Imager A1, Zeiss). Having established that the sample was sufficiently pure, cells were centrifuged at 500 × g and 4 °C for 5 min and the pellet was stored at −80 °C, protected from light, until the pigment analysis. For pigment extraction, the pellet was prepared as for spectrophotometry analysis (see section 2.5) and resuspended in 1 ml of chloroform before a brief sonication to release the pigment. After a brief centrifugation at 10,000 × g and 4 °C for 10 min to pellet cell debris, the sample was analysed by spectrophotometry (see section 2.5). To estimate the amount of carotenoid per cell, the absorbance obtained was compared with a commercial canthaxanthin standard (see section 2.5) using a calibration curve (see **Supplementary Figure 1A**). Knowing the initial number of HELs in the sample (number of events counted by the flow cytometer), the final quantity of carotenoid was calculated in µg per million HELs.

### 2.8 HEL gene and protein expression

#### 2.8.1 Relation between gene expression and HEL proportion

To test the relation between the proportion of HELs and the differential expression between PF and HF, a differential expression analysis was carried out between the HF and the PF for each individual. This analysis was conducted using the PoissonDis algorithm, which is based on the detection of DEGs on the FC and Poisson distribution according to Audic and Claverie (47). Only unigenes with a value of |log_2_(FC)| ≥1 and an FDR ≤0.05 were considered to be significantly differentially expressed. The proportion of DEGs obtained for each individual (i.e., the number of DEGs out of the total number of genes in the respective transcriptomes) was then correlated with the proportion of HELs in the HF, determined before RNA extraction by cell counting (see section 2.1.3). In addition to the DEGs, the fluid-specific expressed gene (FSEG) proportion was tested for a putative relation with the proportion of HELs in HF. These genes had a null expression in one of the two fluids and were identified based on Venn diagram analyses. To statistically test the relations, Pearson's correlation tests were performed (α = 0.05) after checking the normality of data using the Shapiro–Wilk normality test in R V.4.2.2 (α = 0.05).

#### 2.8.2 Proteomics analysis by mass spectrometry and validation of transcriptomics differential analysis

To validate the transcriptomics results and obtain the proteome of a highly enriched population of HELs, mass spectrometry (MS) was carried out on the PF and HF of one individual. Cells from the two body fluids were collected and counted as explained previously (see section 2.1.3). This individual in particular was selected because it had a very high proportion of HELs in its HF. The reason for such a high proportion of HELs in HF is unclear, but one possible explanation is that the individual used for this analysis was eviscerated, causing stress that may have triggered a specific response of HELs. After centrifugating for 5 min at 500 × g at 4 °C, cell pellets were isolated and flash-frozen. To perform the protein extraction, pellets were resuspended in a 5% acetic acid solution and 8 M urea and incubated for 1 h. Then, ultrasonication was performed three times for 10 s (amplitude 20**×**, cycle 1) before centrifuging the samples at 18,000 × g for 15 min to eliminate cell debris. The protein concentration in the supernatant was assessed using a Bradford assay with bovine gamma globulin as a standard, and concentrations were adjusted to 50 µg/µl for downstream processing. To reduce the proteins, a solution containing dithioerythritol (DTE) was added to obtain a final concentration of 12.5 mM before incubating for 25 min at 56 °C under agitation. The samples were then alkylated for 30 min in the dark by adding an iodoacetamide (IAA) solution at a final concentration of 25 mM. Proteins were precipitated by adding four volumes of ice-cold acetone, incubating for 4 h at −20 °C, and centrifuging at 4 °C for 20 min at 18,000 × g. After discarding the supernatant, the pellet was resuspended in a solution at 50 mM NH_4_HCO_3_ containing 2 µg/20 µl of trypsin (Promega) and incubated overnight at 37 °C in order to lyse proteins into peptides. The lysis was stopped by adding 5 µl of formic acid 0.5% to the 20 µl of solution. Samples were centrifuged a last time for 15 min at 11,000 × g and 4 °C. The peptide concentration was established using the Pierce™ Quantitative Colourimetric Peptide Assay kit, and the supernatant was mixed with the loading buffer containing 100 fmol/10 µl of PepCalMix to obtain a final peptide concentration of 4 µg/10 µl before the injection.

Protein identification and quantification were performed following a label-free strategy on a UHPLC HRMS platform (Eksigent 2D Ultra, AB SCIEX, TripleTOF™ 6600). Peptides (4 µg) were separated on a 15-cm C18 column (YMC-Triart 12 nm, S—3 µm, 150 × 0.3 mm ID, 1/32”) using a linear acetonitrile (ACN) gradient [3%-80% (v/v), in 75 min] in water containing 0.1% formic acid (v/v) at a flow rate of 5 µl min^−1^. Spectra were acquired in data-independent (DIA, SWATH) acquisition modes. For SWATH analysis, 100 incremental steps were defined as windows of variable m/z values over a 400/1,250-m/z mass range. The MS/MS working time for each window was 7 ms, leading to a duty cycle of 2.65 s per cycle. DIA analysis for identification and quantification was done using DIA-NN™ software (V1.9.2, for academic use) using the FASTA file format of the coelomocyte proteome of *H. forskali* and annotated with UniProt standards. This proteome was acquired based on the coding sequence prediction using the TransDecoder software (V3.0.1) from the merged coelomocyte transcriptome. Proteins with a peptide count <2 were filtered out due to their unreliable annotation.

To validate the transcriptomics differential expression analysis between HF and PF, the expression of proteins annotated with a DEG was compared with the expression of the corresponding DEGs. As one protein could be annotated by several genes, only proteins annotated with one gene were considered to ensure a reliable correspondence between the protein and the gene expression. An FC value of PF versus HF intensity was calculated for these proteins to correlate it with the FC value of transcriptomics analysis (under log_2_FC). It is important to note that no replication was used in calculating this FC value and that it is therefore not the result of a statistical test. Nevertheless, we chose to keep this single sample showing the highest concentration of carotenocytes in the HF, to support the transcriptomics analysis. To test this correlation, a Pearson's correlation test was performed (α = 0.05) after checking the normality of the data using a Shapiro–Wilk test on R V4.2.2 (α = 0.05).

#### 2.8.3 Search for marker proteins for HELs

To investigate further the function of HELs, a targeted analysis was carried out on candidate marker proteins. To do this, genes and proteins upregulated in the HF (highly enriched in HELs) were extracted to conduct functional analyses. For transcriptomics analysis, genes having an FC value >2 were selected, whereas for proteomics analysis, proteins with an FC value >5 were selected, considering the need for a more stringent selection due to the lack of replicates. For this analysis, proteins annotated by several genes were considered, but only if the first three annotations were consistent with each other (i.e., the corresponding unigene having the same Nr annotation or coding for proteins of the same family, representing 98.7% of the identified proteins with a peptide count >2). The coding peptide sequences were obtained from the transcriptome using the TransDecoder software (V3.0.1). The resulting proteins were aligned against the proteome of the species *Apostichopus japonicus* for protein–protein network analysis using STRING-DB V12.0. The interactome significance was assessed using the PPI p-value in STRING. Moreover, the protein networks obtained by MS and RNA-seq analyses were compared by looking at the significantly enriched biological processes (from the Gene Ontology database) shared between the two analyses (FDR < 0.05). In addition, a string local network analysis was used to highlight functional protein clusters of interest (FDR < 0.05). In this case, the expression and annotation of proteins identified in the cluster were further analysed using a heat map visualisation carried out in MetaboAnalyst V6.0 (log-normalised FPKM after t-test filtering). The annotation displayed corresponds to the gene Nr annotations, but the corresponding STRING annotation is shown in **Supplementary Table 9**. Finally, to emphasise the most specific proteins in HELs, the 15 proteins having the highest fold change value in the MS analysis were listed with their annotation and peptide count as an annotation proxy (i.e., the higher the peptide count is, the more reliable the gene annotation is).

As a complementary analysis, a search was carried out for the expression of globin, in line with the previous hypothesis about HEL pigmentation (39, 44). To do this, a local tBlastn was performed using BioEdit V7.7.1 on the whole transcriptome (E-value <10^−5^) based on four existing peptide sequences coding for globin in echinoderms and reported as intracellular in coelomocytes (48). Obtained unigenes were blasted again using Blastx and were only considered if the first hit matched the annotation of “globin”. The resulting unigenes were finally searched in the DEG list between PF and HF (i.e., |log_2_FC value| ≥1 and an FDR ≤0.05), and their expression was visualised using a heat map in MetaboAnalyst V6.0 (based on the FPKM log-normalised value). A search for proteins annotated with these genes was also carried out in the MS protein list to compare their expression between PF and HF samples.

### 2.9 Redox analysis

To investigate the antioxidant properties of HELs, we (1) looked at the expression of genes involved in ROS reduction and (2) analysed ROS production among the different coelomocyte populations by flow cytometry and fluorescent microscopy using a ROS marker.

#### 2.9.1 Expression of antioxidant gene

To check if antioxidant genes could be overexpressed in HELs, a search was carried out in the transcriptome for genes known to have functions in ROS reduction, including genes annotated as “superoxide dismutase” (SOD), “catalase” (CAT), and “glutathione peroxidase” (GPx) (49). We first checked if some of these genes were significantly differentially expressed between PF and HF based on differential transcriptomics analysis (see section 3.5). Then, in order to study the expression of these antioxidant genes as a functional entity, the expression of all of these genes that were identified in the transcriptome was summed (sum of FPKM values) within each gene family (i.e., for SOD, CAT, and GPx) as well as for their total (i.e., sum of FPKM value of the genes from the three families). These were compared between PF and HF samples using a Wilcoxon signed-rank test in R V4.2.2 (α = 0.05; n = 6). To further visualise the gene expression of the gene of interest (i.e., antioxidant genes), a heat map was carried out based on FPKM values in the MetaboAnalyst (V6.0) server (log(FPKM)). Finally, a relation was tested between the ratio of PF expression out of HF expression as a function of HEL proportion in the HF for each individual to determine whether the proportion of HELs influences the antioxidant gene expression. This relation was tested using a Pearson's correlation test (α = 0.05) after checking the normality of data using a Shapiro–Wilk test, R V4.2.2 (α = 0.05).

#### 2.9.2 ROS production analysis

To estimate the ROS production in the different coelomocyte populations, 2′,7′-dichlorodihydrofluorescein diacetate (DCFH-DA) was used as a marker of intracellular ROS (Merck; D6883). DCFH-DA is a cell-permeable non-fluorescent probe that forms fluorescent DCF when oxidised by intracellular ROS, having an excitation wavelength of 485 nm and an emission range between 500 and 600 nm (50). ROS production was compared in coelomocyte populations from the two body fluids exposed or not to LPS. Four individuals were used for this experiment. Their fluids were collected and mixed directly with CMFSW + EDTA. Each fluid was then separated into two tubes: one for the LPS condition and one for the control condition. The cells were then counted, pelleted using centrifugation for 5 min at 500 × g and 4 °C, and resuspended in coelomocyte culture medium (CCM; 0.5 M NaCl; 5 mM MgCl_2_; 1 mM EGTA; 20 mM HEPES; pH 7.2, as per Smith et al. (21) to obtain a concentration of 5 million cells per ml. The CCM of treated samples contained a final concentration of 20 µg per ml of LPS from *Escherichia coli* O111:B4 (Merck; L2630). After incubation for 1 h, each sample was separated into three tubes corresponding to the three labels: a sample without labelling, a sample with 1 µg/ml of propidium iodide (PI—a marker of cell death; Merck; P4170), and a sample with 5 µM of DCFH-DA (final concentrations). DCFH-DA was first added to the corresponding samples. Samples were incubated again for 30 min, and the cells were washed and resuspended in the flow cytometry buffer (see section 2.6). PI was added to the samples 5 min before the analysis. The different samples, corresponding to different fluids conditions, and labelling were analysed using the Sony ID7000™ Spectral Cell Analyser. First, a weighted least squares method algorithm implemented in the ID7000 system was applied: Each fluorochrome was visualised on a dot plot alongside the other fluorochromes to assess potential unmixing inaccuracies. Moreover, the Sony ID7000™ Spectral Cell Analyser provides a tool called Autofluorescence Finder (AF), which allows for the identification and characterisation of AF signatures within complex multicolour samples. Once AF spectra are identified, they are processed and mathematically separated as independent fluorescence parameters (named AF). Data for analysis were gated on live cells, and manual adjustments were made when necessary, before loading the result on FlowJo V10.10.0 for cell analysis. Cell populations were gated following the same strategy as in section 2.6. For each population, ROS-positive cell and dead cell proportions were calculated based on discrimination with unlabelled samples, using the FITC and PI channels, respectively. Moreover, ROS production was calculated using the median fluorescence in DCFH-DA-marked samples. To highlight significant differences between populations, a Friedman statistical test was performed in R V4.2.2, with the Dunn test as a *post-hoc* test (only considering LPS-treated samples; n = 4; α = 0.05). Moreover, differences between LPS and CON samples were tested using the Mann–Whitney U test in R V4.2.2 (n = 4 in the LPS condition and n = 3 in the control condition). Finally, to confirm the results obtained by flow cytometry, samples were observed under an epifluorescent microscope using a FITC filter (excitation wavelength = 495 nm; Zeiss Axio 1). The level of ROS positivity was qualitatively (i.e., based on fluorescence intensity) compared between the coelomocyte populations by comparing them using the same fluorescence intensity.

## 3 Results

### 3.1 HF and PF have distinctive coelomocyte populations

**Figure 1:**
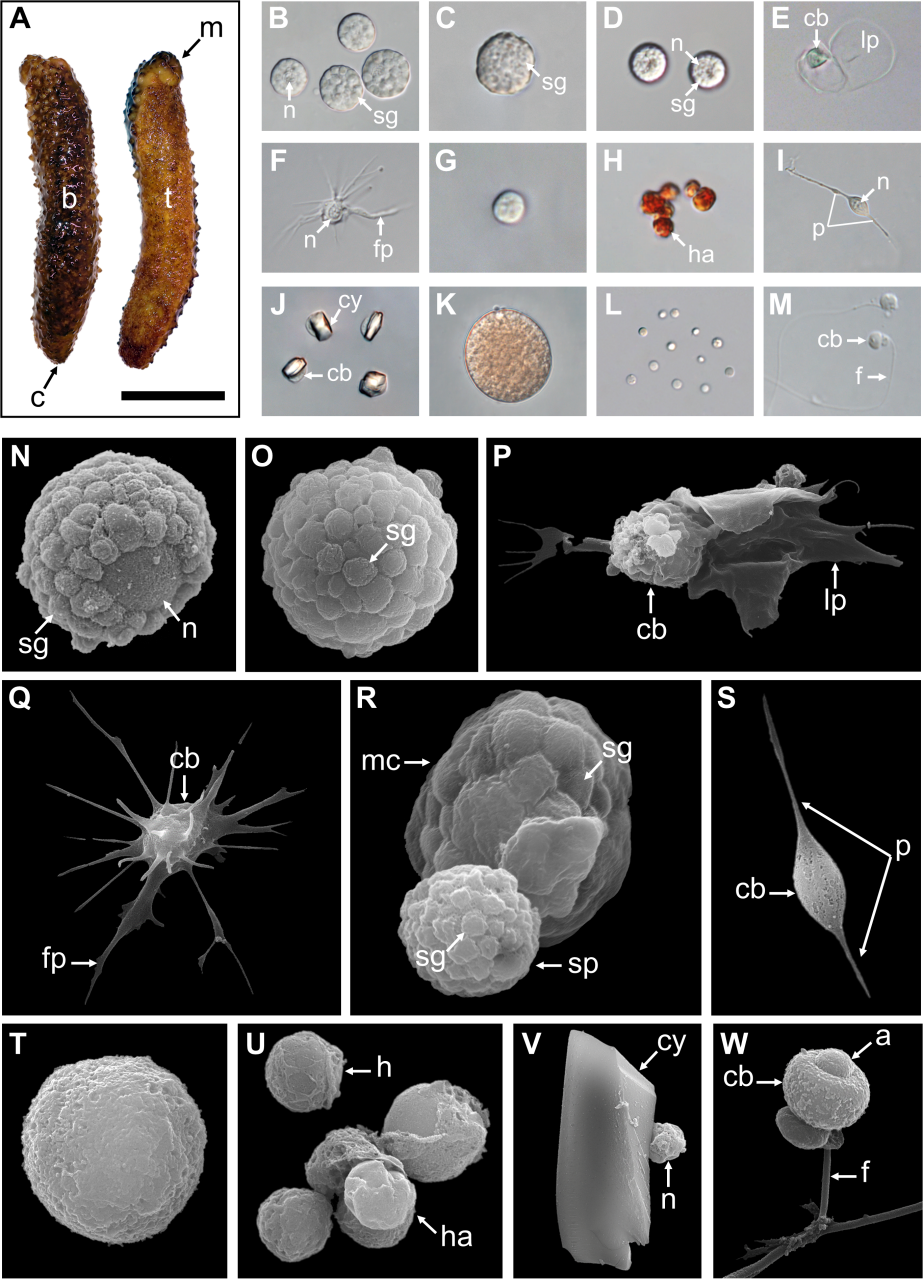
Cellular elements found in the perivisceral and hydrovascular fluids of *Holothuria forskali*. **(A)** Aboral and oral views of a specimen of *H*. *forskali*. B-M. Light microscopy on coelomocytes: **(B)** Morula cells. **(C)** Large spherulocyte. **(D)** Small spherulocytes. **(E)** Petaloid phagocyte. **(F)** Filiform phagocyte. **(G)** Small round cell (SRC). **(H)** Haemocyte-like cells (HELs). **(I)** Fusiform cell. **(J)** Crystal cell. **(K)** Presumed giant cell. **(L)** Presumed minute corpuscles. **(M)** Spermatozoon. **(N-V)** Scanning electron microscopy on coelomocytes: **(N)** and **(O)** Large spherulocytes. **(P)** Intermediate phagocyte. **(Q)** Filiform phagocyte. **(R)** Side-by-side, a morula cell and a spherulocyte. **(S)** Fusiform cell. **(T)** SRC. **(U)** HELs. **(V)** Presumed crystal cell. **(W)** Spermatozoon. Legend: a, acrosome; b, *bivium*; c, cloaca; cb, cellular body; cy, crystal; f, flagellum; fp, filopodia; h, haemocyte-like cell (HEL); ha, HEL aggregate; lp, lamellipodia; m, mouth; mc, morula cell; n, nucleus; p, pseudopodia; sp, spherulocyte; sg, secretory granules; t, *trivium*. The scale bar (in A) represents 8.5 cm in A; 38 µm in B; 19 µm in C; 24 µm in D; 35 µm in E; 31 µm in F; 17 µm in G; 17 µm in H; 34 µm in I; 32 µm in J; 35 µm in K; 31 µm in L; 26 µm in M; 3 µm in N; 3.4 µm in O; 5 µm in P; 6.2 µm in Q; 3 µm in R; 8.6 µm in S; 2.1 µm in T; 4 µm in U; 4.4 µm in V; 2.5 µm in W.

A total of 12 cellular elements could be distinguished in the HF and PF of *H. forskali* (**Figure 1A**), including three types of spherule cells (morula cells, large spherulocytes, and small spherulocytes), two types of phagocytes (petaloid and filiform phagocytes), small round cells (SRCs), HELs, fusiform cells, crystal cells, giant cells, minute corpuscles, and spermatozoa (**Figures 1B-M**). The different types of spherule cells were divided according to their size and the size of their granules: morula cells were the largest, measuring 14.6 ± 2.8 µm with granules larger than 3 µm in diameter (**Figures 1B, R**); large spherulocytes measured 10.8 ± 1.9 µm (**Figures 1C, N, O**); whereas small spherulocytes measured 6.5 ± 1.1 µm (**Figures 1D, R**), both with granules between 1 and 2 µm in diameter. Petaloid and filiform phagocytes, measuring 23.1 ± 11.5 µm (including pseudopodia), could be identified based on the morphology of their pseudopodia, harbouring lamellipodia (**Figures 1E, P**) and filopodia (**Figures 1F, Q**), respectively. However, many phagocytes presented pseudopodia of various shapes and were difficult to assign to one or the other subtype. Therefore, all phagocytes were considered as one cell type in the cell count. The SRCs were smaller and had an undifferentiated appearance, measuring 5.3 ± 1.6 µm (**Figures 1G, T**). HELs were also small cells with highly variable diameters ranging from 1.5 to 8 µm, with a mean size of 3.3 ± 1.5 µm (**Figures 1H, U**). These were mainly recognised through their reddish colour (**Figure 1H**) and their propensity to form cell aggregates (**Figure 1U**). Fusiform cells were distinguished based on their two opposite pseudopodia. These cells measured 27.3 ± 8 µm long (**Figures 1I, S**). Crystal cells were recognised through their typical prismatic crystalline inclusion; it should be noted that the SEM image only represents a putative crystal cell, as the crystalline inclusion might also correspond to a methodological artefact, and only a few of them were observed (**Figures 1J, V**). They measured 8.2 ± 1.6 µm. In addition to conventional coelomocyte types, two cell types were observed only a few times; they correspond to giant cells measuring 44.1 ± 5.4 µm with a light-orange pigmentation (**Figure 1K**) and minute corpuscles, which seemed empty with a very regular spherical shape, and a small size of 2.6 ± 0.4 µm (**Figure 1L**). It is uncertain whether these cell types are coelomocytes or the result of tissue contamination or cell debris release. Finally, spermatozoa could also be distinguished only in some male individuals and were sometimes very abundant in the two body fluids. They were identified mainly by their flagella (**Figures 1M, W**) and their characteristic acrosome, conspicuous in SEM preparations (**Figure 1W**).

Only phagocytes, spherulocytes (large and small merged), morula cells, SRCs, HELs, fusiform cells, and crystal cells were considered in cell concentration and proportion assessment as they were almost always present and easily distinguishable under light microscopy. In total, HF contained 1.40 ± 1.0 × 10^7^ cells per ml, which was significantly higher than PF, containing 3.28 ± 0.8 × 10^6^ cells per ml (**Table 1**; Wilcoxon signed-rank test; p = 0.030; W = 21; **Supplementary Table 1** shows the full statistical analysis results). This difference was mainly driven by the high concentration of HELs in HF, with an average concentration of 1.20 ± 1.0 × 10^7^ cells per ml and a proportion of 80.6 ± 5.1%. Comparatively, HELs were particularly poorly represented in the PF, with a proportion of 0.8% ± 0.7%, and were only observed in four of the six individuals considered for this assessment. Although HELs were overall almost absent in the PF of *H. forskali*, they could reach 20% in some individuals examined. In the PF, spherulocytes were the dominant cell type with a concentration and proportion of 1.2 ± 1.0 × 10^6^ cells per ml and 36.0 ± 5.9%, respectively. Concentrations and proportions of HELs and spherulocytes were significantly different between the two fluids (p = 0.031; W = 21; **Table 1**). Phagocytes were the second dominant cell type in the two body fluids, and their concentration was similar at around 10^6^ cells per ml, with a proportion of 10.5 ± 11.3% in the HF and 30.0 ± 10.1% in the PF. Next, SRCs and morula cells had similar concentrations between the two fluids at around ~1.2 × 10^6^ cells per ml and ~6 × 10^6^ cells per ml (**Table 1**). Finally, fusiform and crystal cells were both poorly represented, with fewer than 10^5^ cells per ml. Interestingly, crystal cells were significantly more concentrated in the PF at 2 ± 0 × 10^4^ cells per ml against 3.3 ± 8.2 × 10^3^ cells per ml in the HF (p = 0.037; W = −15). Overall, proportions were significantly different between the two fluids in all the cell types (p < 0.05) except for fusiform cells. However, these different proportions can be attributed to the high concentration of HEL, which only occurs in HF.

**Table 1:** Comparison of concentration and proportion for each cell population between the hydrovascular fluid (HF) and perivisceral fluid (PF), in specimens under normal homeostatic conditions (i.e., uninjected specimens).

Cœlomocyte types	Concentration (cells ml^−1^)			Proportion (%)		
	HF	PF	P-value	HF	PF	P-value
Phagocyte	9.28 ± 6.18 × 10^5^	1.04 ± 0.45 × 10^6^	ns	10.52 ± 11.33	30.98 ± 10.07	*
Spherulocyte	3.27 ± 1.44 × 10^5^	1.19 ± 0.41 × 10^6^	*	2.91 ± 1.45	36.04 ± 5.90	*
Morula cell	1.05 ± 1.28 × 10^5^	1.41 ± 1.16 × 10^5^	ns	1.07 ± 1.12	5.05 ± 3.14	*
Small round cell	5.78 ± 4.16 × 10^5^	7.37 ± 1.61 × 10^5^	ns	4.49 ± 2.69	23.44 ± 7.36	*
Haemocyte-like cell	1.20 ± 0.96 × 10^7^	2.33 ± 2.34 × 10^4^	*	80.62 ± 5.14	0.75 ± 0.71	*
Fusiform cell	5.67 ± 5.99 × 10^4^	1.00 ± 1.37 × 10^5^	ns	0.38 ± 0.35	3.09 ± 3.75	ns
Crystal cell	3.33 ± 8.16 × 10^3^	2.00 ± 0 × 10^4^	*	0.01 ± 0.03	0.64 ± 0.15	*
Total (all types)	1.40 ± 0.99 × 10^7^	3.28 ± 0.83 × 10^6^	*	na	na	na

Results are formulated as mean ± SD (n = 6), and p-values show significant differences between the two fluids (Wilcoxon signed rank test; * p-value ≤ 0.05; ns means not significant; na means not applicable; see **Supplementary Table 1** for full results).

### 3.2 Coelomocytes aggregate upon fluid collection

**Figure 2:**
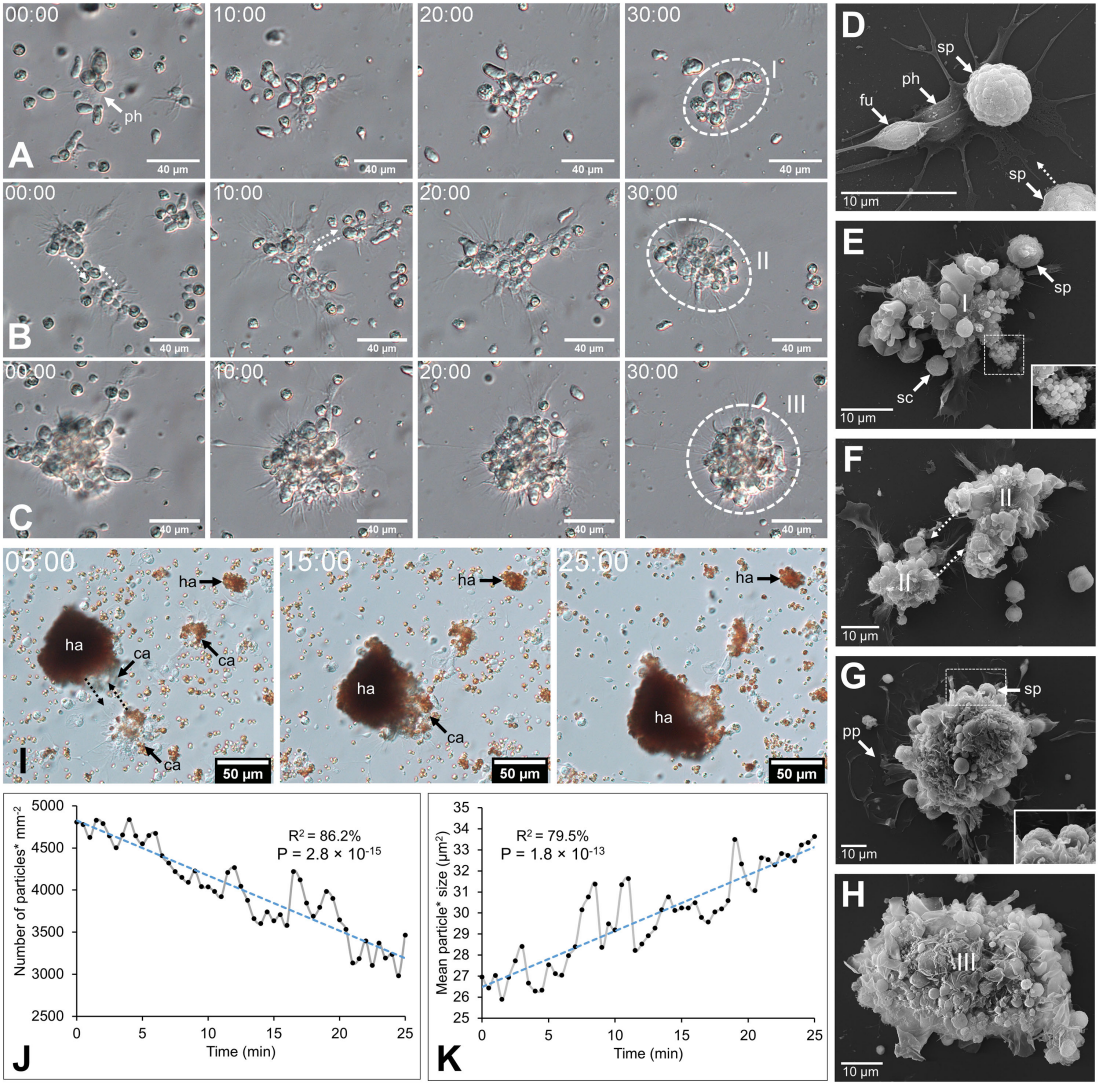
Coelomocyte aggregation following fluid collection in *Holothuria forskali*. A-C. Time-lapse images of early aggregate formation in the perivisceral fluid (PF). **(A)** Formation of stage I aggregate is driven by a retrograde movement of cells encountering a phagocyte and their pseudopods (**Supplementary Video 2**). **(B)** Maturation of a stage I aggregate in a stage II early aggregate (**Supplementary Video 3**). **(C)** Maturation of a stage II aggregate in a stage III aggregate (**Supplementary Video 4**) D-H. SEM images of coelomocytes aggregating in the PF, following what is observed by time-lapse imaging. **(D)** Adherent phagocytes interact with other cells and lead to the formation of the initial aggregate (i.e., only a few cells). **(E)** Other cell types are recruited, notably spherule cells, which release secretory granules through cell lysis (close-up view; **Supplementary Video 1**). **(F)** Fusion between two aggregates of stage II. **(G)** Large compact aggregate of stage II, still showing numerous pseudopodia (with a close-up view of spherule cells taking part in the aggregate). **(H)** Maturation of the aggregates, which become more compact and finally detach from the slide by retracting their pseudopod. **(I)** Time-lapse images showing early aggregate formation in the hydrovascular fluid with HELs and HEL aggregates being passively captured in the early aggregates (**Supplementary Video 5**). **(J)** and **(K)** Respectively, the number of small particles (* means cells with an area between 4 and 50 µm^2^; size targeting HELs; **Supplementary Figure 4B**) and the mean size of particles (* means cells and cell aggregates with an area superior to 4 µm^2^; **Supplementary Figure 4C**) per minute during 25 min (coefficient of determination—R^2^ and Mann–Kendall trend test p-value are included in the graphs). Legend: ca, coelomocyte aggregate; fu, fusiform cell; ha, HEL aggregate; I, stage I aggregate; II, stage II aggregate; III, stage III aggregate; ph, phagocyte; pp, pseudopodia; sc, small round cell; sp, spherule cell.

Coelomocytes appeared to display high mobility after fluid collection and gathered, leading to cell aggregation. In both body fluids, this process began with a phagocyte settling on the slide and extending its pseudopodia (**Figures 2A, D**). Then, some previously immobile coelomocytes started to achieve amoeboid movements and join the settled phagocytes, especially spherule cells that could suddenly migrate toward the aggregate by forming characteristic small spherical protrusions followed by an undulation throughout the cell membrane, giving them a figure-eight appearance (**Supplementary Figure 3B**; **Supplementary Video 1**). In some cases, when they encountered an aggregate, spherule cells could perform a sudden cell lysis, releasing their cytosol content, including numerous secretion granules within the aggregate (**Supplementary Video 1** arrow; **Supplementary Figure 3B****;****Figure 2E**). Other cells were passively captured in the newly formed aggregate when encountering it through the fluid flow. This process results in the formation of early, disorganised, and loose stage I aggregates (**Supplementary Video 2****;****Figures 2A, E**). These aggregates, by a retrograde movement of the phagocytic pseudopodia, while capturing additional cells, became more compact. If some early aggregates were close together, they could merge by a reciprocal tractive force of the pseudopodia (**Figures 2B, F**). This results in aggregates at stage II with many pseudopodia scattered around them (**Supplementary Video 3****;****Figures 2B, G**). As the aggregates grew, the pseudopodia became more stretched, and some were gradually retracted within the aggregates. Aggregates also acquired a spherical shape and slowly detached from the slide, resulting in stage III aggregates (**Supplementary Video 4****;****Figures 2C, H**). These aggregates were capable of autonomous lateral movement on the slide. While the aggregation process was the same in the two body fluids, many HELs were passively captured in early aggregates in the HF. Most HELs were isolated, but many formed small aggregates composed solely of HELs. **Figure 2I** shows a large HEL aggregate surrounding an early aggregate, itself fusing with another early aggregate, all capturing isolated HELs and other coelomocytes (**Supplementary Video 5**). In the upper right of the video, a small HEL aggregate remains immobile without the action of a phagocyte or another early aggregate. To confirm that HELs participate in the aggregate, even passively, a quantitative particle analysis was performed by automatically counting cells whose surface area is between 4 and 50 µm^2^, targeting HEL size. This analysis shows that the number of isolated HELs decreased significantly throughout the time lapse, indicating that HELs join the aggregates over time (**Figure 2J**; Mann–Kendall trend test: z = −7.9; p = 2.8 × 10^−15^). However, this does not necessarily mean that this decrease in free HELs results from an active movement of these cells towards the early aggregates. Indeed, the time-lapse video suggests that it was rather the fluid flow that caused their movement, but that once they encountered an aggregate or another cell, they strongly adhered to it (**Supplementary Video 5**). Similarly, the mean aggregate size was followed during time lapse, and it appears that it increased significantly over time (**Figure 2K**; z = 7.3; n = 51; p = 1.8 × 10^−13^). This can be explained by the fact that isolated cells join aggregates and small aggregates merge together, resulting in fewer but larger aggregates. The automated numbering method of HELs and measurement of aggregate size can be found in **Supplementary Figure 4**.

### 3.3 Only HELs are increased 1 day after immunostimulation with LPS

**Figure 3:**
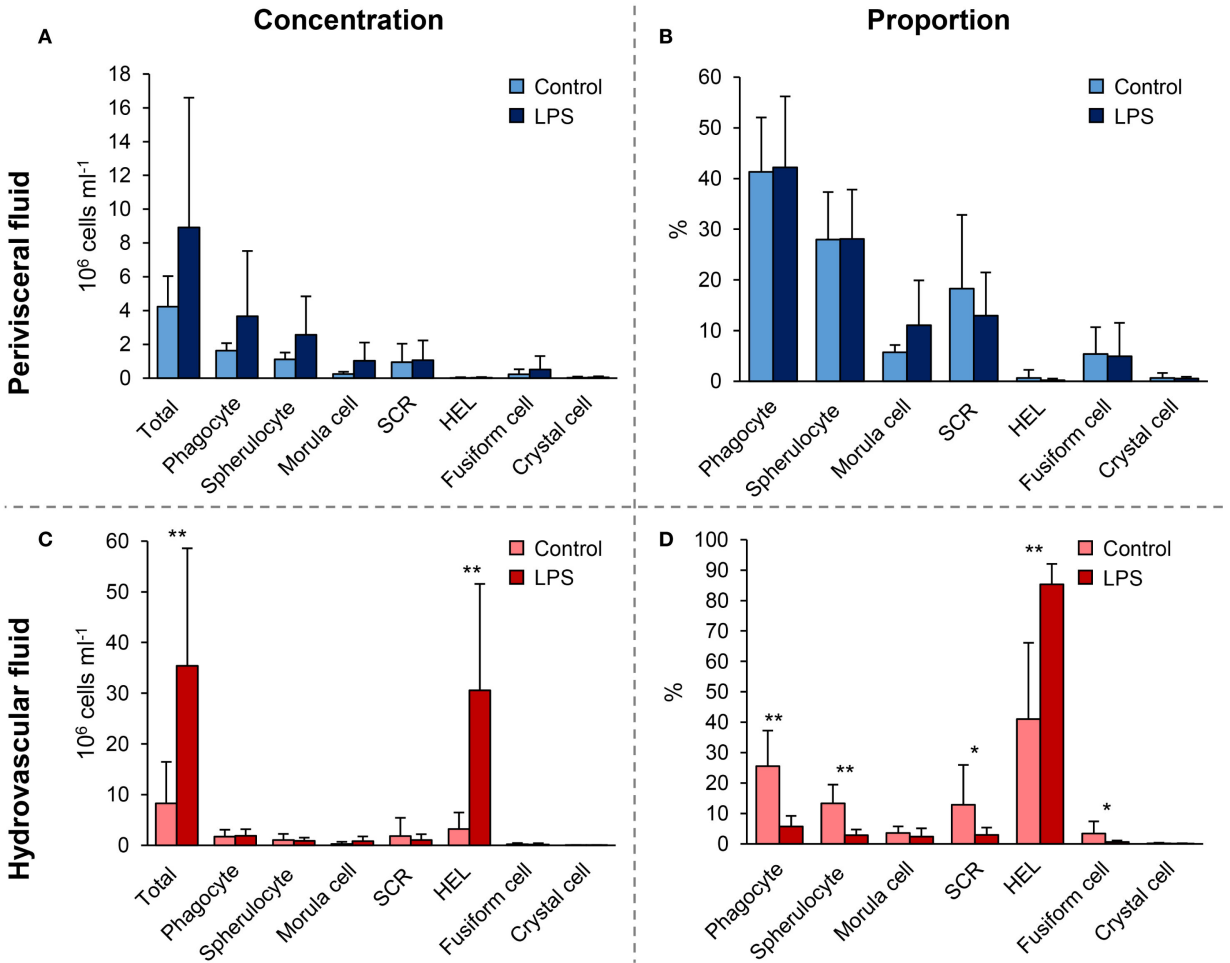
Comparison of cell population concentrations and proportions 24 h after injections of lipopolysaccharides (LPS) between control-injected individuals (artificial sterile seawater; control) and LPS-injected individuals (same solution containing 5 mg ml^−1^ of LPS). **(A)** and **(B)** Cell concentrations in hydrovascular fluid (HF) and perivisceral fluid (PF), respectively. **(C)** and **(D)** Cell proportions in HF and PF, respectively. Scale bars represent the SD (n = 7 in the LPS condition and n = 6 in the control condition), and asterisks show significant differences (Mann–Whitney U test; * p ≤ 0.05; ** p ≤ 0.01; results of the statistical analysis are shown in **Supplementary Table 2**).

Significant changes in cell populations following LPS injections were only found in the HF (**Figure 3**). Firstly, in terms of concentration, the total number of coelomocytes and HELs differed significantly, with a clear increase in LPS-injected individuals (**Figure 3A**; Mann–Whitney U test; p < 0.01). In PF, although an overall rise in the concentration of all coelomocyte types was observed in LPS-injected individuals, none were significant (**Figure 3B**; p > 0.05). Regarding proportions in HF, most cell types differed significantly, except morula cells and crystal cells (**Figure 3C**; p < 0.05). Only HELs increased in proportion in LPS-injected individuals, with a proportion of 85.3 ± 6.6% in LPS-injected against 41 ± 25.1% in control individuals (p = 4.7 × 10^−3^; U = 2). As only HELs change in concentration in the HF, this increase explains why the proportion of other cell types is significantly lower. Finally, the proportions in the PF were stable between control and LPS-injected individuals, except for morula cells and SRCs, which showed a slight increase and decrease in LPS-injected individuals, respectively, although these were not significant (**Figure 3D**; p > 0.05). Proportion and concentration values, as well as the results of the statistical tests, can be consulted in **Supplementary Table 2**.

### 3.4 Coelomocytes have a specific gene expression profile following a LPS challenge

#### 3.4.1 Transcriptome metrics and quality control

A total of 14 cDNA libraries were sequenced, including six HF cell samples, six PF cell samples, one stone canal sample, and one podia sample sequenced for other projects (**Supplementary Table 3**). They generated 3.33 billion reads with 93.0% clean reads and a Q20 of 99.0%. The *de novo* assembly yielded a total of 167,199 unigenes ranging from 30,861 to 73,673 unigenes for individual samples and with an average length, an N50, and a GC content of 2,017, 3,807 and 38.9%, respectively.

The unigene length distribution showed that most unigenes were between 300 and 3,000 bp in length (76.2%; **Supplementary Figure 5A**). To obtain an initial indication of the function of the unigenes, the transcriptome was aligned with seven functional databases; 45.5% had at least one annotation, with Nr having the highest proportion of annotated unigenes (41%), followed by KEGG (31.7%) and InterPro (30.5%; **Supplementary Figure 5B**). For the Nr annotation, the distribution of species shows a majority of echinoderm species (>77.8%), with the most represented species being *Apostichopus japonicus* (60.4%; **Supplementary Figure 5C**). To assess the completeness of the transcriptome, BUSCO analyses were used; while individual transcriptomes showed a variable degree of completeness, the merged transcriptome showed a high percentage of complete orthologs (97.2%), most of which are duplicated (75.6%) (**Supplementary Figure 5D**).

#### 3.4.2 Differential gene expression between control and LPS individuals

**Figure 4:**
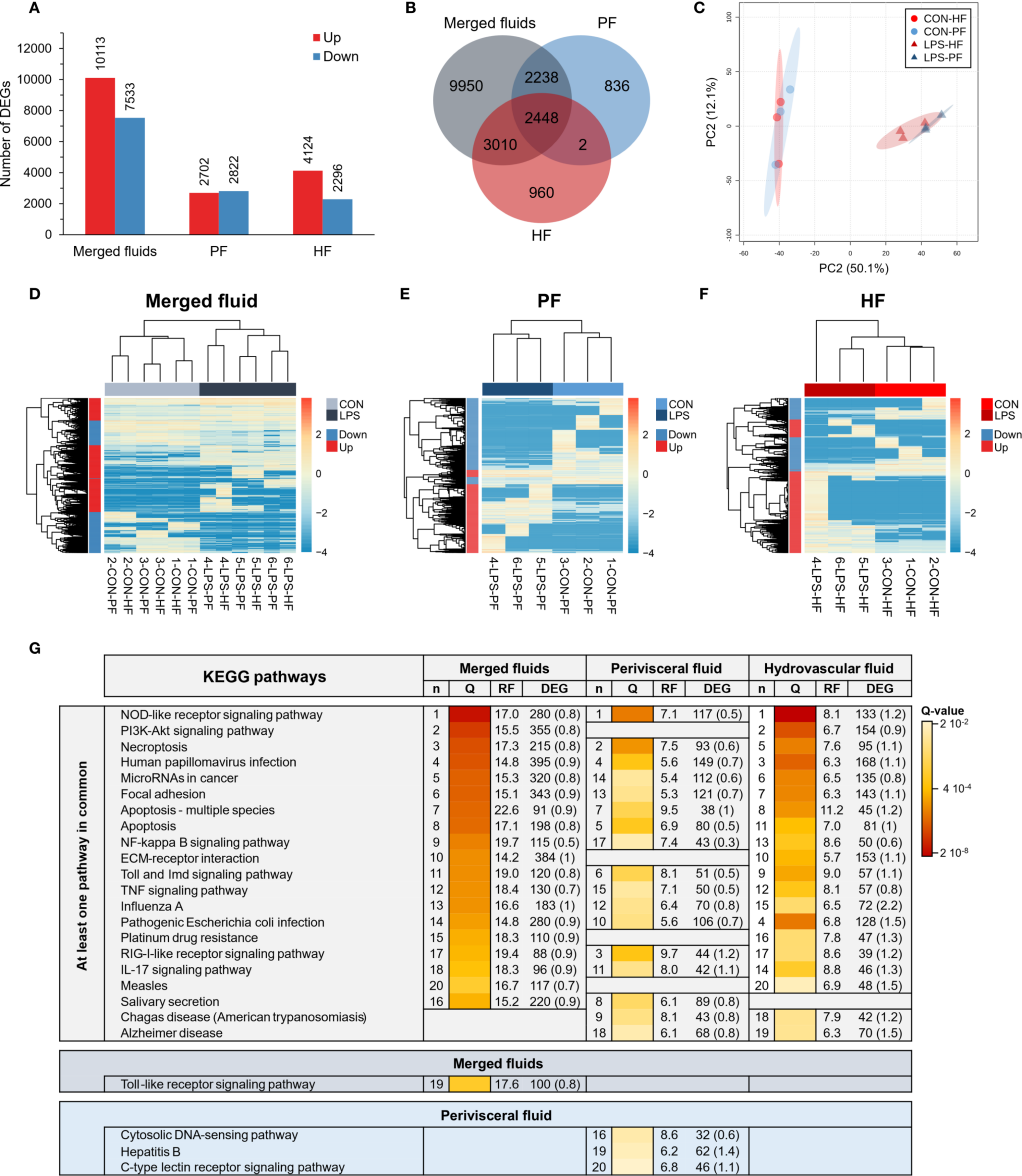
Differential expression analyses between control-injected (CON) and lipopolysaccharide-injected (LPS) individuals of *Holothuria forskali*. **(A)** Number of up- and downregulated genes between CON and LPS individuals in the three analyses carried out: the first considering all body fluids; the second only considering the perivisceral fluid (PF), and the third only considering the hydrovascular fluid (HF) (the exact number of differentially expressed genes (DEGs) for each analysis is included in the graph; the full list is shown in **Supplementary Table 4**). **(B)** Venn diagram of DEGs between the three analyses. **(C)** Principal component analysis (PCA) of DEG expression considering the analysis of CON vs. LPS (i.e., considering all body fluids). **(D-F)** Heat maps based on the three differential expression analyses following the same order as in **(A, G)** The 20 most enriched KEGG pathways in the three analyses; pathways were ordered so that the degree of enrichment can be compared between the three analyses; n represents the ranking of the pathway in each analysis; q-value (Q) represents the significance of the enrichment according to a colour scale; rich factor (RF) represents the percentage between the number of DEGs out of the number of unigenes in a given pathway; DEG represents the number of DEGs annotated in the pathways and, in brackets, is indicated the ratio between up- and downregulated within these DEGs (CON versus LPS). The full list of KEGG enrichment analyses between CON and LPS individuals can be found in **Supplementary Table 5**.

Three differential analyses were performed to compare expression between control and LPS-injected individuals; one considered only HF samples (HF analysis), one only PF samples (PF analysis), and one both (merged fluid analysis). The merged fluids analysis yielded a total of 17,646 DEGs of which 10,113 genes were upregulated and 7,533 downregulated; the PF analysis yielded 5,524 DEGs of which 2,702 genes were upregulated and 2,822 downregulated and the HF analysis yielded 6,420 DEGs of which 4,124 genes were upregulated and 2,296 downregulated (**Figure 4A**). Comparing the three analyses in a Venn diagram shows that 2,450 DEGs were common between PF and HF analysis with only 2 not found in the merged fluid analysis (**Figure 4B**). A PCA was performed on the DEGs from the merged analysis, showing a clear distinction between control and LPS-injected individuals with a higher variability within LPS-injected individuals (**Figure 4C**). For each analysis, a heat map was produced based on the DEGs (**Figures 4D-F**). While the merged and PF analyses show a good clustering between control and LPS-injected individuals, in the HF analysis, the 4-LPS-HF sample forms an individual branch, possibly due to a sex influence, as it was the only male in the LPS-injected condition (**Supplementary Table 3****;****Supplementary Figure 6**). Furthermore, in the merged analysis group, clustering reveals a strong influence of individuality rather than sex or body fluids (i.e., the HF-PF distinction). To obtain a functional overview of the DEGs, the 20 most enriched KEGG pathways were selected for each analysis, and a comparison was performed to reveal potential specific pathways in the immune response of each body fluid (**Figure 4G**). Of the 20 most enriched pathways, 14 were common to all three analyses, and 16 were common to the PF and HF analyses. A ranking was established based on the q-value (adjusted p-value), representing the significance of the enrichment for each pathway. All three analyses presented the same most enriched pathways, namely, “NOD-like receptor signalling pathway”, followed by “PI3K-Akt signalling pathway” in the merged and HF analysis and “necroptosis” in the PF analysis. Surprisingly, the “PI3K-Akt signalling pathway” was not among the 20 most enriched pathways in the PF analysis. In addition, the “RIG-I-like receptor signalling pathway”, which was the third most enriched pathway in the PF analysis, was only the 17th most enriched pathway in both the merged and HF analyses. The most represented KEGG category in all three analyses was “Immune system” with a total of six terms. The pathways with the highest rich factors (RF) were “Apoptosis - multiple species” in the merged and HF analyses and “RIG-I-like receptor signalling pathway” in the PF analysis. Regarding the ratio between up- and downregulated DEGs, the highest ratio (i.e., higher expression in the LPS condition) corresponds to “ECM–receptor interaction” and “pathogenic *Escherichia coli* infection” (1) in the merged analysis, to “hepatitis B” in the PF analysis (1.4), and to “influenza A” in the HF analysis (2.2). Overall, these results show a clear response in gene expression to LPS injection, with a similar gene expression between the two fluids, which mainly corresponds to “immune system” pathways.

#### 3.4.3 Sex-specific differential expression

Since a sex influence was observed in the expression of coelomocytes (see section 3.4.2), an additional expression analysis was performed to assess the influence of sex on gene expression (**Supplementary Figure 6**). A large number of DEGs were identified (**Supplementary Figure 6A**), with a clear distinction between male and female samples (**Supplementary Figures 6B, C**). In addition, enrichment analyses revealed that many immune pathways were significantly differentially expressed between the two sexes (**Supplementary Figures 6D-F**). However, it cannot be excluded that this difference is due to the presence of spermatozoa in the male samples.

### 3.5 Coelomocytes exhibit distinct gene expression profiles between HF and PF

**Figure 5:**
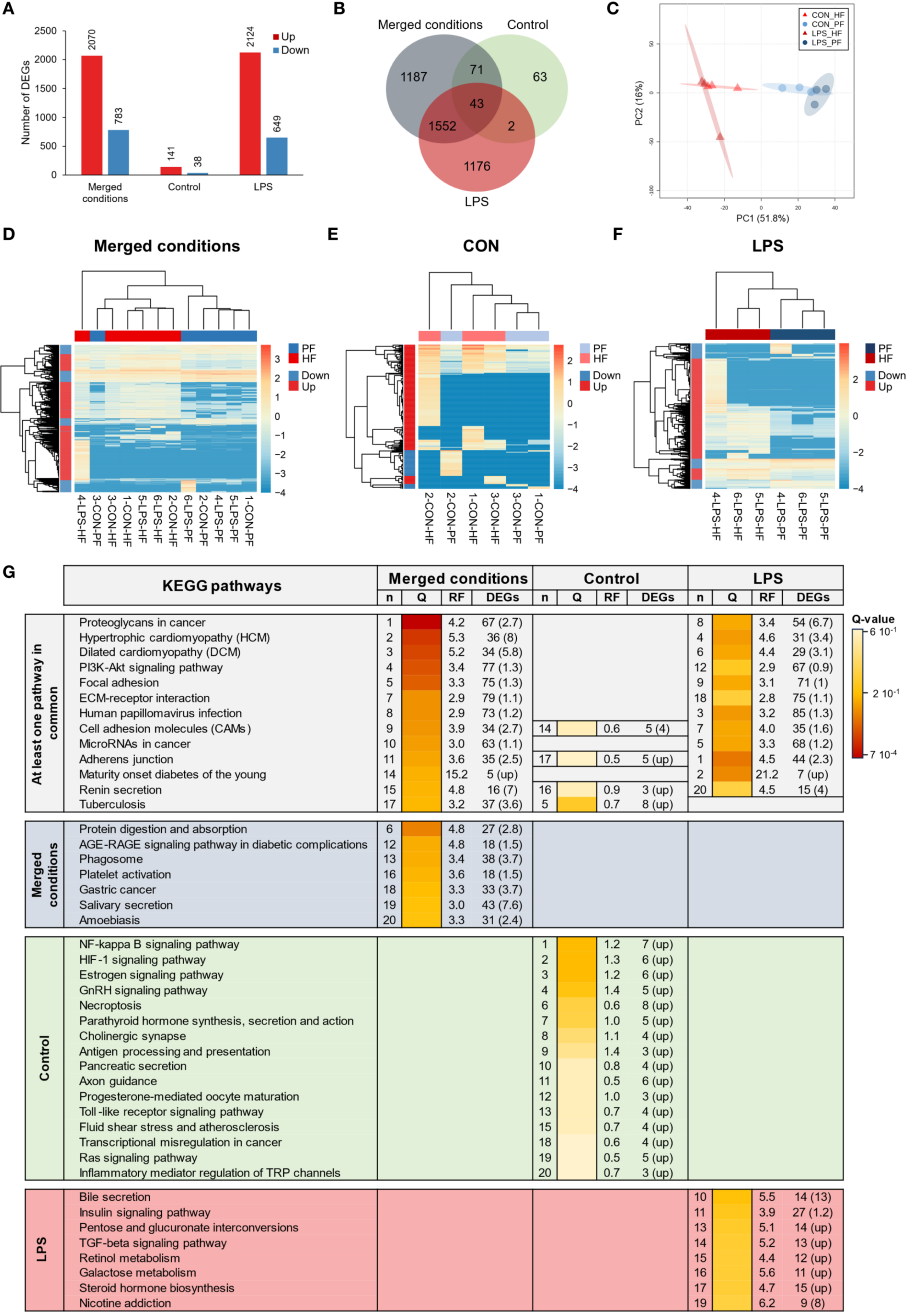
Differential expression analyses between the perivisceral fluid (PF) and the hydrovascular fluid (HF) in *Holothuria forskali*. **(A)** Number of up and downregulated genes between the PF and the HF in the three analyses carried out: the first considering all conditions, the second only considering control individuals (CON), and the third only considering LPS individuals (the exact numbers of differentially expressed genes (DEGs) are included in the graph; the full list is shown in **Supplementary Table 6**). **(B)** Venn diagram of DEGs between the three analyses. **(C)** Principal component analysis (PCA) of DEG expression considering the analysis between PF vs. HF (i.e., considering the two conditions). **(D-F)** Heat maps based on DEGs of the three differential expression analyses following the same order as in **(A, G)** Twenty most enriched KEGG pathways in the three analyses; pathways were ordered so that the degree of enrichment can be compared between the three analyses; n represents the ranking of the path; q-value (Q) represents the significance of the enrichment according to a colour scale; rich factor (RF) represents the percentage between the number of DEGs out of the number of unigenes for a given pathway; DEG represents the number of DEGs annotated in the path and, in brackets, the ratio between up- and downregulated within these DEGs (“up” means that all genes in this pathways were upregulated). The full list of KEGG enrichment analyses between the PF and HF can be found in **Supplementary Table 7**.

To compare expression between PF and HF samples, three differential analyses were performed: one considered only control samples (CON analysis), one only LPS-injected samples (LPS analysis), and one both (merged condition analysis). The merged analysis yielded a total of 2,853 DEGs, of which 2,070 genes were upregulated and 783 downregulated in the HF; the CON analysis yielded 179 DEGs, of which 141 genes were upregulated and 38 downregulated in the HF; and the LPS analysis yielded 2,773 DEGs, of which 2,124 genes were upregulated and 649 downregulated in the HF (**Figure 5A**). The Venn diagram shows that only 43 DEGs were shared by all three analyses, which is mainly due to the low number of DEGs in the CON analysis (**Figure 5B**). Similarly, only 45 DEGs were shared between the CON and LPS analyses, but the LPS and merged analyses shared 1,552 DEGs. The PCA based on the merged analysis showed a clear distinction between the HF and PF samples; however, this separation was less pronounced under control conditions (**Figure 5C**). While the heat maps of merged and LPS analyses show a relatively good distinction between PF and HF, clustering in the CON analysis shows an inaccurate separation (**Figures 5D-F**). With regard to the comparison of the 20 most enriched pathways between the three analyses, only three pathways are shared (**Figure 5G**). However, 12 pathways are shared between the merged analysis and the LPS analysis, consistent with the higher number of DEGs that are shared between these two analyses. The three most enriched pathways were “proteoglycans in cancer”, “hypertrophic cardiomyopathy”, and “dilated cardiomyopathy” in the merged analysis; “NF-kappa B signalling pathway”, “HIF-1 signalling pathway”, and “estrogen signalling pathway” in the CON analysis; and “adherens junction”, “maturity onset diabetes of the young”, and “human papillomavirus infection” in the LPS analysis. In terms of KEGG categories, the most represented system is the “endocrine system” (7 terms), followed by the “digestive system” (4 terms) and the “immune system” (3 terms). The pathways with the highest richness factors (RF) were “maturity onset diabetes of the young” in the merged analysis (1.5%) and LPS analysis (21.2%), and “antigen processing and presentation” (1.4%) in the CON analysis. In terms of the ratio of up- and downregulated genes, many pathways had only upregulated genes in the HF (represented by “up” in **Figure 5G**). Overall, these results show that gene expression between PF and HF coelomocytes is more divergent under immunostimulation with LPS.

### 3.6 Coelomocytes contain carotenoid pigments, which are more abundant in the HF

**Figure 6:**
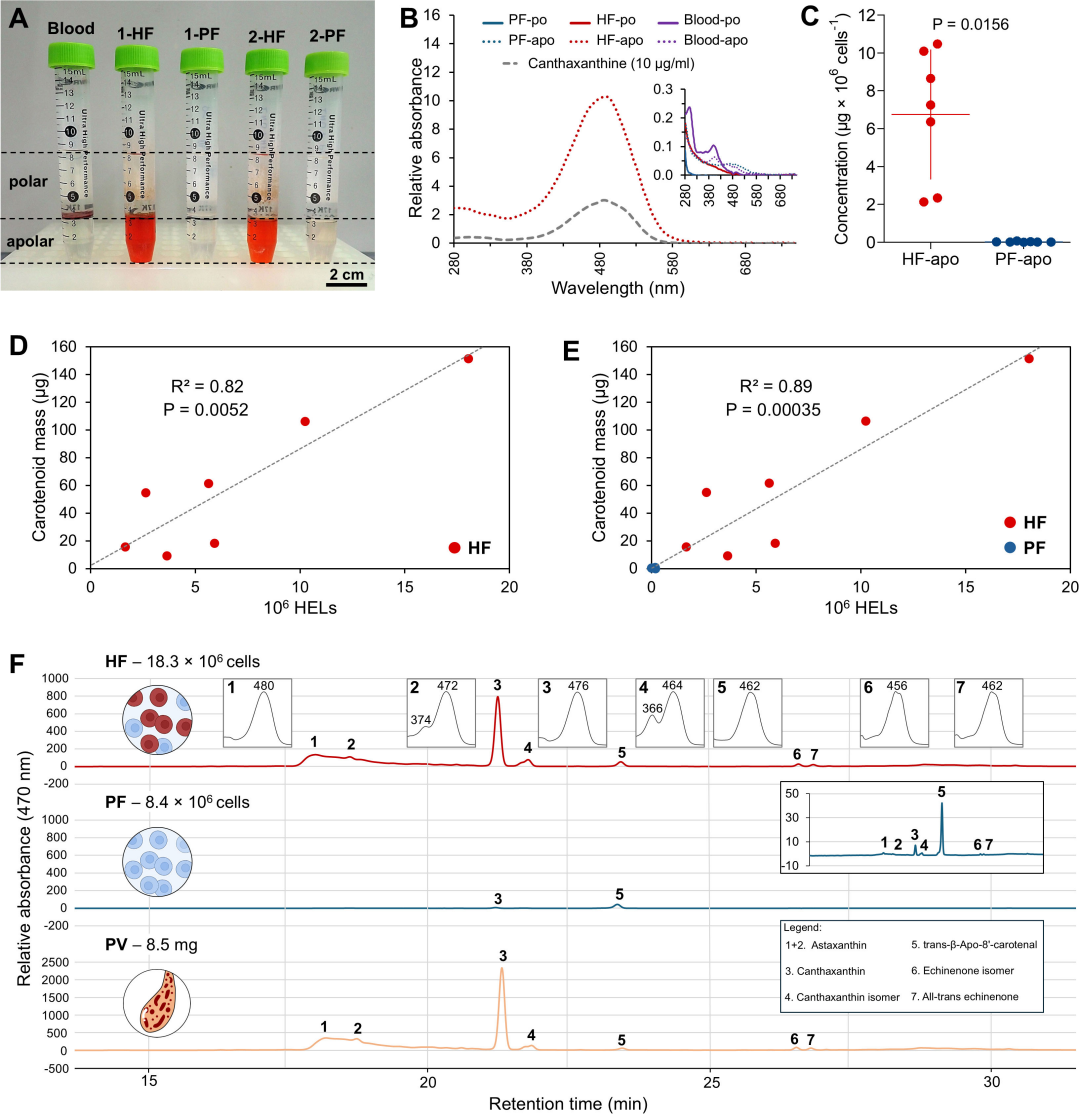
Pigment profiling of coelomocytes from the hydrovascular fluid (HF) and the perivisceral fluid (PF) of *Holothuria forskali*. **(A)** Liquid–liquid extraction of pigment from HF and PF coelomocytes, as well as a bovine blood sample for haemoglobin positive control. The polar phase (methanol + water) surrounds the apolar phase (chloroform). Only the apolar phases of the HF samples show a red pigmentation. **(B)** Absorbance spectra of pigments extracted from the polar and apolar phases of HF and PF samples and from bovine blood (the apolar phase of the PF sample; polar phases and blood samples are shown in the zoomed graph, as their absorbance was too low to be distinguished in comparison with the spectrum of the apolar phase of HF). The spectra were compared to that of a canthaxanthin standard, which peaks at the same wavelength as the apolar phase extract of HF. **(C)** Comparison of the pigment concentration between HF and PF samples calculated based on a calibration curve acquired based on a canthaxanthin commercial standard (λ = 490 nm; **Supplementary Figure 1A**). The masses of pigment are normalised by a million cells (all coelomocyte types included). Scale bars represent the SD, and the p-value is included on the graph (n = 7; Wilcoxon signed-rank test). **(D)** Relation between carotenoid mass and the number of HELs in the HF samples. **(E)** Relation between carotenoid mass and the number of HELs in the PF and HF samples. **(F)** High-performance liquid chromatography (HPLC) profile at 470 nm of HF, PF, and Polian vesicle (PV) tissue samples in *H. forskali* (smaller graph shows absorbance spectra corresponding to each peak). Peaks were identified with commercial standards (**Supplementary Figure 1B**). Peaks 1 + 2 correspond to astaxanthin, peak 3 to canthaxanthin, peak 4 to a canthaxanthin isomer, peak 5 to the injected standard used for quantification (trans-β-Apo-8'-carotenal), peak 6 to an echinenone isomer, and peak 7 to all-trans echinenone (legend: apo, apolar phase; Rel. Abs, relative absorbance; iso, isomer; po, polar phase; PV, Polian vesicle).

A comparative pigment analysis was carried out between the two fluids to characterise the nature of the red pigmentation of the HELs. First, a liquid–liquid extraction was carried out to verify the pigment affinity for polar (water–methanol) or apolar (chloroform) solvents. In addition to the two body fluids (i.e., HF and PF), the analysis includes a mammal blood sample (*Bos taurus*) as a positive control, given the hypothesis that haemocyte pigmentation is due to haemoglobin. The results show that reddish-orange pigmentation is mainly observed in the apolar phase of the HF samples, with some PF samples showing a slight pink colouration in their non-polar phase (**Figure 6A**). The blood sample, in contrast, shows no colouration in the two phases, with most of the colour visible in the interphase, corresponding to pelleted cellular debris in the polar phase (**Figure 6A**).

To determine the nature of the pigments in more detail, the polar and apolar phases of each sample were analysed by spectrophotometry. The spectra corroborate the results obtained visually with a much higher absorbance in the apolar phase of the HF samples, showing a large relative absorbance peak at 490 nm corresponding to a red resulting colour (**Figure 6B**). With regard to the property of pigment (apolar and orange red colour), those measures were associated to the detection and identification of carotenoid pigments by HPLC(see below). Canthaxanthin, being the most abundant carotenoid, was used as a standard in spectrophotometry and shows a very similar spectrum to the HF apolar samples (**Figure 6B**). Using a calibration curve (**Supplementary Figure 1A**), it was possible to reveal a significant difference in the carotenoid concentration between the two fluids, with 6.8 µg ± 3.4 µg per 10^6^ cells in HF compared with 2.6 ± 2.3 × 10^−2^ µg per 10^6^ cells in PF (Wilcoxon signed-rank test: p = 0.0156; W = −28; **Figure 6C**). These concentrations appear consistent with the proportion of HELs in the two fluids, which were 77.3 ± 14.2% in HF and 0.8 ± 0.7% in PF (n = 7). In this way, the carotenoid mass was correlated with the number of HELs only in the HF samples (**Figure 6D**), and in the PF and HF samples (**Figure 6E**), which revealed strong relationships with high coefficients of determination (R^2^) of 89.3% and 81.6%, respectively (Spearman's correlation test: S = 82.6; p = 3.5 × 10^−4^ in D; Pearson’s test: df = 5; t = 4.7; p = 5.2 × 10^−3^ in E).

In parallel with the quantification of carotenoids, HPLC analysis was used to identify the types of pigments present in the two different body fluids as well as in the Polian vesicle (PV) tissue samples (rich in large red bodies; see section 3.7). It reveals the presence of different types of carotenoids, including, in decreasing order of abundance, canthaxanthin and its isomer, astaxanthin, and echinenone and its isomer (**Figure 6F**). **Table 2** shows the concentration and proportion of each type of pigment calculated based on the standard of known concentration (trans-β-Apo-8'-carotenal; peak 5; **Figure 6F**). The most abundant ones, namely, canthaxanthin and astaxanthin, accounted for approximately 55% and 45%, respectively, in the various samples (including the isomer). Furthermore, the concentration of astaxanthin and canthaxanthin appears to be highly correlated in both fluids, as evidenced by a linear relationship with a high coefficient of determination (R^2^ = 99%: **Supplementary Figure 7**). The proportion of echinenone was less than 5% in the HF and Polian vesicle samples, with a slightly higher proportion in the PF at 7.76 ± 4.8% (including the isomer). Importantly, while the proportion of pigment was relatively constant between samples, its concentration was much higher in HF than in PF, corroborating the results obtained by spectrophotometry. However, it should be noted that the total carotenoid content calculated by HPLC is higher than the one obtained by spectrophotometry (e.g., for HF samples: 28.7 ± 9.6 µg per 10^6^ cells by HPLC compared with 6.8 µg ± 3.4 µg per 10^6^ cells by spectrophotometry), which could be explained by the different sensitivity of the two techniques. The PV samples are also very rich in carotenoids (i.e., the carotenoid profile and the relative absorbance were similar to that of the HF samples; **Figure 6F**). Their total carotenoid content was 0.3 ± 0.1 µg per µg of dry mass, thus representing approximately 30% of the total dry mass of the samples.

**Table 2:** Concentrations and proportions of carotenoids measured by high-performance liquid chromatography (HPLC) in the hydrovascular fluid (HF), the perivisceral fluid (PF) and the Polian vesicle (PV) of *Holothuria forskali*.

Peak	Pigment	Concentration (µg × 10^6^ cell^−1^)		Concentration (µg × µg^−1^)	Proportion (%)		
		HF	PF	PV	HF	PF	PV
1 + 2	Astaxanthin	13.1 ± 8.4	0.6 ± 0.8	1.5 ± 1.1 × 10^-2^	42.1 ± 8.1	30.4 ± 9.8	43.4 ± 6.8
3	Canthaxanthin	13.2 ± 7.1	0.7 ± 0.7	1.6 ± 0.8 × 10^-1^	49.3 ± 8.3	48.8 ± 8.8	48.5 ± 6.1
4	Canthaxanthin isomer	1.5 ± 0.9	0.2 ± 0.2	1.8 ± 1.1 × 10^-2^	5.6 ± 0.9	13.1 ± 2.2	5.6 ± 1.3
6	Echinenone isomer	0.3 ± 0.2	0.04 ± 0.03	3.5 ± 2.7 × 10^-3^	1.1 ± 0.6	3.5 ± 2.8	1.1 ± 0.6
7	All-trans-echinenone	0.6 ± 0.4	0.06 ± 0.05	5.3 ± 4.7 × 10^-3^	1.9 ± 0.6	4.2 ± 2.0	1.4 ± 0.4
All	Total carotenoids	28.7 ± 9.6	1.5 ± 1.0	0.3 ± 0.1	na	na	na

Peaks correspond to the spectra shown in **Figure 6F**. The concentration for body fluid samples (HF and PF) is calculated per million cells, including all coelomocyte types. The concentration for the Polian vesicle samples is calculated based on the dry weight (na means not applicable). Results are presented as mean ± SD (n = 4).

### 3.7 HELs are distributed across the different tissues and form red bodies

**Figure 7:**
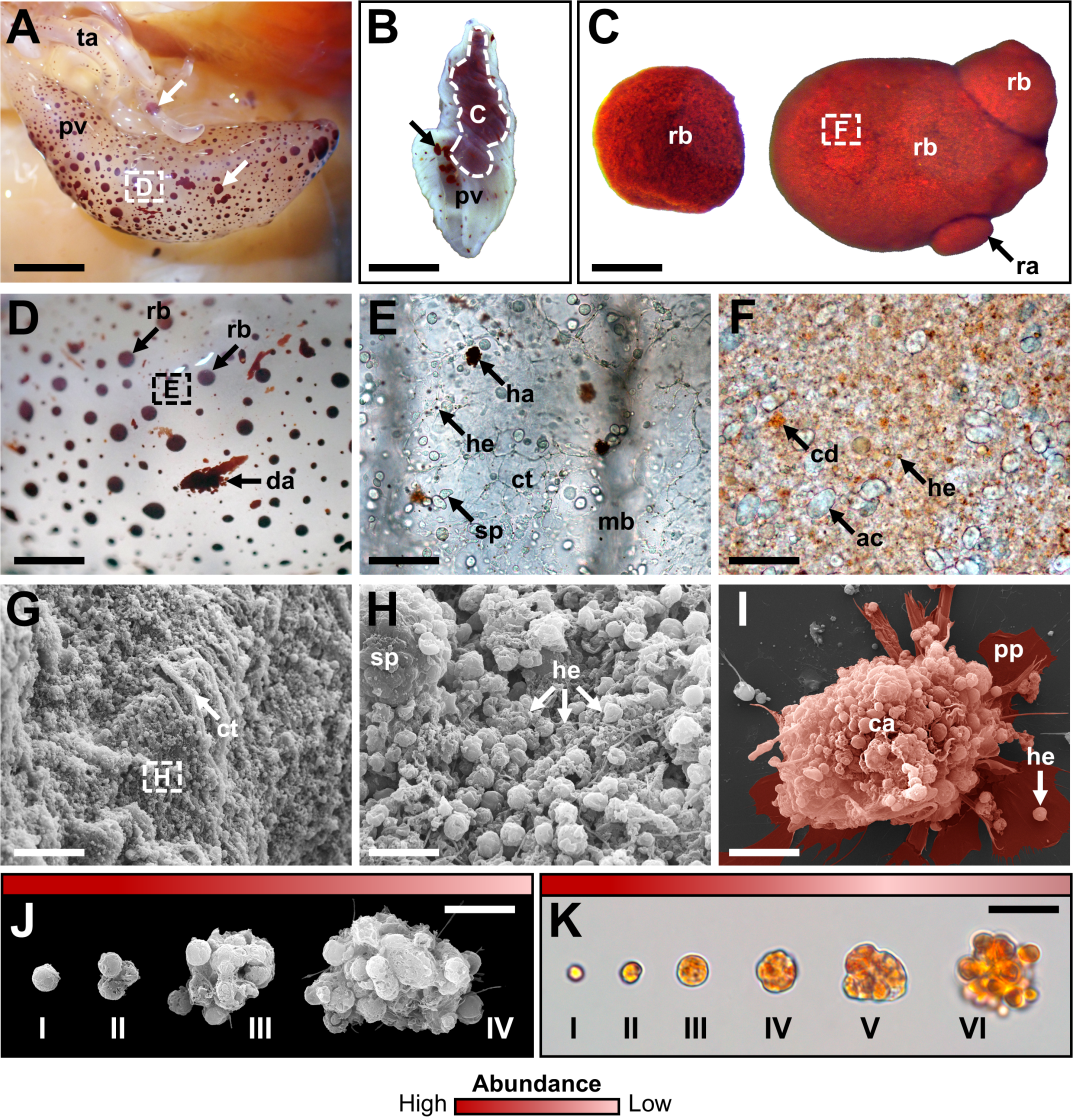
Distribution and morphological diversity of HELs. **(A)** Polian vesicle and ampullae of the buccal tentacles showing marginated aggregates (indicated by white arrows). **(B)** Poured Polian vesicle showing free red aggregates (indicated by black arrow). **(C)** Red aggregates of different shapes are found in the hydrovascular fluid (HF) of the Polian vesicle. **(D)** High magnification of marginated aggregates on the inner surface of the Polian vesicle; some disorganised outer aggregates are also visible. **(E)** Inner surface of the Polian vesicle showing marginated HEL aggregates, HELs, and spherulocytes. **(F)** Cells constituting the red aggregates, including putative apoptotic coelomocytes and HELs; a few conspicuous coloured spots are visible, possibly corresponding to carotenoid deposition. **(G)** SEM view of the inner surface of the Polian vesicle showing a large number of cells. **(H)** Close-up (SEM view) on the inner surface of the Polian vesicle showing numerous marginated HELs and a large spherulocyte. **(I)** Early aggregate stage II observed after incubation of the HF cells for 30 min on a slide; the colour indicates the cell aggregate and its pseudopodia, and many HELs are visible within the aggregate. **(J, K)** Different sizes of HELs and/or HEL aggregates, respectively, in SEM and light microscopy, illustrating the high adhesive properties of these cells as well as a potential fragmentation process of large HELs (their relative abundance is shown by a red scale; darker red means more abundant). Legend: ac, presumed apoptotic cell; ca, coelomocyte aggregate; cd, presumed carotenoid deposition; ct, connective tissue; da, disorganised aggregate; ha, HEL aggregate; he, HEL; pp, pseudopodia; mb, muscular band; pv, Polian vesicle; rb, red body; sp, spherulocyte; ta, tentacle ampulla; dotted contours refer to the subsequent images. Scale bars represent 95 mm in A; 11 mm in B; 340 µm in C; 4.5 mm in D; 75 µm in E; 100 µm in F; 25 µm in G; 5 µm in H; 11 µm in I; 6 µm in J; and 14 µm in K.

As previously demonstrated, HELs are mainly observed in the hydrovascular system. However, within this system, HELs can take different forms and localise to different compartments. This section aims to further describe HELs and their pathways throughout the body of *H. forskali* (**Figure 7**). From a macroscopic to a microscopic point of view, the first evidence of the presence of HELs is large red bodies measuring from 200 to 2000 µm (**Figures 7A-I**). These bodies can be attached to the inner wall of the PV and buccal tentacle ampullae (**Figures 7A, E**) or free in the HF (**Figure 7C**). Round red aggregates appear to detach easily when the PV is shaken or poured (**Figure 7B**). Most of the red bodies were round, but some were irregular (**Figure 7D**). Flattening them under a microscope slice revealed some intact HELs, but the red pigmentation appears to be distributed throughout the aggregate matrix, with some even more pigmented areas forming reddish patches (**Figure 7F**). In addition to the HELs, other cells are visible but have an empty appearance and do not correspond to the types observed in the fresh cell suspension. Moving on to a more microscopic view, the inner membrane of the Polian vesicle shows a large number of cells which appear to be marginated, i.e., attached to the inner membrane (**Figure 7G**). Among these cells, many HELs can be recognised as well as spherule cells (**Figure 7H**). HELs are present in two forms: some are isolated (i.e., individual cells), and others form small aggregates composed solely of HELs. These small HEL aggregates were generally composed of 2 to 50 cells and generally did not exceed 100 µm in diameter. Zooming in further on an individual with a more reddish PV reveals a higher density of HELs forming a “cell bed” on the inner membrane of the PV, although a few spherule cells are still visible. In addition to the red bodies and the HEL aggregates, early coelomocyte aggregates (as in section 3.2) were observed in the HF (**Figure 7I**). Finally, the examination of the cells in the HF enabled us to distinguish different sizes of HELs and HEL aggregates (**Figures 7J, K**). HELs formed highly compact HEL aggregates of different sizes (ranging from single cells—stage I to clusters of several dozens of cells—stage IV), suggesting strong intercellular interactions (**Figure 7J**). In light microscopy, different size groups could also be distinguished: size I measuring 1.7 µm, size II measuring 2.8 µm, size III measuring 5.4 µm, size IV measuring 7 µm, size V measuring 10 µm, and size VI measuring 15 µm (**Figure 7K**). The most abundant sizes were sizes I and II, with size III also frequent; the three sizes accounted for more than 90% of the free HELs. Sizes IV and V showed a granular appearance and were larger. Size VI could be attributed to a HEL aggregate, as in **Figure 7J**, and was also more common than sizes IV and V. However, the fact that it is very similar to size V (**Figure 7K**) suggests that this cellular element might be the result of large HEL fission, releasing size I and size II HELs.

### 3.8 The autofluorescence signature of HELs allows their isolation by FACS

**Figure 8:**
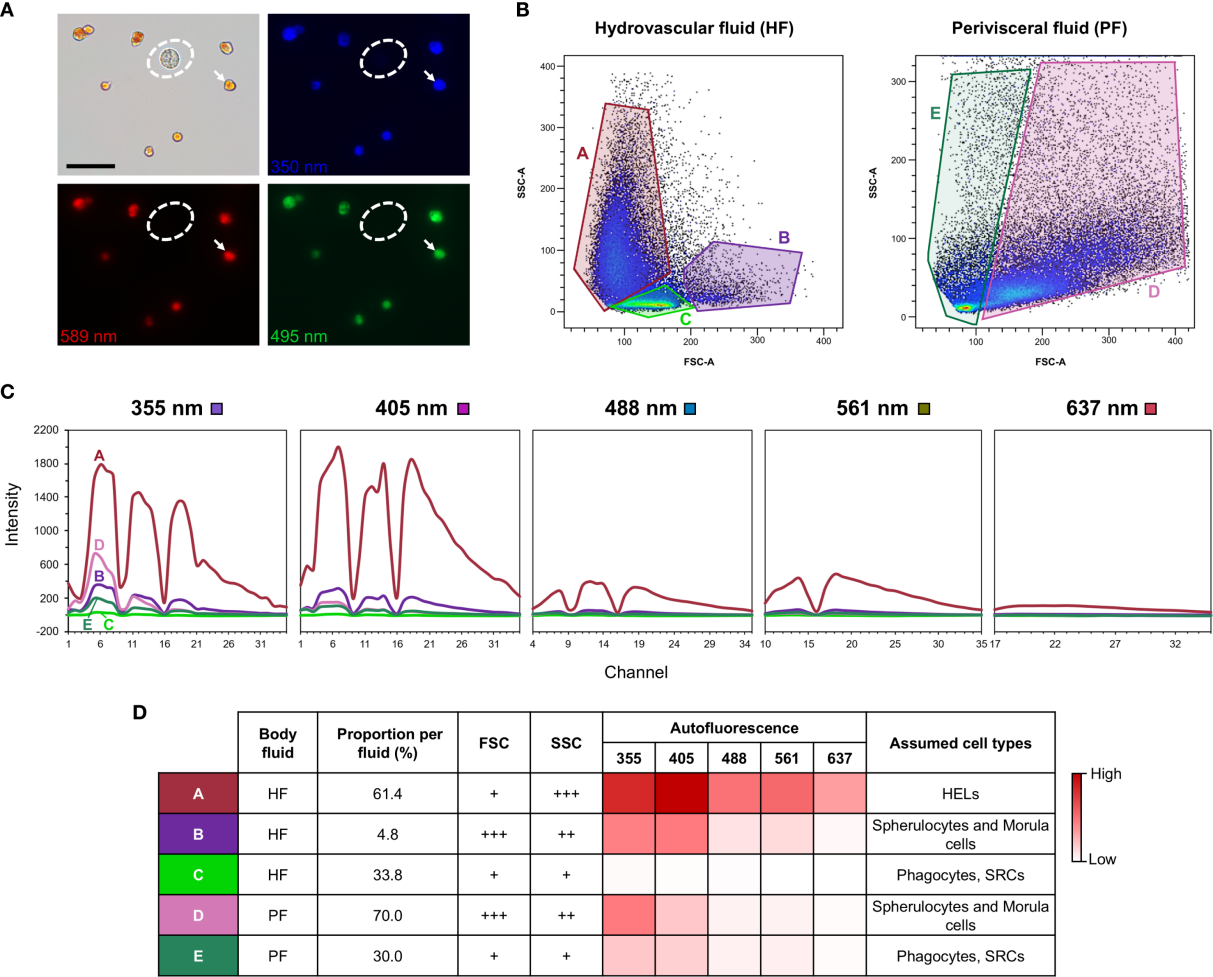
Autofluorescence (AF) of coelomocytes from perivisceral fluid (PF) and hydrovascular fluid (HF) revealed by spectral flow cytometry in *Holothuria forskali*. **(A)** Coelomocytes from the HF observed by fluorescent microscopy at different excitation wavelengths (350, 495, and 589 nm; the dotted ellipse surrounds a spherulocyte, and the white arrow shows a haemocyte-like cell—HELs). **(B)** Flow cytometry profile of HF and PF coelomocytes according to the cell size (forward scatter channel—FSC) and cell granularity (side scatter channel—SSC); the gating strategy is shown in **Supplementary Figure 2**. **(C)** AF spectra overlay for each population obtained by spectral flow cytometry; the graph shows the AF intensity for the different spectra, ranging from dark violet (355 nm) to red (637 nm). Individual AF spectra as well as resulting HF and PF total spectra are found in **Supplementary Figure 8**. **(D)** Characteristics of each population: the corresponding body fluid; the proportion of the population (i.e., the number of events in the population out of the total number of events in the population of the fluid); FSC—size; SSC—granularity (+ means low; ++ means intermediate; and +++ means high); the mean AF intensity in the different channels considered, represented by a colour scale (a darker colour means higher AF); cell types assigned to each population according to their different characteristics.

When observing coelomocytes under a fluorescent microscope, it is possible to note that certain cell types present different AF levels at different excitation wavelengths. HELs, in particular, showed high AF at all the wavelengths tested, including dark violet (350 nm), blue (485 nm), and yellow (589 nm), while at equivalent exposures, the other coelomocyte types were not visible (**Figure 8A**). In order to obtain a more quantitative comparison of AF between the different populations, coelomocytes from both fluids were studied using spectral flow cytometry. With classical size (FSC) and granularity channels (SSC) (gating strategy in **Supplementary Figure 2**), five different populations were defined, namely, populations A, B, and C in the HF and populations E and D in the PF (**Figure 8B**; **Supplementary Figure 8** for full individual spectra). HELs were easily identified as population A by comparing the cell profile between fluids (as they are only present in the HF). In flow cytometry, this population corresponds to small granular cells. The comparison of AF spectra between the five populations (**Figures 8C, D**) confirms that population A (corresponding to HELs) was the most autofluorescent population in all the channels tested and that this AF is higher than that of the other cell types. Furthermore, two other populations, namely, population B of the HF and population D of the PF, showed slight AF in the dark violet (355 nm) and violet (405 nm) channels (**Figure 8D**). They also have a higher granularity which, with their proportion, suggests that these cells might be spherule cells (i.e., spherulocytes and morula cells). The other two populations, populations C of the HF and E of the PF, were intermediate in size with small granularity. They were associated with cell types that do not have granules, notably phagocytes and SRCs.

**Figure 9:**
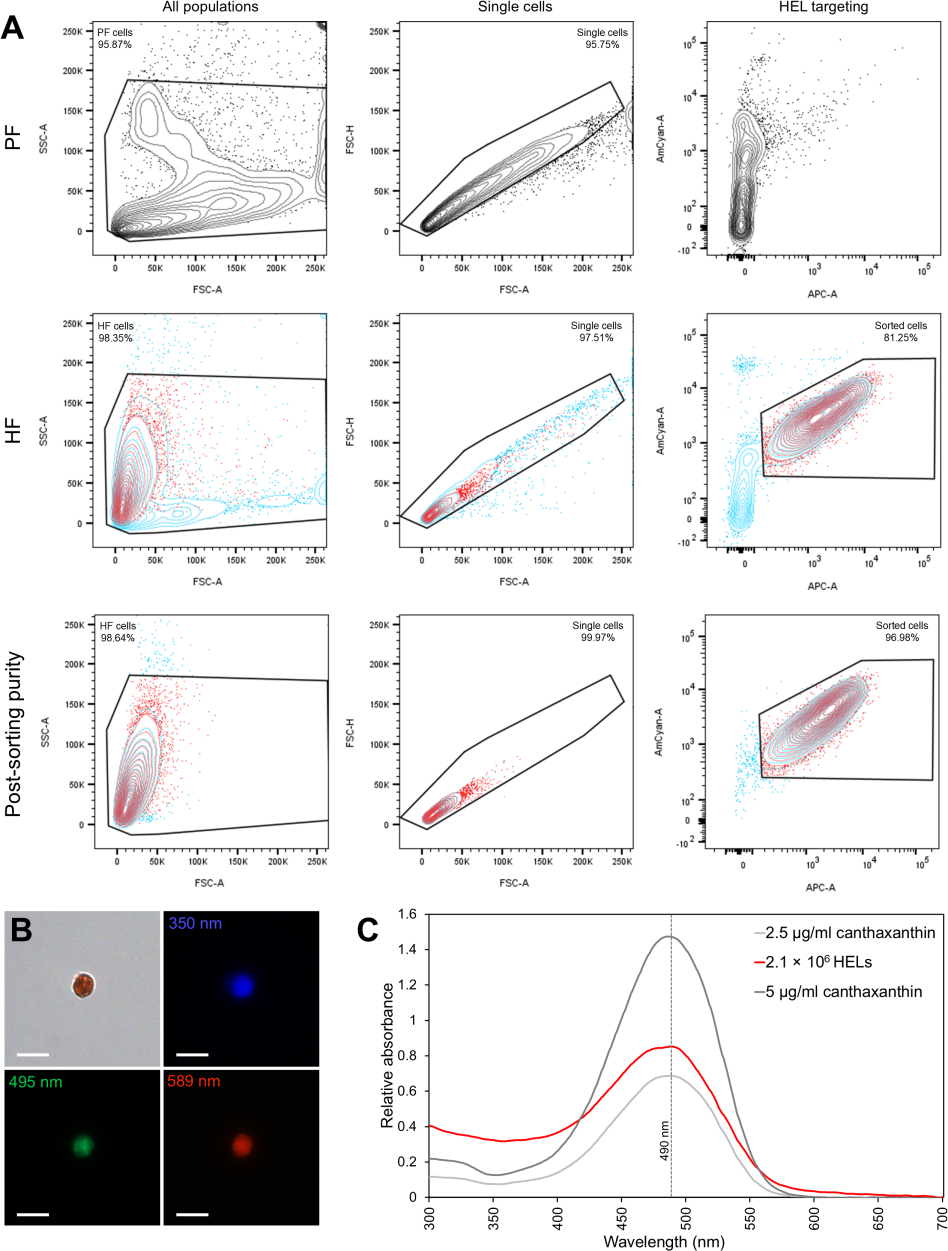
Haemocyte-like cells (HELs) isolated from the hydrovascular fluid of *Holothuria forskali* by fluorescent-activated cell sorting (FACS). **(A)** Sorting strategy based on autofluorescence (AF) of HELs. The HEL population is identifiable by comparing the HF and the PF samples using fluorescent channels (AmCyan and APC). The post-purity test confirms the sorting of a highly enriched HEL population. **(B)** AF of isolated HELs observed by fluorescent microscopy at different excitation wavelengths (λ = 350, 495 and 589 nm). The scale bar represents 10 µm. **(C)** Comparison of the absorbance spectra between the extracted pigment from the purified sample of HELs and the different dilutions of a canthaxanthin standard with known concentrations. The three spectra show a very similar pattern with a peak at 490 nm.

To confirm that HELs are carotenoid-carrying cells, we took advantage of the AF criteria established using spectral flow cytometry to develop a method for isolating HELs by FACS (**Figure 9**). Using the two fluorescent channels APC and Amcyan, it was possible to segregate the HEL population from the less-autofluorescent populations (**Figure 9A**). The fact that this population corresponds to HELs is confirmed when looking at the PF sample with the same channels, which does not show any fluorescent population. HELs were sorted from the HF sample with a HEL proportion of 85.1%. After the sorting, the purity of HELs was assessed by analysing the sorted sample with the same gating strategy, which resulted in a purity of 97.0% (**Figure 9A**). In addition, the HEL purity was assessed by visual check under a microscope, which confirmed that no other cell types were present in the sample and that purified HELs by FACS still showed the same autofluorescent characteristics as freshly collected ones (**Figure 9B**). A total of 2.1 × 10^6^ HELs were sorted to perform a targeted pigment analysis on this sample. Pigment analysis confirms that this sample has a very similar spectrum to canthaxanthin with a peak at 490 nm (**Figure 9C**). By comparing the absorbance obtained with a canthaxanthin standard curve, it was possible to determine that the mass of carotenoid was 2.9 µg for this sample, i.e., 1.4 µg of carotenoid per 10^6^ HELs.

### 3.9 HELs as a driver of differential expression between HF and PF

**Figure 10:**
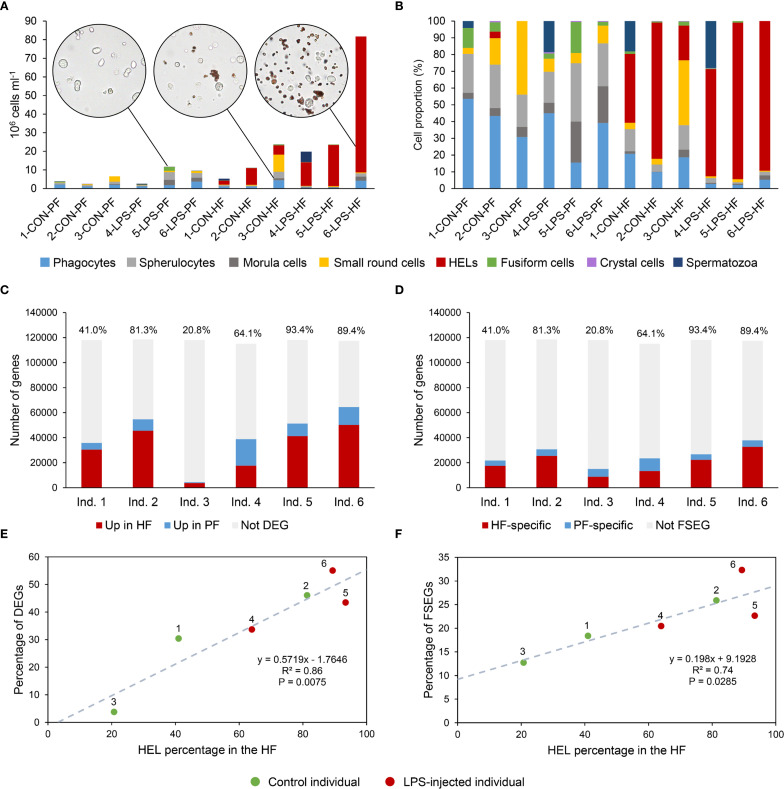
Relation between cell populations and differential gene expression between perivisceral fluid (PF) and hydrovascular fluid (HF) in *Holothuria forskali*. **(A)** Cell population concentration in the different sequenced samples. Pictures of representative unprocessed fluids are included. **(B)** Cell population proportion in the different sequenced samples. **(C)** Number of differentially expressed genes (DEGs) in the comparison between HF and PF for each individual. **(D)** Number of fluid-specific expressed genes (FSEGs; i.e., only expressed in one of the two fluids). The percentage of HELs is indicated above the histograms in C and D, suggesting a correlation with the number of DEGs and FSEGs, respectively. **(E)** and **(F)** Relation between HELs proportion in the HF and proportion of DEGs in E or FSEGs in D (equation of the linear regression; coefficient of determination—R^2^ and p-value of the Pearson's correlation test are included in the graph).

By hypothesising that each cell type has its own gene expression, the presence of HELs in HF and not in PF (or at least in a small proportion) should influence the differential expression between the two fluids. The first evidence of this can be observed in the differential expression analysis between the two fluids; while results of section 3.3 demonstrate that the HEL proportion increased in LPS-injected individuals, the differential expression was more marked between the two fluids when considering LPS-treated individuals. This suggests a link between HEL proportion in the HF and differential gene expression between the two fluids. To further investigate this effect, the cell proportion and concentration were calculated for each sequenced sample (**Figures 10A, B**) and a differential expression analysis between the two fluids for each individual was carried out (**Figure 10C**). As found previously, LPS-injected individuals had a higher proportion of HELs in the HF at 82.3 ± 15.9% versus 47 ± 30.8% in control individuals among these individuals. Regarding differential expression analysis, the number of DEGs was variable between samples and seemed to correlate with the proportion of HELs in the HF (**Figure 10C**). The same analysis was done for genes that were expressed in only one fluid—FSEGs, and it followed the same trend (**Figure 10D**). Based on these observations, a significant relation was obtained between the HEL proportion in the HF and DEG proportion between PF and HF (Pearson’s correlation test: R^2^ = 86%; t = 5.0; df = 4; p = 0.0075; **Figure 10E**), indicating that 86% of the variation in the number of DEGs between the two body fluids is explained by the proportion of HELs in the HF. Regarding the FSEGs, although the linear model had a lower coefficient of determination (R^2^ = 74%), a significant relation was also revealed (Pearson’s correlation test: t = 3.6; df = 4; p = 0.029; **Figure 10F**). Overall, these relations tend to demonstrate that genes overexpressed in HF have a high probability of being overexpressed in HELs.

### 3.10 Proteomics validation of differential transcriptomics and search for candidate marker proteins of HELs

**Figure 11:**
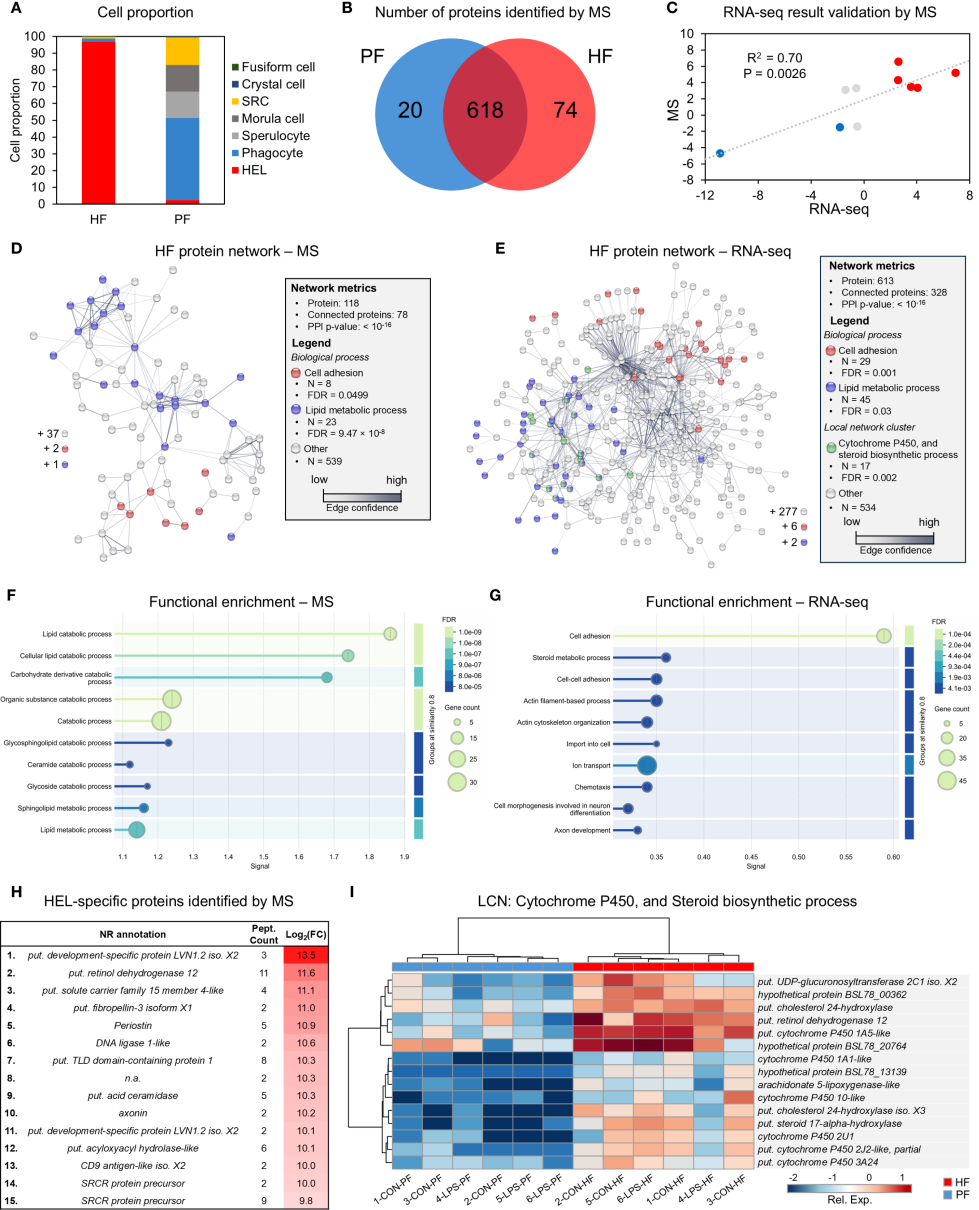
Specific expression of haemocyte-like cells (HELs) and validation of RNA sequencing analyses (RNA-seq) by mass spectrometry (MS). **(A)** Proportion of cell types in hydrovascular fluid (HF) and perivisceral fluid (PF) samples analysed by MS. Note that the HF is constituted of 97% of HELs. **(B)** Venn diagram of proteins identified by MS between PF and HF. The protein list and associated metrics are shown in **Supplementary Table 8**. **(C)** Relationship between the fold changes (FC) in RNA-seq and MS analyses between HF and PF for proteins annotated with a DEG from RNA-seq analysis (PF vs. HF) and having only one gene annotation (blue dots—downregulated in the HF in the two analyses; red dots—upregulated in PF in the two analyses; grey dots—conflicting results). The coefficient of determination—R^2^ and the p-value are indicated on the graph (Pearson's correlation test). Protein annotations are shown in **Supplementary Table 8**. **(D)** and **(E)** String protein network based on proteins with a FC > 5 in MS analysis and proteins (i.e., translated genes) with an FC > 2 in RNA-seq analysis, respectively (the legend includes protein network metrics and functional enrichment analysis, including two biological processes shared between MS and RNA-seq networks, and a local network cluster analysis for RNA-seq analysis; results of the string mapping is shown in **Supplementary Table 9**). **(F)** and **(G)** The 10 most enriched biological processes (based on the gene ontology database) of the protein networks in **(D)** and **(E)**, respectively. **(H)** The 15 most differentially expressed proteins between PF and HF according to MS analysis. For each protein, the annotation, the peptide count, and the Log_2_FC are indicated. **(I)** A heat map of the differential expression between PF and HF of the proteins in the local network cluster “cytochrome P450 and steroid biosynthesis process” from the protein network in E, with their gene annotation (Nr annotation).

To confirm the results obtained by differential transcriptomics and identify HEL-specific proteins, a proteomics comparison between HF and PF coelomocytes from the same individual was carried out by MS. The HF of this individual was highly enriched in HELs, accounting for 97.0% of the cells in this sample (**Figure 11A**). Coelomocyte proportions in the PF fluid were similar to those found previously, with 49% of phagocytes followed by 31.5% of spherule cells (i.e., morula cells and spherulocytes), 16.5% of SRC, and only 2.5% of HELs. A total of 712 proteins could be identified, of which 618 were shared between the two body fluids, 20 were only expressed in the PF, and 74 were only expressed in the HF (**Figure 11B**; the list of proteins is shown in **Supplementary Table 8**). These proteins were annotated with the *de novo* transcriptome obtained from HF and PF coelomocytes (see section 3.4). To confirm the differential expression between the two body fluids, the FC values obtained by MS were compared with those obtained by RNA-seq. For this analysis, only proteins that had only one matching gene in the list of DEGs between the two fluids were selected, resulting in 10 proteins (see **Supplementary Table 8** for the list of proteins and their annotation). A significant relation was found between the two techniques, validating, therefore, the RNA-seq differential expression analysis (**Figure 11C**; R^2^ = 70%; Pearson's correlation test: t = 4.3; df = 8; p = 2.6 × 10^−3^). To further investigate the function of HELs, proteins upregulated in HF were extracted from MS and RNA-seq analyses (i.e., translated from genes) to build functional protein networks against the proteome of *A. japonicus*. For the proteomics analysis, only proteins having an FC >5 were selected, giving a total of 133 proteins. After extracting coding sequences from their annotation and aligning them against the *A. japonicus* proteome, 118 proteins resulted (**Figure 11D**). For the RNA-seq analysis, unigenes with a fold change >2 were selected. The same steps were followed based on translated peptide sequences, which resulted in 613 proteins in the final network (**Figure 11E**; the result of the STRING mapping is shown in **Supplementary Figure 4**). For the two analyses, the network had significant protein interactions (string PPI p-value < 10^−16^). The same analysis was done for upregulated proteins in the PF by RNA-seq and MS, and the resulting networks had a lower number of proteins (108 and 52, respectively) with poorly significant PPI p-values (**Supplementary Figure 9**). Functional analyses using the “gene ontology” database highlight two common biological processes between MS and RNA-seq analyses, namely the “cell adhesion” process (FDR = 0.049 and 0.001, respectively) and the “lipid metabolomic process” (FDR = 9.5 × 10^−8^ and 0.03, respectively). In addition, RNA-seq analysis revealed a local network cluster of 17 proteins corresponding to “cytochrome P450, and steroid biosynthetic process” that was significantly enriched (FDR = 0.002; **Figure 11E**). The 10 most enriched biological processes for MS and RNA-seq are also shown in **Figures 11F, G**, respectively, to further characterise the global function of the protein networks. While MS analysis shows mainly lipid-related biological processes (e.g., “lipid catabolic process” and “cellular lipid catabolic process”), RNA-seq analysis reveals mainly cytoskeleton-related processes (e.g., “cell adhesion”, “actin-filament based process”, and “actin cytoskeleton organisation”). Furthermore, to highlight the most specific proteins in the HF sample, the 15 proteins most differentially expressed in MS analysis were listed (**Figure 11H**). The three most differentially expressed proteins were “putative development-specific protein LVN1.2 isoform X2” (log_2_FC = 13.5), “putative retinol dehydrogenase 12” (log_2_FC = 11.6), and “putative solute carrier family 15 member 4-like” (log_2_FC = 11.1). Regarding their peptide counts, they vary between 2 and 11, with “putative retinol dehydrogenase 12” having the highest, reflecting a more robust annotation for this protein (**Figure 11H**). Finally, the annotation and expression of proteins from the local network cluster “cytochrome P450 and steroid biosynthetic process” were analysed using a heat map visualisation for their interest in the carotenoid metabolism (**Figure 11I**; two proteins were filtered out by a t-test filtering due to excessive variance). As expected, it shows a clear distinction between the two body fluids, with a higher expression in the HF (**Figure 11I**). Regarding the annotation, six proteins are notably annotated as “cytochrome P450”. Moreover, one protein is annotated as “putative retinol dehydrogenase 12”, highlighting similar results between MS and RNA-seq analyses (**Figures 1H, I**).

Parallel to these analyses, a search for genes coding for “globin” and “haemoglobin” was carried out in line with the haemoglobin-based pigmentation hypothesis of HELs. While four genes could be identified as coding for globin based on a Blast search against the transcriptome of PF and HF (**Supplementary Figure 10A**), none were differentially expressed between the two fluids (**Supplementary Figures 10B, C**). Moreover, no protein was annotated with one of these genes in the MS analysis, reflecting a low expression level. These results demonstrate that globins are not particularly expressed in HF and in HELs.

### 3.11 HELs do not produce ROS but express antioxidant enzymes

**Figure 12:**
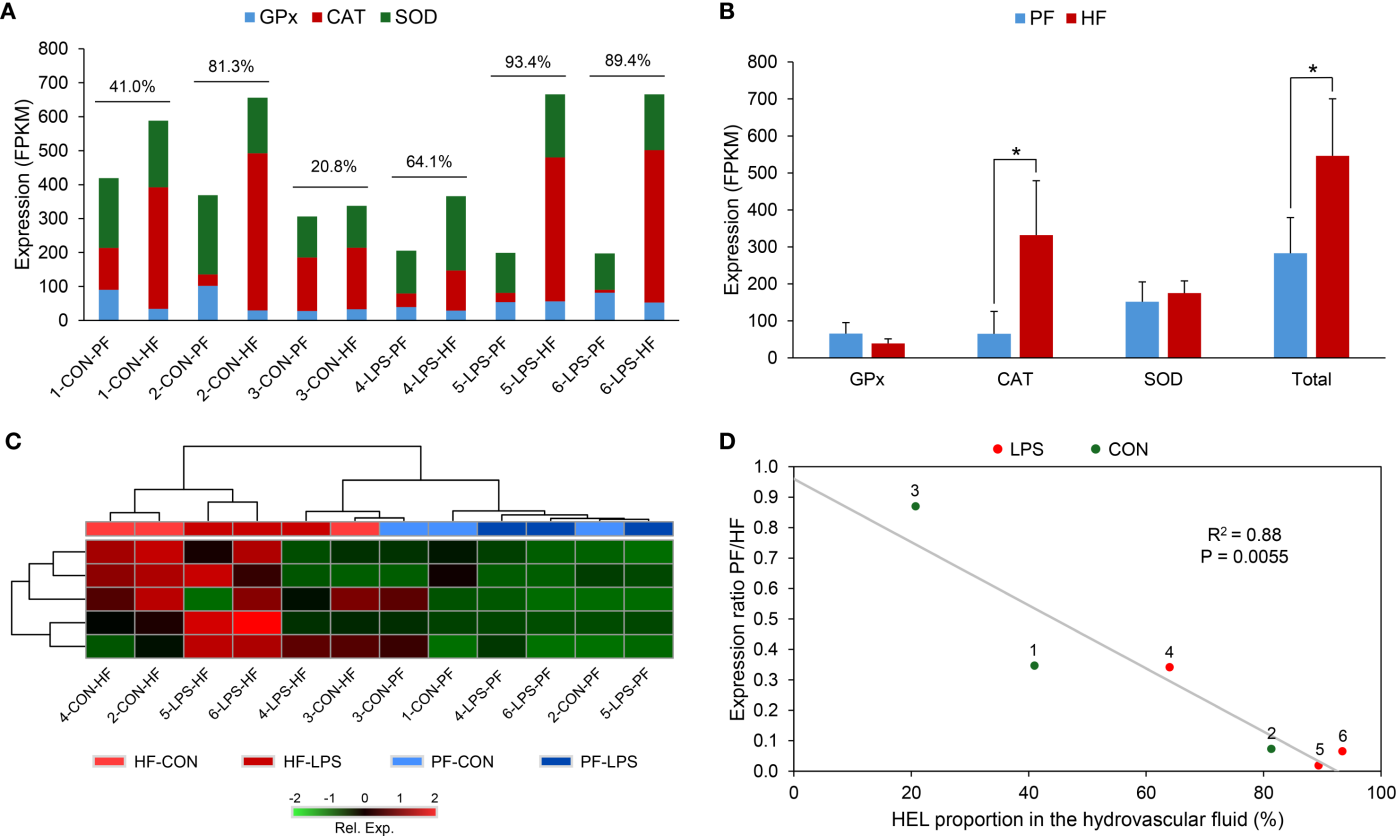
Expression of antioxidant genes in perivisceral fluid (PF) and hydrovascular fluid (HF) of *Holothuria forskali*. **(A)** For each individual, the total expression of the three antioxidant gene families in PF and HF (i.e., the sum of the FPKM values of the genes annotated in the gene family). The proportion of haemocyte-like cells (HELs) is shown above the histograms of each individual, suggesting a correlation between catalase expression and the proportion of HELs. **(B)** Comparison of total antioxidant gene expression between PF and HF, meaning the sum of expression of genes from the three gene families. Error bars represent the SD (n = 6; Wilcoxon signed rank test; * p-value < 0.05). **(C)** Heat map based on the expression of the five catalase genes expressed in coelomocytes. **(D)** Relation of the PF/HF ratio of catalase total expression as a function of the proportion of HELs in the HF (Pearson's correlation test). Legend: CAT, catalase; CON, control individual; GPx, glutathione peroxidase; LPS, LPS-injected individual; Rel. Exp., relative expression; SOD, superoxide dismutase.

In order to characterise the function of the HELs in more detail, the redox balance was studied, assuming that the carotenoids contained in the HELs are antioxidant molecules, representing potentially an essential function of these cells. Firstly, a search was carried out for the expressed genes with an antioxidant function, namely, superoxide dismutase (SOD), catalase (CAT), and glutathione peroxidase (GPx), among the DEGs between the two fluids. This resulted in one gene annotated as CAT being overexpressed in HF (PF versus HF: log_2_FC = 3.43; FDR = 0.011) and one gene annotated as SOD being overexpressed in PF (PF versus HF: log_2_FC = -1.87; FDR = 0.041). However, by examining the entire transcriptome, it was possible to identify 8 genes coding for SOD, 5 coding for CAT, and 20 coding for GPx expressed in the PF or HF. To study the expression of these genes as a functional entity, the total expression of the different genes was calculated (**Figure 12A**) and compared between the two body fluids (**Figure 12B**). This shows that the total expression of these genes is significantly higher in HF (Wilcoxon signed-rank test: W = −21; p = 0.0313), which is mainly due to the total expression of CAT, which is also significantly overexpressed in HF (W = −21; p = 0.031). On the other hand, the total expressions of SOD and GPx were similar between the two body fluids (p > 0.05). Although only one CAT gene showed a significant differential expression between the two fluids, the other four genes encoding CAT appear to follow the same trend (i.e., in each individual, they are more expressed in the HF). Indeed, when examining their expression on a heat map, a relatively good clustering between the two body fluids can be noticed, although the 3-CON-HF sample clusters with 3-CON-PF (**Figure 12C**). Nonetheless, it is known from cell counting that this HF sample is the one containing the lowest proportion of HELs (**Figure 12A**), suggesting a relationship between the proportion of HELs and CAT expression. To verify this, the total expression ratio PF/HF of CAT for each individual was correlated with the proportion of HELs in the HF. This results in a significant correlation (R^2^ = 88%; Pearson’s correlation test: t = −5.4; df = 4; p = 0.0055), meaning that the higher the proportion of HEL in the HF, the lower the PF/HF ratio of CAT expression (**Figure 12D**). This tends to show that HELs overexpress CAT genes compared with other cell types, but not other antioxidant genes such as SOD and GPx.

**Figure 13:**
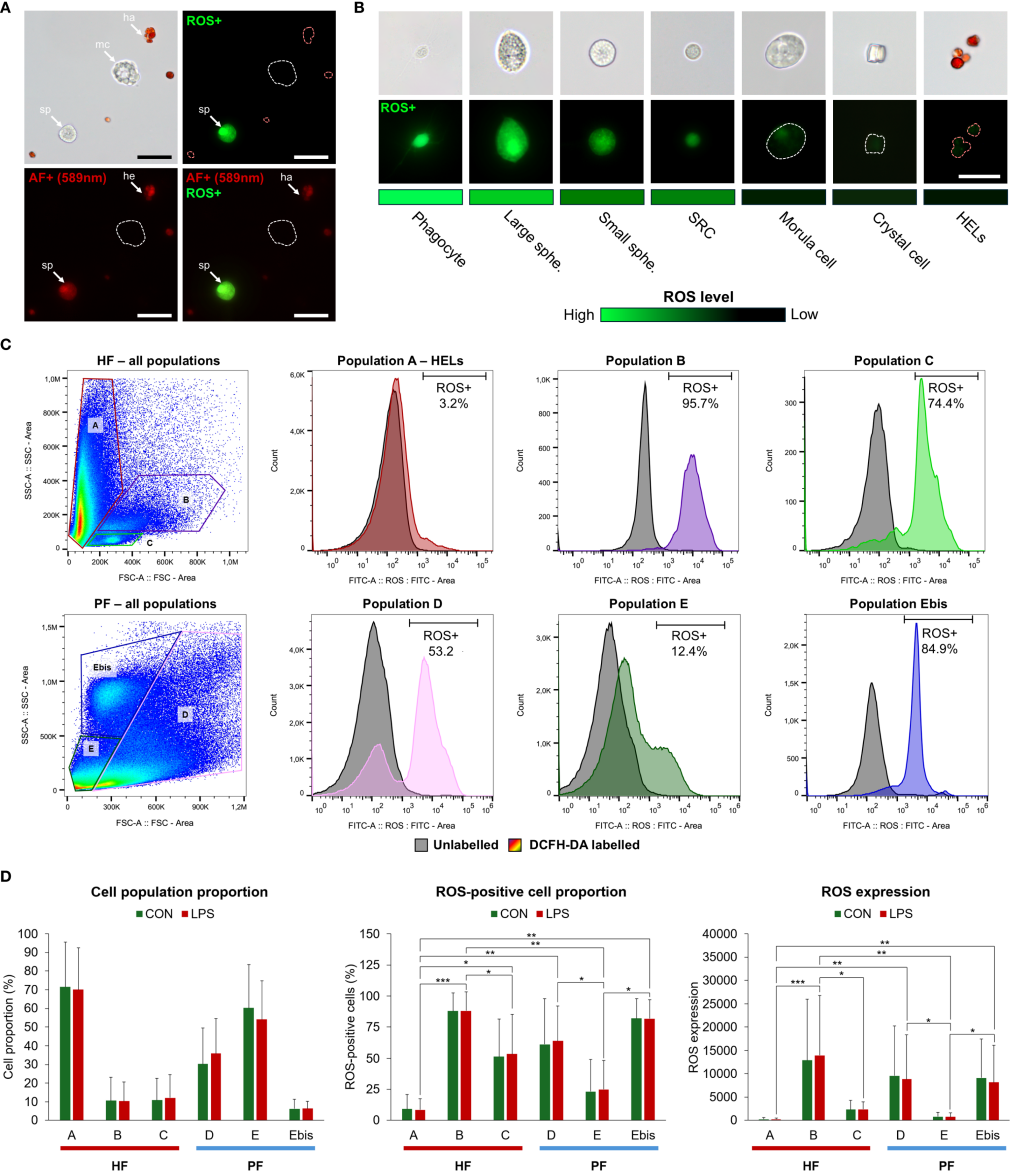
Study of the production of reactive oxygen species (ROS) by coelomocytes from hydrovascular (HF) and perivisceral fluids (PF) in *Holothuria forskali* using DCFH-DA labelling. **(A)** DCFH-DA labelling of HF coelomocytes under fluorescent microscopy: while haemocyte-like cells (HELs) are autofluorescent at 589 nm, the autofluorescence (AF) of HELs is no longer visible with the excitation wavelength of DCFH-DA (495 nm) when there are ROS-positive cells around (he, HEL; mc, morula cell; sp, spherulocyte; a negative control is shown in **Supplementary Figure 11**). The scale bars represent 20 µm. **(B)** DCFH-DA labelling of different types of coelomocytes photographed with the same intensity; the lighter green, the higher the level of ROS (the dotted lines in A and B indicate where the localisation of the cell is when they are not visible). Scale bars represent from left to right: 20, 14, 12, 10, 15, 16, and 10 µm. **(C)** ROS production quantified between different coelomocyte populations using spectral flow cytometry: the graph on the left shows the population gating of coelomocytes from HF and PF; histograms on the right show, for each population, the distribution of cells as a function of fluorescence intensity (black—no DCFH-DA labelling; coloured—same population with a DCFH-DA labelling; the proportion of ROS-positive cells is indicated on the graph based on the defined threshold). The gating strategy is shown in **Supplementary Figure 12**. **(D)** Comparison of the proportion of ROS-positive cells and ROS expression between the different populations and between lipopolysaccharide-exposed (LPS) and control (CON) samples based on the quantification method in **(C)** The graph on the left shows the proportion of each population; the middle and right graphs show the proportion of ROS-positive cells and ROS expression (based on the median fluorescence intensity), respectively. Results are formulated as means with error bars representing the SD (n = 4 in LPS and n = 3 in CON; Friedman test; p-value: * < 0.05; ** < 0.01; *** < 0.001).

In parallel with the analysis of antioxidant gene expression, ROS production between different coelomocyte types was studied using the DCFH-DA, a ROS marker. First, ROS-positive cells were visualised using a fluorescence microscope (**Figures 13A, B**). While the HELs showed high AF at 495 nm, this was no longer perceptible in the presence of other ROS-positive cells, being highly fluorescent (**Figure 13A**). Note that DCFH-DA labelling was confirmed by examining the same samples without DCFH-DA (**Supplementary Figure 11**). By examining the different types of cells individually with the same fluorescence intensity, it was also possible to show that ROS production varies considerably between coelomocyte populations: Phagocytes and large spherulocytes are strongly ROS-positive; small spherulocytes and SRCs are moderately ROS-positive; and morula cells, crystal cells, and HELs are not ROS-positive (**Figure 13B**). To confirm these results quantitatively, ROS production was studied by spectral flow cytometry using PF and HF cells with and without LPS exposures (see gating strategy in the PF and the HF in **Supplementary Figure 12**, respectively). In terms of populations, the results showed a similar profile to that obtained previously (see section 3.8), except that the E population of the HF was divided into two subgroups, namely, the E and Ebis populations, because in this analysis, we noticed a higher density of highly granular cells (i.e., higher SSC for the Ebis population; **Figure 13C**). It is known, from the comparison between the two fluids and the sorting experiment, that population A corresponds to HELs (see section 3.8). By comparing the proportion of ROS between the different populations, it is clear that HELs show a lower proportion of positive ROS compared with the other populations (**Figure 13C**). A statistical comparison was then conducted to compare the proportion of ROS-positive cells and ROS production (based on median fluorescence) between the different populations exposed or not exposed to LPS (**Figure 13D**). Firstly, no clear difference could be found between the two conditions, neither in terms of the proportion of ROS-positive cells nor in terms of ROS production. Secondly, in the comparison of cell populations, significant differences were observed in the proportion of ROS-positive cells and ROS production (Friedman χ^2^ = 18.6; df = 5; p = 0.0023 and Friedman χ^2^ = 16.7; df = 5; p = 0.0051, respectively). For both measures, HELs have the lowest value with 8.4 ± 8.9% of ROS-positive cells and a median fluorescence of 246.3 ± 237.2 (n = 4). These are significantly different from all other populations, except for population E in the PF, corresponding to small cells that also had a relatively low level of ROS-positive cells and ROS production (25.1 ± 23.6% and 715.5 ± 888.2, respectively). In comparison, the most ROS-positive populations, namely, population B in the HF and population D in the PF, have a proportion of ROS-positive cells of 87.9 ± 15.2% and 61.1 ± 27.9% and a ROS production of 13,936.2 ± 12,813.5 and 8,828.1 ± 9,525.0 (n = 4), respectively. In addition, cell viability was investigated between the different conditions using PI staining. However, no difference was revealed, neither between the conditions nor between the cell populations (**Supplementary Figure 13**), with an overall low mortality proportion ranging from 1.2 ± 0.5% for population C to 13.6 ± 11.3% for population Ebis.

## 4 Discussion

### 4.1 Coelomocyte types are not equally distributed between coelomic fluids

The wide diversity of coelomocyte morphotypes in sea cucumbers suggests a complex immune system in which each cell type fulfils distinct functions in immunity. However, very little information exists on these respective functions. With their distinctive coelomic compartment containing the HF and PF, sea cucumbers offer a promising model for studying the distinctive function of these body fluids, potentially reflected by their respective cellular composition. In *H. forskali*, 11 main morphotypes could be distinguished under light microscopy (including five subtypes and two uncertain types), with seven main cell types generally observed and included in cell counts. These results confirm previous studies on other sea cucumber species (e.g., 51 for *A. japonicus*; 18 for *H. scabra*; 37 for review). They also corroborate the presence of a large number of small reddish cells—HELs—notably similar to the recent description of haemocytes in the order Holothuriida (22). Our primary results demonstrated that coelomocyte types are not equally distributed between PF and HF, with a conspicuous difference in the almost exclusive presence of HELs in the HF, where they appear to be the predominant type. In contrast, spherulocytes and crystal cells were more represented in the PF than in the HF. While differences in the concentration of cell types between the two fluids have rarely been investigated (e.g., in 52), certain common patterns are shared between species: crystal cells were similarly more prevalent in the PF of *H. scabra* (18), and the so-called haemocytes were found to be restricted to the HF in many species including *Allothyone mexicana*, *Bohadschia argus*, *Cucumaria frondosa*, *Eupentacta quinquesemita*, *H. atra*, *H. scabra*, *H. forskali*, and *Sclerodactyla briareus* (22, 23, 53–55). These differences in the cellular composition of the two fluids, mainly represented by the remarkably high concentration of HELs in the HF, suggest divergent functions associated with their respective cellular composition.

### 4.2 Coelomocytes of the two body fluids are immunocompetent

The activity and behaviour of the cells were followed during immune response activation to study the function associated with the different cell types. Although fluid extraction does not truly represent immunological stress, we noticed that when no anticoagulant solution was added, the cells initiated amoeboid-like movements and formed aggregates. In this case, the immune activation could be triggered by the recognition of damage-associated molecular patterns released during dissection, such as intracellular proteins or nuclear DNA in the extracellular environment (56). As some cellular behaviours were characteristic of some cell types, such an approach can be informative about the function of different cell types. According to our observations, the process begins with the activation of phagocytes that adhere to the slide and extend their pseudopodia to recruit other types of cells. This recruitment is thought to be induced by the release of humoral factors such as cytokines and agglutinins, which we have identified as being widely expressed in coelomocytes in our transcriptomics analysis. These could activate the mobility of other cells and attract them by chemotaxis. Spherule cells, in particular, showed a significant degree of mobility through a characteristic amoeboid movement based on the formation of spherical protrusions at the apex of the cell, followed by an undulation, giving them a figure-eight shape. This particular mobility is reminiscent of the bleb-driven mobility, a particular amoeboid movement based on an increase in the hydrostatic pressure inside the cell and distinct from pseudopod-based mobility (57). This ability may confer on the cells a “swimming ability” that could be useful for a fast circulating cell recruitment to the site of infection. In addition, some spherule cells could carry out sudden cell lysis when they encountered an aggregate, releasing numerous granules. Previous reports suggested that spherulocytes or morula cells could perform similar cell lysis to release granules and amorphous material based on morphological analysis during the formation of brown bodies following the injection of foreign material (58, 59). The released granules could also explain the observation of minute corpuscles (i.e., cell fragments) in several studies (37). Furthermore, our results revealed that early aggregates, by becoming increasingly compact and merging, can move autonomously on the slide. Similar observations were reported by Taguchi et al. (60), who proposed that this “crawling ability” could be useful for the aggregate to migrate to the sites of injury. In HF, we were able to confirm that HELs also participate in this process. Although they are not mobile, they appear to be highly interactive when they encounter another cell or a cell aggregate. The aggregation process is known to be an essential immune mechanism in echinoderms, leading to the encapsulation of foreign matter and pathogens (23, 60, 61). This process is also thought to be responsible for the formation of coloured bodies in the different echinoderm classes (41). In *H. forskali*, large red bodies were conspicuous in the Polian vesicle and buccal tentacle ampullae, similarly to the description of these red bodies in *C. frondosa* (23). Overall, these results confirm that aggregation is an important immune mechanism in sea cucumbers and provide new information on the first stages of this phenomenon, involving the HELs in the HF.

Immunostimulation with LPS indicated that only the concentration of HELs in the HF was significantly increased one day after the injections, which suggests that this type of cell has a specific function in the immune response. Changes in the coelomocyte population have already been studied in *H. glaberrima* and *H. scabra* following injections of various pathogen-associated molecular patterns (PAMPs), including LPS (18, 62). Although these species show different patterns in the modifications of the cell populations, both displayed a clear response to PAMPs. Furthermore, the timing of the immune response is important to take into account and could have prevented the detection of changes in other cell populations in our study. In *C. frondosa*, for example, the concentration of coelomocytes was monitored at different time points after various stresses, including the presence of a predator or an injured conspecific (63). In this study, the peaks of phagocytes and morula cells preceded the peak of haemocytes and began to decrease 3 h after the stress. Therefore, we cannot exclude that an increase in the concentration of other cells occurred earlier and that only a peak in haemocytes was visible at that time, and further temporal analyses are required to confirm any changes in the concentration of other cell types at shorter time intervals. Moreover, the high responsiveness of coelomocytes to many non-pathogenic stressors (64), including handling (23), makes it difficult to obtain a real negative control. Therefore, stress related to the detention of individuals might have influenced the concentration of some cell type responding to non-specific stress (i.e., not necessarily pathogenic). In any case, our result showed that HELs increased after one day, suggesting, in combination with the study of Hamel et al. (63), that HELs act relatively late in the immune response.

The immune response to LPS injection was also assessed at the level of gene expression between the two body fluids. Both showed a clear transcriptional response, with many physiological pathways related to the immune system that were differentially expressed and shared between them. These include “NOD-like receptor signalling pathways” or “Toll and Imd signalling pathways”, which are important pathogen recognition pathways (14, 65, 66). Other pathways involved in immune mechanism regulation include apoptosis, necroptosis, the “IL-17 signalling pathway”, or the “NF-kappa B signalling pathway”. These pathways are consistent with previous studies investigating the gene expression of coelomocytes following exposure to PAMPs in holothuroids, notably in *H. leucospilota* and *H. scabra*, in which a high expression of cytokines and NOD-like receptor genes was reported (18, 19). In contrast to these pathways, some were not enriched in the same way between the two body fluids, including the “PI3K-Akt signalling pathway” and “RIG-I-like receptor signalling pathway”, which were respectively more differentially expressed in the HF and the PF during the immune response. The “PI3K-Akt signalling pathway” is notably involved in cell proliferation and epithelial–mesenchymal transition (67). During an immune response, it was proposed that haemocytes, initially marginated to the inner wall of hydrovascular tissues, could detach to enter the cell suspension (23). This is consistent with our observation of a large number of HELs on the inner membrane of the Polian vesicle and their increase in concentration following the immunological stress that only occurred in the HF. Differential expression of this pathway could, therefore, reflect the demargination process during the immune response (23, 67). The “RIG-I-like receptor signalling pathway” is a pathogen recognition receptor pathway that has an important function in innate immunity and can notably cooperate with Toll-like receptor signalling (68). Overall, gene expression analyses following the injection of LPS demonstrated that coelomocytes of both fluids are highly responsive cells expressing a large diversity of immune genes, including numerous homologues of vertebrate immune genes (2, 13).

### 4.3 Unexpected characteristics of HELs

Reddish-pigmented cells in holothuroids have historically been considered and designated as “haemocytes” because of their haemoglobin content (6, 44). Consequently, it has been suggested that their function is to oxygenate tissues by participating in the transport of oxygen (39, 69). This hypothesis on the function of haemocytes has been widely accepted for over a century and has been posited as a universal characteristic of haemocytes in sea cucumber species. To study the function of the HELs in more detail, we decided to take advantage of the significant enrichment of these cells in the HF to compare them with the coelomocytes of the PF. Surprisingly, we discovered that the HF coelomocytes contained an enormous quantity of carotenoids that correlated with the number of HELs in the samples analysed. Furthermore, by performing the same analysis on FACS-purified HELs, we unequivocally demonstrated that HELs were the pigment-carrying cells. All the results support that HEL pigmentation is due to carotenoids. They also exclude the involvement of red water-soluble pigments such as haemoglobin or phycoerythrin, which could be excluded based on their solvent affinity (70). Other lipophilic pigments, including chlorophylls, could be ruled out based on their distinct spectral properties (71). In order to characterise this newly described pigmented cell type in more detail, various analyses were carried out.

First of all, our results showed that the HELs were located mostly in the HF, where they were generally observed in large red bodies or formed small aggregates of approximately 10 cells that were generally marginated at the inner membrane of the Polian vesicle. While most HELs are small, we observed some that were larger and granular in appearance. Furthermore, given that some HELs were minute (less than 2 µm), we hypothesised that some HELs could instead result from the release of small anucleate corpuscles by larger ones. This hypothesis is consistent with previous observations of haemocytes forming cytoplasmic protuberances that detached as small spheroids before the entire cell underwent cytolysis (5, 72). Hetzel (5) also mentioned that some of these vesicles contained refractive yellow granules, suggesting a possible role in the release of pigments or metabolites. This process of cytolysis is also reminiscent of platelet production in vertebrates, which results from the cellular lysis of megakaryocytes (73). Although further research is needed to confirm this fragmentation ability, this potential mechanism of HEL fragmentation might enhance the organism’s ability to promptly respond to physiological needs and facilitate the distribution of these corpuscles across tissues or within cellular aggregates to carry out various biological processes.

Another characteristic of the HELs was their strong autofluorescence (AF). This AF was attributed to the high concentration of carotenoids inside the cell, as it has been shown that astaxanthin-producing green algae also exhibit a strong AF (74) that was notably used in flow cytometry analyses (75). In the present study, we also took advantage of this AF using spectral flow cytometry to determine the best strategy for isolating these cells by FACS. FACS has also been used to sort the red spherule cells in sea urchins according to their AF, which has made it possible to better characterise the pigment contained in these cells (21, 76). While sea urchin red spherule cell pigments are rather naphthoquinones (77), the strategy for isolating pigmented cells based on AF has now also proven to be effective in sea cucumbers with carotenoids, which offers good prospects for the use of this technique in other echinoderm species in the future.

### 4.4 Carotenoid diversity and acquisition in sea cucumbers

HPLC analyses have shown that the carotenoids contained in HELs are mainly composed of canthaxanthin (~55%) and astaxanthin (~45%) and, to a lower proportion, echinenone (<5%) (**Figure 1**). Astaxanthin and canthaxanthin are keto-carotenoids found in a wide variety of aquatic organisms, including shrimps, crabs, crayfish, and fish (78–81). In sea cucumbers, a wide variety of carotenoids have been identified, mainly in the integument, gonads, or other viscera (e.g., 82). These pigments include astaxanthin, canthaxanthin, and echinenone, in accordance with our results, as well as β-carotene, phoenicoxanthin, fucoxanthin, and cucumariaxanthin, which were not detected in *H. forskali* (82–84). Recently, David et al. (45) studied the diversity of carotenoids in the gonad of *H. forskali*, showing a very similar composition in the same types of carotenoids but with a much lower concentration compared with the Polian vesicle sample (which contained a large amount of HELs). This suggests that the carotenoid profile within a species is relatively constant between the different tissues. In contrast, different proportions of carotenoids are generally observed between species. For example, Matsuno and Tsushima (85) identified three new carotenoids, namely, cucumariaxanthines A, B, and C, which were specific to the order Dendrochirotida, suggesting a possible taxonomic specificity in their distribution. This difference in carotenoid composition could thus explain why certain coloured coelomocytes, described as haemocytes, may have a different colouration. For example, it has been shown that the haemocytes in *H. scabra* are brownish (18, 23), which was initially attributed to lower oxygen concentrations in the body fluid of this tropical species, resulting in a reduced form of haemoglobin (22). Following our results, it could reasonably be suggested that this colour is due instead to the presence of different carotenoid types. Further research is underway to identify the nature of the HEL pigmentation in various species of sea cucumbers belonging to different taxonomic groups and with different ecologies to better determine which factors shape this pigmentation.

Our results indicate that the carotenoid content of HELs varies between 1.4 and 30 µg per 10^6^ cells. This range is not far from that reported for the green algae *Haematococcus pluvialis*, which can reach higher values, such as 80 µg per 10^6^ cells, but are larger cells (86). Although these values cannot be compared with those of metazoan tissues, generally measured as a percentage of the mass, a comparison of the results obtained with the Polian vesicle samples reveals a much higher concentration than those found in salmon or krill, known to have a high carotenoid content (87). Metazoans, with a few exceptions, cannot synthesise their carotenoids *de novo* and must acquire them through their diet (80). The transformation and storage of such a quantity of carotenoids can be costly for the metabolism and require a certain amount of time to accumulate them (87). Consequently, the possibility of storing these carotenoids in specialised cells for long-term use could constitute a significant advantage. In echinoderms, carotenoids were notably found in different species and organs of sea stars (Asteroidea) (e.g., 88, 89), where they are supposed to help cope with environmental stresses (89). However, it is still unclear if they are acquired through their diet (88). In many holothuroid species, including *H. forskali*, a high concentration of carotenoids was found in gonads, which was attributed to a protective effect for the gametes and a potential source of energy for the embryos (45, 82). Nonetheless, the mechanism of such an accumulation of carotenoids in the gonads remains unclear. David et al. (45) hypothesised that the acquisition of carotenoids could occur during gonadal maturation through diet. However, they could not find any difference in carotenoid content throughout the year, which raised questions about the source and allocation of these carotenoids. They concluded that they could originate from the reabsorption of carotenoids in the spent gonadal tubules (after spawning). During our experiments, large quantities of HELs and coelomocyte aggregates were observed in the dehiscent gonadal tubules (unpublished results), which suggests that these circulating cells could play a central role in the transport and recycling of carotenoids. Furthermore, the study of the composition of the diet in the intestine of *H. forskali* reveals mainly the presence of fucoxanthin, which would come from brown algae (90). This type of carotenoid is not a direct precursor of keto-carotenoids such as canthaxanthin and astaxanthin (80). However, a recent study testing different rearing methods for *H. forskali* juveniles in coculture with oysters and abalones revealed the presence of β-carotene and, to a lesser proportion, canthaxanthin (29). These pigments are precursors of astaxanthin, which means that under certain conditions (80), *H. forskali* could directly accumulate these precursors. Although it remains unclear whether *H. forskali* can acquire carotenoids throughout its life, it can be reasonably assumed that the HELs could regulate the use of carotenoids by acting as long-term storage inside the hydrovascular system. It is also important to note that, in contrast to other carotenoid-rich organs such as gonads or digestive tract, the Polian vesicle(s), and the hydrovascular system in general, are not expelled during self-evisceration (91), allowing them to keep a carotenoid stock even during the regeneration process. This stock of carotenoids could serve as an energy reserve during periods when resources are limited. Finally, haemocytes have been found in association with various tissues in *C. frondosa*, including muscle bands and haemal vessels (23, 92), which supports their role in delivering carotenoids to different tissues.

### 4.5 Differential expression between the two body fluids provides insight into the function of HELs

Our results showed that gene expression between the two body fluids tends to be more divergent under immunostimulation conditions than under immunoquiescent conditions. This coincides with the increase in the proportion of HELs that only occurs in the HF of immunostimulated individuals. In addition, many pathways were not shared in the comparison of PF versus HF between immunostimulated and immunoquiescent condition analyses. It is interesting to note that a large proportion of these pathways were not related to the immune system, but rather to the endocrine and digestive systems, with a higher overall number of upregulated genes in the HF, suggesting that this fluid is also involved in the transport of nutrients and regulation of physiological processes through hormonal control. This endocrine-related activity could also reflect an involvement in the immune-neuro-endocrine (INE) axis, a conserved system found in both vertebrates and invertebrates. The INE axis integrates immune, neuroendocrine, and metabolic functions through bidirectional signalling, whereby immune cells can both produce and respond to neuroendocrine mediators, thereby coordinating defence mechanisms with metabolic and homeostatic processes (93). In our study, the high proportion of endocrine-related transcripts in the HF could therefore play its dual role in immunity and systemic physiological regulation.

By counting the proportion of coelomocyte types in the sequenced samples, it was possible to correlate differential gene expression with the proportion of HELs in the HF, meaning that a large part of these functions can be attributed to HELs. This approach, combining cell counting and comparative transcriptomics, proved to be very informative in terms of cell function. Moreover, thanks to this, we were able to select samples where the concentration of spermatozoa was reasonable in comparison with the proportion of coelomocytes, which would not have been possible without checking the cellular composition of our samples before extracting the RNA. Such gamete contamination might explain why some differences can be observed in the expression of immune genes in coelomocytes between males and females in echinoderms (e.g., this study, **Supplementary Figure 6**; 94). For these reasons, we strongly encourage determining the cellular proportion of the samples when considering omics analyses on echinoderm coelomocytes.

On the basis of the genes and proteins that were upregulated in HF, we were able to identify two broad biological functions characteristic of HELs, namely, “cell adhesion” and “lipid metabolic processes”. The processes in cell adhesion encompass proteins involved in the interaction with the extracellular matrix and interaction with other cells (95, 96). This is consistent with the characteristics of HELs, which are often observed in small aggregates or marginated at the wall of the Polian vesicle (23, 95). This process involves proteins such as integrins, cadherins, and laminins, which were overexpressed in the HF. As for the “lipid metabolic processes”, they can reasonably be attributed to the metabolism of carotenoids, which are lipidic molecules synthesised from tetraterpene precursors (97). We have been notably able to identify candidate proteins involved in carotenoid metabolism, particularly in the pathway “cytochrome P450, and steroid biosynthetic process”. In this metabolism, six proteins have been annotated as cytochromes P450 and are overexpressed in the HF. These proteins are highly conserved enzymes with a function in the biosynthesis and detoxification (98). However, it has been shown that they can have a bifunctional activity by also playing the role of carotenoid-ketolase, which transforms “conventional” carotenoids such as β-carotene into ketocarotenoids such as canthaxanthin and astaxanthin. This property was first demonstrated in birds, in which the expression of a gene belonging to the cytochrome P450 family, namely, CYP2J19, was identified as responsible for the red-beaked phenotype (99). Later, a similar carotenoid-ketolase function of cytochrome P450 genes was reported in several lineages, including spider mites, shrimps, and scallops (100–102) but, to our knowledge, not in echinoderms. It has been suggested that the acquisition of this bifunctional activity of cytochrome P450 would result from the evolutionary convergence of these enzymes under different types of selective pressure (102). Interestingly, disruption in the expression of cytochromes P450 can lead to yellow-brown phenotypes, as illustrated by spider mites (102). Given that some species of sea cucumber have brown HELs, such as *H. scabra*, it could be hypothesised that these species have lost or never acquired the bifunctional cytochrome P450 enzyme. However, further research is needed to determine whether this difference in pigmentation is due to genetic variation or different ecological parameters, such as different food resources. In addition to cytochrome P450, we have been able to identify genes coding for “retinol dehydrogenase 12” and “UDP-glucuronosyltransferase”, which are also thought to play a role in carotenoid metabolism. Retinol is a derivative of vitamin A, synthesised from β-carotene, a precursor of canthaxanthin and astaxanthin (80, 103). UDP-glucuronosyltransferase is an enzyme also involved in detoxification, in particular by catalysing the transfer of glucuronic acid to small lipophilic molecules (104). It has recently been shown that it is differentially expressed in the integument between different colour morphotypes of *A. japonicus*, suggesting that this gene may also have a role in regulating integument pigmentation through the carotenoid metabolism (105). More broadly, cytochrome P450 metabolisms have been reported in several studies investigating colour phenotypes in sea cucumbers without making a direct link with the carotenoid-ketolase activity highlighted in other lineages (e.g., 105, 106). These results suggest, therefore, that this metabolic pathway plays a key role in sea cucumber pigment processing.

In addition to these enzymes, we listed the 15 most enriched proteins in HELs obtained by MS. Among them, two were annotated as “putative developmental specific protein LVN1.2 isoform X2”. Surprisingly, this protein has been reported as a marker of endodermal tissues in sea urchin embryos (107) and has also been overexpressed in the intestine following heat stress in sea cucumbers (108). Although further research is underway to identify the origin of HELs, this expression suggests that these HELs may have a distinct origin from other types of coelomocytes (109). Overall, this dataset could help to identify this cell type in other studies and other species, particularly with regard to single-cell RNA datasets in sea cucumbers.

### 4.6 Function of HELs in the immune response regulation through the redox balance

Beyond their function in pigmentation, it has been shown that carotenoids play several roles in photoprotection, reproduction, and immunity (89, 110–112). These are notably enabled by their distinctive structural composition, incorporating a hydroxyl group and a carboxyl group, which give them high antioxidant properties (113). Among these molecules, astaxanthin has been identified as the carotenoid with the most potent antioxidant properties (114, 115). In addition, carotenoids have also been identified as antimicrobial molecules (116, 117). Therefore, HELs, with their high concentration of carotenoids and astaxanthin in particular, could be considered antioxidant and antimicrobial immune cells.

In the same vein, we have demonstrated that HELs produce fewer ROS than other types of coelomocytes. ROS are highly reactive compounds that can be produced during the immune response to neutralise pathogens in a process called respiratory burst (118). However, an excessive concentration of ROS can also be harmful to the host’s tissues, hence the importance of regulating the redox balance during the immune response (111). In addition to their high carotenoid concentration, we have shown that HELs also overexpress catalase, an enzyme that reduces hydrogen peroxide into water and oxygen (111). However, surprisingly, the superoxide dismutase, an enzyme that reduces superoxide into hydrogen peroxide (118), is not overexpressed in HELs. We believe that carotenoids could play this role instead of superoxide dismutase, rendering the overexpression of this enzyme unnecessary. Such competitive activity between carotenoids and antioxidant enzymes has been reported in several invertebrates (119–121), particularly in *Gammarus pulex*, in which the uptake of carotenoids decreased the concentration of ROS and the expression of superoxide dismutase but increased the expression of catalase (119).

In contrast to HELs, certain populations of coelomocytes, including phagocytes and spherulocytes, were strongly positive for ROS. This level of ROS was not higher in the cells exposed to LPS. This could be explained by the harvesting of the fluids and the short-term culture, which may have led to ROS production in the control samples as well, thereby masking any signal of the immunostimulation. Beyond their immune function, ROS have also been described as key mediators of regeneration processes in metazoans (49). Interestingly, it has been shown that coelomocytes are recruited to the site of injury during wound healing or intestinal regeneration (122, 123). Lacouth et al. (124) even reported that among coelomocytes, phagocytes and spherulocytes were the first cell types to be recruited to the site of the lesion. Based on our results, these cell types could constitute a first line of ROS-producing cells aimed at activating regeneration processes at the lesion site. Other types of cells producing a lower quantity of ROS, such as morula cells and HELs, could ultimately be recruited to balance this effect. Overall, it can be hypothesised that the high carotenoid content of sea cucumbers could be an adaptation to their extraordinary regenerative capacities, balancing the need for ROS that could be regulated in the tissues by coelomocytes.

**Figure 14:**
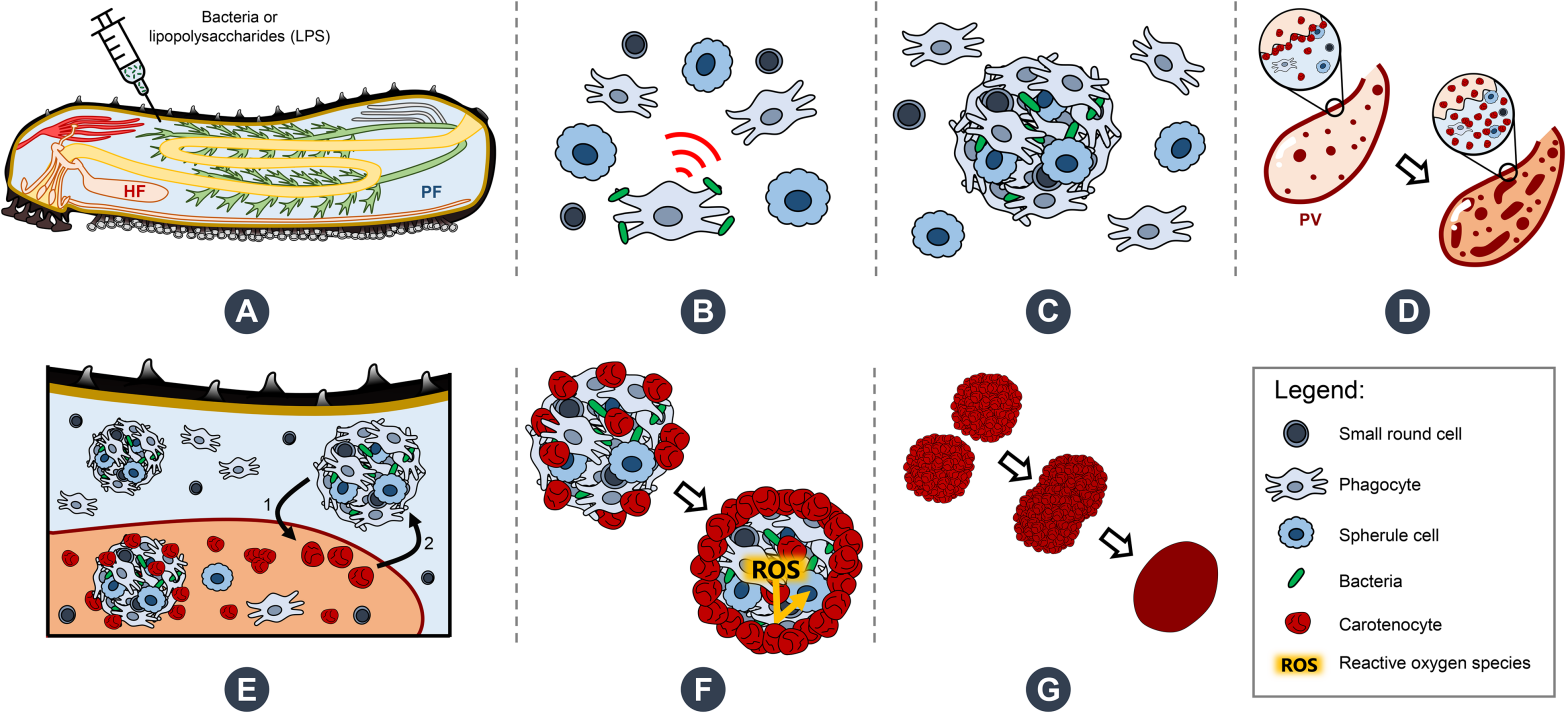
Proposed model illustrating the presumed function of haemocyte-like cells (HELs)—renamed carotenocytes according to our results—in the immune response of *Holothuria forskali* and potentially other sea cucumber species. **(A)** The organism is immunostimulated either by bacterial exposure or lipopolysaccharide injections. **(B)** The immune response is initiated by phagocytes, which start the aggregation process in perivisceral and hydrovascular fluids (PF and HF, respectively). **(C)** Formation of early aggregates occurs through the recruitment of other coelomocytes, leading to the neutralisation of pathogens. **(D)** Releasing of marginated HELs, with potential production and fragmentation of HELs in the HF. **(E)** HELs encounter the aggregates via two possible pathways: either HELs migrate toward the site of infection, or the aggregates migrate toward the hydrovascular system after the pathogen entrapment. **(F)** Aggregates are covered by a layer of HELs, which form an antioxidant shell to mitigate the production of reactive oxygen species (ROS) within the aggregate. **(G)** The red aggregates gather and progressively become more compact to form large red bodies that are stored in the Polian vesicle (PV) for possible recycling of carotenoids and/or eventual excretion.

Given that haemocytes participate in the formation of large red bodies, previous studies have suggested that the haemoglobin contained in these cells could release ROS in the aggregate to neutralise pathogens (23). Although we could confirm the involvement of HELs in the formation of red bodies in *H. forskali*, our results tend to demonstrate the opposite. We have therefore proposed a new model explaining the putative function of HELs, consisting of an antioxidant immune regulator. During a bacterial infection or an injection of LPS, phagocytes, and spherulocytes begin to aggregate, producing ROS within the newly formed aggregate to neutralise the pathogenic agent (**Figures 14A-C**). In a second phase, demargination and potential production of HELs occur in the tissues of the hydrovascular system, with possible fragmentation of the larger HELs to release small spheroids and promote their distribution (**Figure 14D**). Two possibilities are then proposed: these cells could be recruited at the site of the infection or, more unlikely, the aggregates could migrate in the hydrovascular system (**Figure 14E**). Thanks to their high adhesive properties, HELs could form a coating around the early aggregate which, given their high antioxidant properties, would constitute an antioxidant shell. In this way, HELs could restrict ROS production to the aggregate by carotenoids and catalase expression, preventing reactive compounds from damaging the host’s tissues (**Figure 14F**). The resulting coloured aggregates would finally merge to form large coloured bodies (i.e., larger and more compact aggregates) that might be excreted from the body through the cloaca as proposed by Caulier et al. (23) (**Figure 14G**). Although the antioxidant properties of HELs are supported by their gene expression and high carotenoid content, further research is, however, needed to confirm this model *in vivo* and elucidate their potential function in the antioxidant shell formation.

### 4.7 HELs are sea cucumber pigmented coelomocytes—carotenocytes

Finally, with all the evidence that HELs do not contain haemoglobin but carotenoids, we have proposed to rename these cells “carotenocytes”. Although further research is needed to assess the distribution of these cells in sea cucumbers, we believe that a large proportion of the haemocytes that have been described over the past century do not contain haemoglobin but carotenoids. However, we do not totally exclude the potential presence of true haemoglobin-containing cells in sea cucumbers, particularly in certain burrowing species living in low-oxygen environments (e.g., *Paracaudina chiliensis*; 39). The existence of carotenoid-carrying cells has previously been documented in other marine animals, including fish and crustaceans, in which they constitute erythrophores (i.e., carotenoid chromatophores) serving the external colouration (125), but no example of circulating carotenoid-carrying cells in a metazoan was found in the literature. Moreover, Xing et al. (126) recently discovered different pigmented cells in sea cucumbers, namely, the quinocytes and melanocytes in the integument of *A. japonicus*, but to our knowledge, our study is the first to describe pigmented coelomocytes in sea cucumbers (by not considering respiratory pigments). This discovery, therefore, opens up a new paradigm on pigmented coelomocytes in echinoderms, which were previously thought to be only present in sea urchins and sea stars (7, 8, 127) and suspected in brittle stars (128). Furthermore, it is interesting to note that, although sea cucumber carotenocytes and sea urchin red spherule cells contain different pigments, they appear to share certain functions, including in the antimicrobial response and the ability to scavenge ROS (129; **Figure 14**). It could, therefore, be hypothesised that sea cucumbers and sea urchins have independently acquired similar functional cell types through similar immune selection pressure but by two different molecular systems. While further research is underway to determine the significance of the distribution of carotenocytes in sea cucumbers and confirm their function in immunity, carotenoid storage, and the regeneration process, we believe that such cell types are broadly distributed in sea cucumbers and that they play a central role in the sea cucumber homeostasis.

## Data Availability

The datasets presented in this study can be found in online repositories. The names of the repository/repositories and accession number(s) can be found below: https://www.ncbi.nlm.nih.gov/, PRJNA1259574 https://doi.org/10.6084/m9.figshare.c.7839038.

## Supplementary material

Supplementary Video 1 Time-lapse imaging of a spherule cell performing cell lysis upon contact with an early aggregate.

Supplementary Video 2 Stage I early aggregate formation.

Supplementary Video 3 Stage II early aggregate formation.

Supplementary Video 4 Stage III early aggregate formation.

Supplementary Video 5 Formation of early aggregates in the hydrovascular fluid (HF) with the participation of haemocyte-like cells.

Supplementary Table 1 Statistical analysis – cell concentration and proportion difference between hydrovascular fluid (HF) and perivisceral fluid (PF).

Supplementary Table 2 Statistical analysis – cell proportion and concentration after a lipopolysaccharide immunostimulation.

Supplementary Table 3 Metrics of the *de novo* RNA-sequencing (RNA-seq) in *Holothuria forskali*.

Supplementary Table 4 List of differentially expressed genes (DEGs) between control (CON) and lipopolysaccharide-injected LPS individuals.

Supplementary Table 5 KEGG pathway enrichment analysis of the differential expression analysis between control (CON) and lipopolysaccharide-injected (LPS) individuals.

Supplementary Table 6 List of differentially expressed genes (DEGs) between perivisceral fluid (PF) and hydrovascular fluid (HF).

Supplementary Table 7 KEGG pathway enrichment analysis of the differential expression analysis between perivisceral fluid (PF) and hydrovascular fluid (HF).

Supplementary Table 8 Proteins identified by mass spectrometry (MS) and validation of the RNA-sequencing (RNA-seq) differential expression analysis between the two fluids.

Supplementary Table 9 Results of the STRING Mapping on the proteome of *Apostichopus japonicus*.

Supplementary Figure 1 Identification of pigment based on commercial standards.

Supplementary Figure 2 Spectral flow cytometry gating strategy.

Supplementary Figure 3 Spherule cell-specific behaviours and mobility.

Supplementary Figure 4 Automated cell analysis using the Particle Analysis tool in ImageJ software.

Supplementary Figure 5 Transcriptome assembly and general annotation in *Holothuria forskali*.

Supplementary Figure 6 Differential expression analysis of coelomocytes between males and females in *Holothuria forskali*.

Supplementary Figure 7 Linear relationship between astaxanthin and canthaxanthin concentrations.

Supplementary Figure 8 Autofluorescence (AF) spectra of coelomocytes revealed by spectral flow cytometry.

Supplementary Figure 9 String protein networks based on genes overexpressed in the perivisceral fluid (PF).

Supplementary Figure 10 Globin expression in coelomocytes of perivisceral fluid (PF) and hydrovascular fluid (HF).

Supplementary Figure 11 Validation of DCFH-DA labelling for reactive oxygen species (ROS) detection.

Supplementary Figure 12 Flow cytometry gating strategy of reactive oxygen species (ROS) production analysis.

Supplementary Figure 13 Estimation of cell mortality using propidium iodide (PI) labelling and flow cytometry.
