# Supplementary material for: Carotenoid-based immune response in sea cucumbers relies on newly identified coelomocytes—the carotenocytes
Source: Front Immunol. 2025 Nov 6;16:1668167. doi: 10.3389/fimmu.2025.1668167 (PMC12631484; doi:10.3389/fimmu.2025.1668167)
Supplement: Supplementary Table 1 — Statistical analysis – cell concentration and proportion difference between hydrovascular fluid (HF) and perivisceral fluid (PF). [file Table1.pdf]

Tables S1. Statistical analysis results between the hydrovascular fluid (HF) and perivisceral fluid (PF), in specimens of normal condition (no injection). Results are formulated as mean ± SD (n = 6), and p-values (P) show significant differences between the two fluids (Wilcoxon signed rank test; significant differences are in bold; n.a. means not applicable).

	Concentration (cells ml^{-1})				Proportion (%)			
**Coelomocyte types**	**HF**	**PF**	**P**	**W**	**HF**	**PF**	**P**	**W**
**Phagocyte**	$9.28 \pm 6.18 \times 10^{5}$	$1.04 \pm 0.45 \times 10^{6}$	1	-1	10.52 ± 11.33	30.98 ± 10.07	**0.031**	**-21**
**Small spherulocyte**	$3.27 \pm 1.44 \times 10^{5}$	$1.19 \pm 0.41 \times 10^{6}$	**0.035**	**-21**	2.91 ± 1.45	36.04 ± 5.90	**0.031**	**-21**
**Large spherulocyte**	$1.05 \pm 1.28 \times 10^{5}$	$1.41 \pm 1.16 \times 10^{5}$	0.27	-7	1.07 ± 1.12	5.05 ± 3.14	**0.031**	**-21**
**Small round cell**	$5.78 \pm 4.16 \times 10^{5}$	$7.37 \pm 1.61 \times 10^{5}$	0.31	-11	4.49 ± 2.69	23.44 ± 7.36	**0.031**	**-21**
**Haemocyte-like cell**	$1.20 \pm 0.96 \times 10^{7}$	$2.33 \pm 2.34 \times 10^{4}$	**0.031**	**21**	80.62 ± 5.14	0.75 ± 0.71	0.031	21
**Fusiform cell**	$5.67 \pm 5.99 \times 10^{4}$	$1 \pm 1.37 \times 10^{5}$	0.78	-3	0.38 ± 0.35	3.09 ± 3.75	**0.19**	**-11**
**Crystal cell**	$3.33 \pm 8.16 \times 10^{3}$	$2.0 \pm 0.0 \times 10^{4}$	**0.037**	**-15**	0.01 ± 0.03	0.64 ± 0.15	**0.031**	**-21**
**Total (all types)**	$1.40 \pm 0.99 \times 10^{7}$	$3.28 \pm 0.83 \times 10^{6}$	**0.031**	**21**	n.a.	n.a.	n.a.	n.a.
