# Supplementary material for: Carotenoid-based immune response in sea cucumbers relies on newly identified coelomocytes—the carotenocytes
Source: Front Immunol. 2025 Nov 6;16:1668167. doi: 10.3389/fimmu.2025.1668167 (PMC12631484; doi:10.3389/fimmu.2025.1668167)
Supplement: Supplementary Table 2 — Statistical analysis – cell proportion and concentration after a lipopolysaccharide immunostimulation. [file Table2.pdf]

Table S2. Statistical analysis results of concentration and proportion of coelomocyte populations between control and LPS-injected individuals (Mann-Whitney U test; significant differences are in bold).

	Perivisceral fluid (PF)				Hydrovascular fluid (HF)			
**Cell types**	**Control**	**LPS**	**P**	**U**	**Control**	**LPS**	**P**	**U**
**Concentration**								
**Phagocyte**	$1.6 \pm 4.4 \times 10^{6}$	$3.7 \pm 3.8 \times 10^{6}$	0.7308	18	$1.7 \pm 1.3 \times 10^{6}$	$1.8 \pm 1.2 \times 10^{6}$	0.7206	18
**Small spherulocyte**	$1.1 \pm 0.4 \times 10^{6}$	$2.6 \pm 2.2 \times 10^{6}$	0.4452	15	$1 \pm 1.2 \times 10^{6}$	$8.9 \pm 6.2 \times 10^{5}$	1	20.5
**Large spherulocyte**	$2.5 \pm 1.3 \times 10^{5}$	$1 \pm 0.1 \times 10^{6}$	0.2234	12	$2.9 \pm 4 \times 10^{5}$	$8.3 \pm 9.2 \times 10^{5}$	0.1	9
**Small round cell**	$9.5 \pm 11.6 \times 10^{5}$	$1.1 \pm 1.1 \times 10^{6}$	0.7206	18	$1.8 \pm 3.6 \times 10^{6}$	$1 \pm 1.1 \times 10^{6}$	0.3518	14
**Haemocyte**	$1.7 \pm 4 \times 10^{4}$	$2.3 \pm 4.3 \times 10^{5}$	0.4314	16	$3.2 \pm 3.2 \times 10^{6}$	$3.1 \pm 2.1 \times 10^{7}$	**0.0012**	**0**
**Fusiform cell**	$2.4 \pm 2.8 \times 10^{5}$	$5.2 \pm 7.9 \times 10^{5}$	0.6678	17.5	$2.2 \pm 2.2 \times 10^{5}$	$1.8 \pm 2.3 \times 10^{5}$	0.828	19
**Crystal cell**	$3.3 \pm 6.2 \times 10^{4}$	$4.9 \pm 6 \times 10^{4}$	0.2328	12.5	$1.7 \pm 2.3 \times 10^{4}$	$2 \pm 2.6 \times 10^{4}$	1	20.5
**Total**	$4.2 \pm 1.8 \times 10^{6}$	$8.9 \pm 7.6 \times 10^{6}$	0.7308	18	$8.2 \pm 8.1 \times 10^{6}$	$3.5 \pm 2.3 \times 10^{7}$	**0.0082**	**3**
**Proportion**								
**Phagocyte**	42.1 ± 14 %	41.3 ± 10.7 %	0.8301	19	25.6 ± 11.6 %	5.7 ± 3.4 %	**0.0053**	1
**Small spherulocyte**	28 ± 9.7 %	27.9 ± 9.3 %	0.9452	20	13.3 ± 6.1 %	2.8 ± 1.8 %	**0.0023**	1
**Large spherulocyte**	11 ± 8.8 %	5.6 ± 1.4 %	0.2949	13	3.6 ± 2.1 %	2.3 ± 2.7 %	0.3525	14
**Small round cell**	12.9 ± 8.5 %	18.2 ± 14.5 %	0.6282	17	12.8 ± 13 %	2.9 ± 2.3 %	**0.014**	4
**Haemocyte**	0.2 ± 0.3 %	0.6 ± 1.6 %	0.5414	17	41 ± 25.1 %	85.3 ± 6.6 %	**0.0047**	2
**Fusiform cell**	4.9 ± 6.5 %	5.3 ± 5.2 %	0.7748	18.5	3.4 ± 3.9 %	0.5 ± 0.5 %	**0.0123**	3
**Crystal cell**	0.5 ± 0.3 %	0.6 ± 0.9 %	0.6621	17.5	0.1 ± 0.2 %	0 ± 0.1 %	0.5855	17
