# Supplementary material for: Carotenoid-based immune response in sea cucumbers relies on newly identified coelomocytes—the carotenocytes
Source: Front Immunol. 2025 Nov 6;16:1668167. doi: 10.3389/fimmu.2025.1668167 (PMC12631484; doi:10.3389/fimmu.2025.1668167)
Supplement: Supplementary Table 3 — Metrics of the de novo RNA-sequencing (RNA-seq) in Holothuria forskali. [file Table3.pdf]

Table S3. Metrics of the *de novo* RNA-sequencing in *Holothuria forskali*. For each individual transcriptome and the merged transcriptome, the table provides: the sex of the individuals, metrics on raw data (reads metrics) and assembled data (assembly metrics). Read metrics include the total number of raw reads, the total number of clean reads, the total number of clean bases, the percentage of bases whose quality is greater than 20 in clean reads (Q20), and the percentage of clean reads. Assembly metrics include: the total number of unigenes; the total length of the transcriptome; the mean length of unigenes; the N50; and the ratio of GC bases. Legend: ♂ – male; ♀ – female; na – not applicable; nd – non-determined; CON – control individual; HF – hydrovascular fluid sample; LPS – lipopolysaccharide individual; PF – perivisceral fluid sample.

Samples		Read metrics					Assembly metrics				
**Name**	**Sexe**	**Raw reads (M)**	**Clean reads (M)**	**Clean bases (Gb)**	**Q20 (%)**	**Clean reads (%)**	**Total number**	**Total length (M)**	**Mean length**	**N50**	**GC (%)**
**1-CON-PF**	♂	233.89	221.38	22.14	99.0	94.7	42,190	48.85	1,157	2,054	38.4
**1-CON-HF**	♂	236.37	221.93	22.19	99.0	93.9	59,097	77.01	1,303	2,490	39.1
**2-CON-PF**	♀	241.35	221.49	22.15	99.1	91.8	33,934	33.40	984	1,500	38.0
**2-CON-HF**	♀	243.84	221.00	22.10	99.0	90.6	57,175	70.75	1,237	2,301	38.9
**3-CON-PF**	♀	236.37	221.78	22.18	99.1	93.8	56,407	81.03	1,436	2,740	39.7
**3-CON-HF**	♀	233.89	220.49	22.05	99.0	94.3	55,941	82.70	1,478	2,817	39.7
**4-LPS-PF**	♂	233.89	221.74	22.17	99.0	94.8	42,789	47.42	1,108	1,965	38.5
**4-LPS-HF**	♂	241.35	220.62	22.06	98.9	91.4	46,473	41.56	894	1,447	38.1
**5-LPS-PF**	♀	241.35	221.50	22.15	98.9	91.8	37,473	39.18	1,045	1,773	38.2
**5-LPS-HF**	♀	236.37	220.75	22.08	98.8	93.4	57,538	75.27	1,308	2,490	39.2
**6-LPS-PF**	♀	241.35	221.87	22.19	98.8	91.9	30,861	27.73	898	1,269	38.1
**6-LPS-HF**	♀	236.37	222.51	22.25	98.8	94.1	55,386	77.09	1,391	2,629	39.4
**Stone canal**	nd	241.35	220.28	22.03	99.0	91.3	58,800	71.97	1,223	2,267	38.8
**Podia**	nd	233.89	220.25	22.02	99.1	94.2	73,673	95.07	1,290	2,425	39.4
**Total**	na	3331.63	3097.59	309.76	99.0	93.0	167,199	337.36	2,017	3,807	38.9
