# Supplementary material for: Carotenoid-based immune response in sea cucumbers relies on newly identified coelomocytes—the carotenocytes
Source: Front Immunol. 2025 Nov 6;16:1668167. doi: 10.3389/fimmu.2025.1668167 (PMC12631484; doi:10.3389/fimmu.2025.1668167)
Supplement: Supplementary Figure 2 — Spectral flow cytometry gating strategy. [file Image2.pdf]

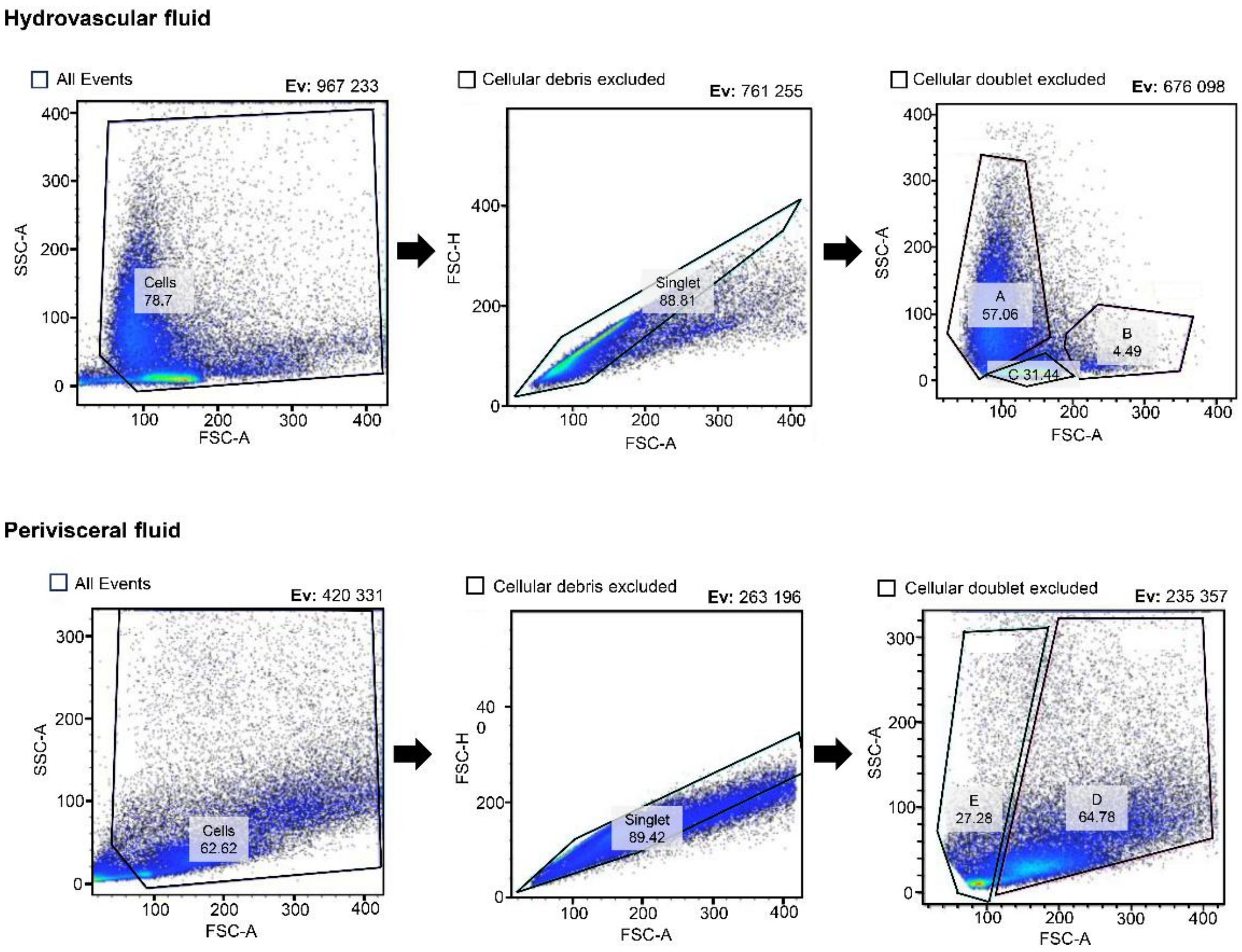

Sup. Fig. 2. Spectral flow cytometry gating strategy of coelomocytes in the hydrovascular (A) and perivisceral (B) fluids *of Holothuria forskali*. The percentages indicated correspond to the percentage of cells (cell events) selected. The size and granularity parameters (FSC-A and SSC-A) were utilised to eliminate cellular debris, which typically exhibits minimal levels of these parameters. Subsequently, the parameters of size (FSC-A) and height (FSC-H) were utilised to eliminate cell doublets, which possess an increased surface area. Finally, the selection of different populations was based on size and granularity parameters (FSC-A and SSC-A). Legend: Ev – number of cell events.
