# Supplementary material for: Carotenoid-based immune response in sea cucumbers relies on newly identified coelomocytes—the carotenocytes
Source: Front Immunol. 2025 Nov 6;16:1668167. doi: 10.3389/fimmu.2025.1668167 (PMC12631484; doi:10.3389/fimmu.2025.1668167)
Supplement: Supplementary Figure 3 — Spherule cell-specific behaviours and mobility. [file Image3.pdf]

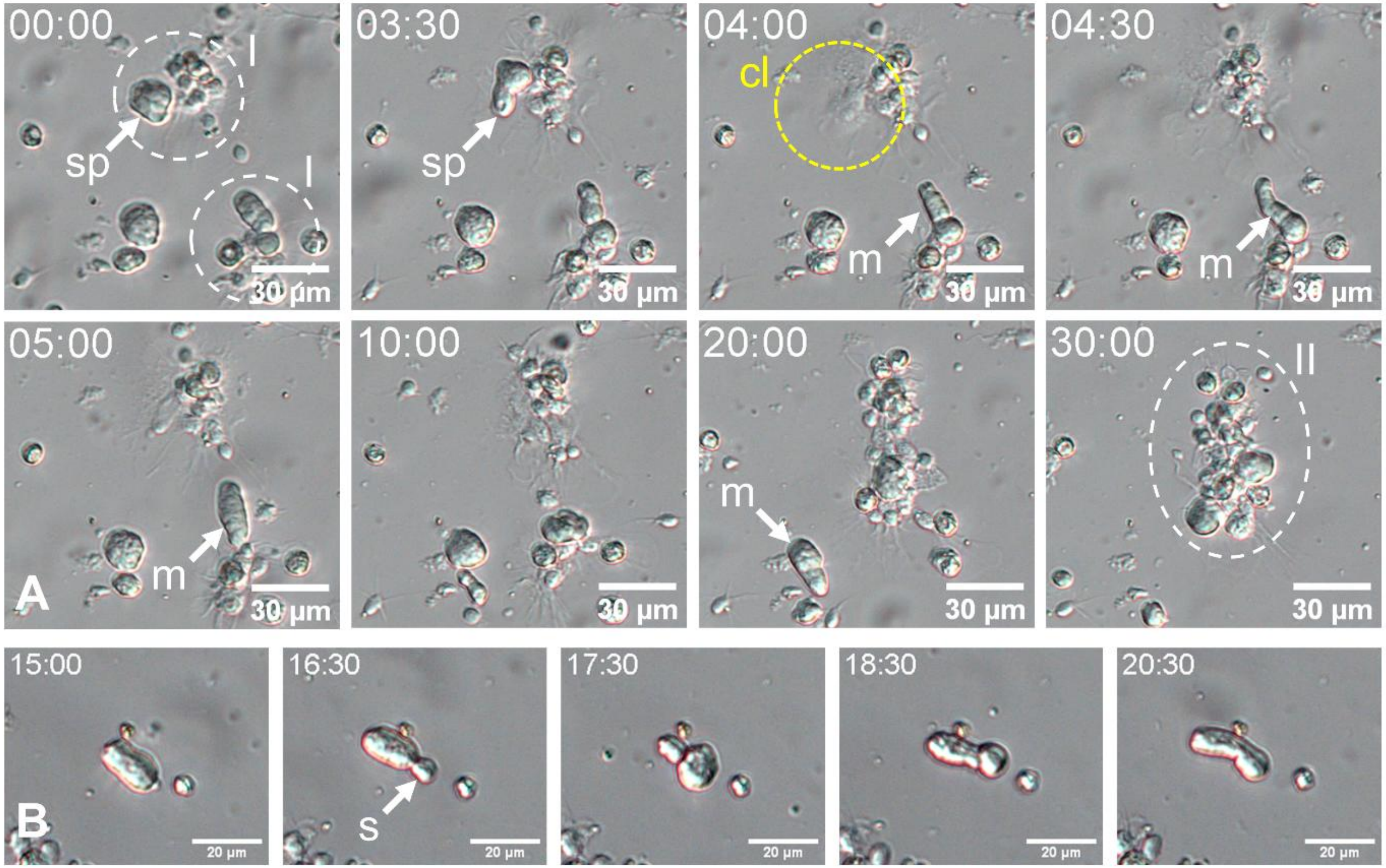

Sup. Fig. 3. Spherule cell-specific behaviours and mobility. **A.** Time-lapse imaging (same as **Video S1**) showing a spherule cell achieving lysis in contact with an aggregate. Some spherule cells also display high mobility. **B.** Particular movement of a small spherulocyte displaying a bleb-driven-like mobility starting with a small spheroid at the apex of the cell, followed by an undulation throughout the cell membrane (time frames: min:sec). Legend: cl - cell lysis; I - stage I aggregate; II - stage II aggregate; m - mobility; s - spheroid; sp - spherule cell.
