# Supplementary material for: Carotenoid-based immune response in sea cucumbers relies on newly identified coelomocytes—the carotenocytes
Source: Front Immunol. 2025 Nov 6;16:1668167. doi: 10.3389/fimmu.2025.1668167 (PMC12631484; doi:10.3389/fimmu.2025.1668167)
Supplement: Supplementary Figure 4 — Automated cell analysis using the Particle Analysis tool in ImageJ software. [file Image4.pdf]

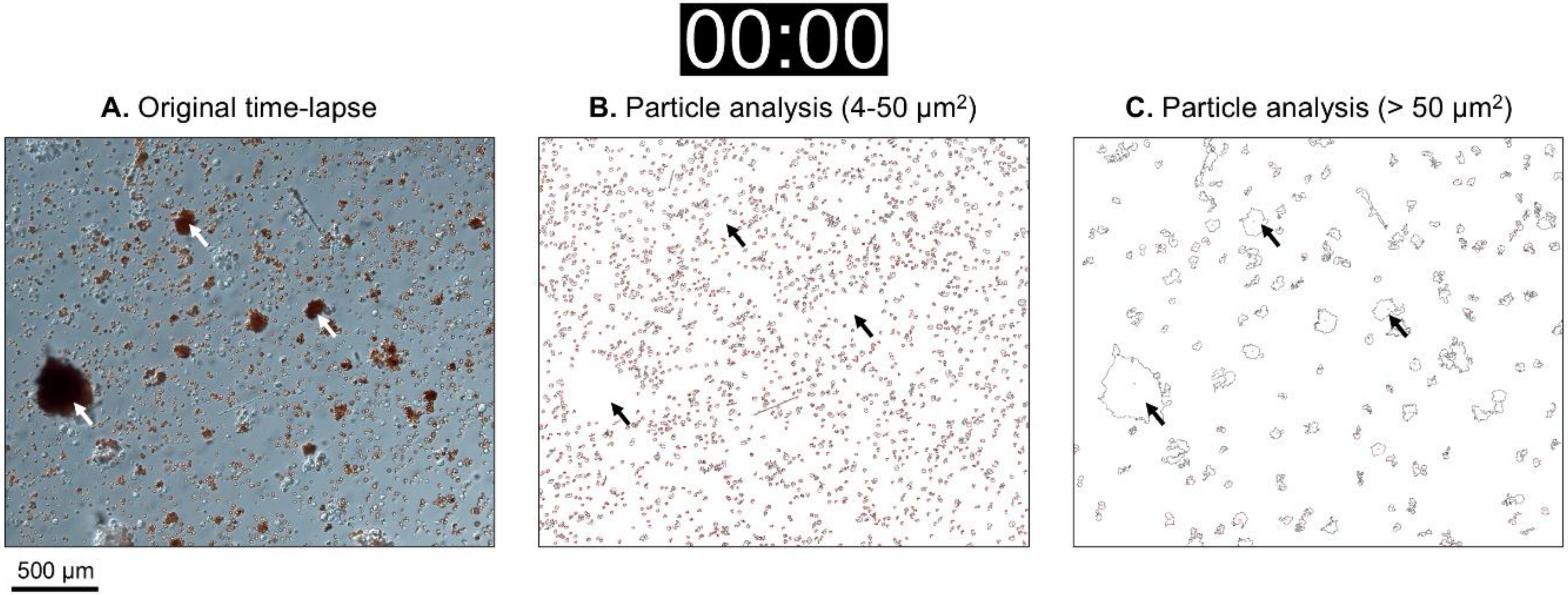

Sup. Fig. 4. Automated cell analysis using the Particle Analysis tool in ImageJ software. **A.** Original time-lapse converted to video in ImageJ. **B.** After applying a threshold filter, particle analysis was performed on an area between 4 and 50 μm^2 to target haemocyte-type cells. Each number corresponds to a cell. **C.** Particle analysis on an area greater than 50 μm^2, mainly targeting cell aggregates. Arrows indicate large aggregates in which no small cells (i.e., particles < 50 μm^2) are visible.
