# Supplementary material for: Carotenoid-based immune response in sea cucumbers relies on newly identified coelomocytes—the carotenocytes
Source: Front Immunol. 2025 Nov 6;16:1668167. doi: 10.3389/fimmu.2025.1668167 (PMC12631484; doi:10.3389/fimmu.2025.1668167)
Supplement: Supplementary Figure 5 — Transcriptome assembly and general annotation in Holothuria forskali. [file Image5.pdf]

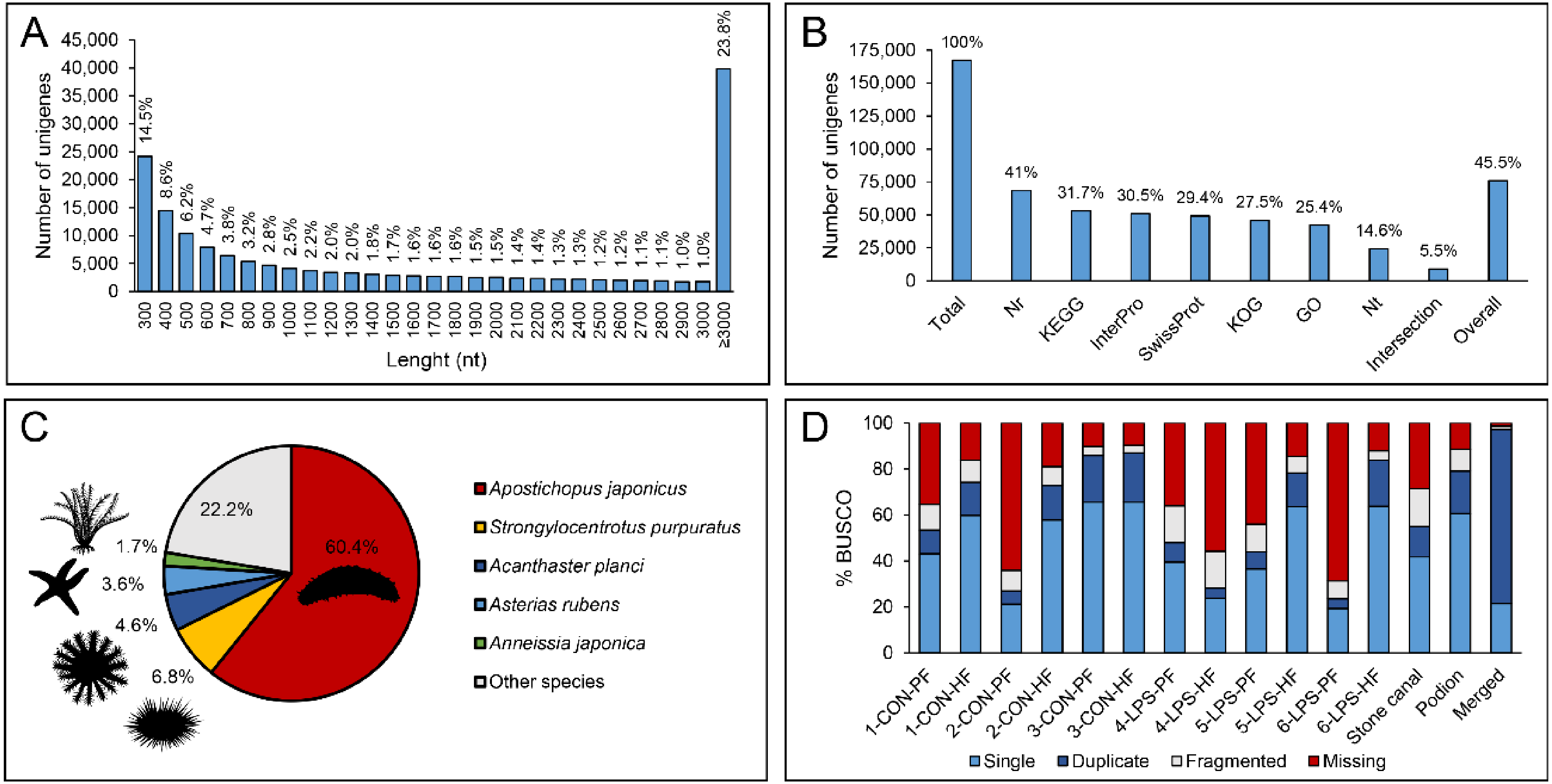

Sup. Fig. 5. Transcriptome assembly and general annotation in *Holothuria forskali*. A. Length distribution of unigenes (corresponding percentages are embedded in the graph). B. General annotations of unigenes against seven functional databases (match percentages are embedded in the graph). C. Species distribution for the Nr annotation (match percentages are embedded in the graph; species icons were modified from PhyloPic (www.phylopic.org). D. BUSCO assessment graph (CON – control individual; HF – hydrovascular fluid; LPS – LPS-injected individual; PF – perivisceral fluid).
