# Supplementary material for: Carotenoid-based immune response in sea cucumbers relies on newly identified coelomocytes—the carotenocytes
Source: Front Immunol. 2025 Nov 6;16:1668167. doi: 10.3389/fimmu.2025.1668167 (PMC12631484; doi:10.3389/fimmu.2025.1668167)
Supplement: Supplementary Figure 7 — Linear relationship between astaxanthin and canthaxanthin concentrations. [file Image7.pdf]

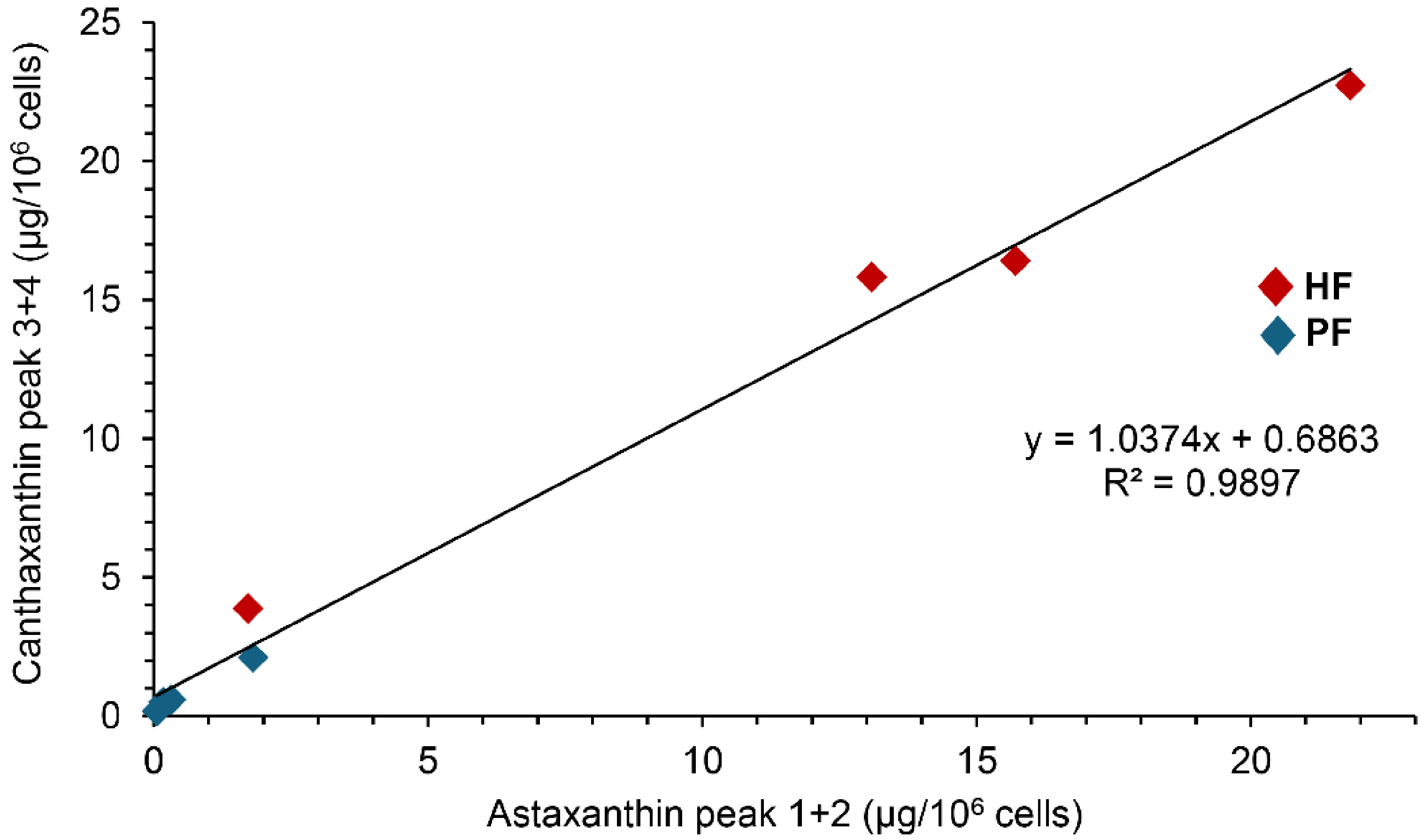

Sup. Fig. 7. Linear relationship between astaxanthin and canthaxanthin concentrations as determined by high-performance liquid chromatography (HPLC), in perivisceral fluid and in hydrovascular fluid of *Holothuria forskali*. Peaks refer to the spectra shown in Figure 6F. Peak 1+2: addition of the concentrations of the areas under the peaks (AUP) corresponding to astaxanthin. Peak 3+4: addition of the concentration of the areas under the peak (AUP) corresponding to canthaxanthin. The concentrations of these two types of carotenoids demonstrate a high coefficient of determination (R^2). The equation of the linear regression and the determination coefficient (R^2) are indicated on the graph. Legend: HF – hydrovascular fluid; PF – perivisceral fluid**.**
