# Supplementary material for: Carotenoid-based immune response in sea cucumbers relies on newly identified coelomocytes—the carotenocytes
Source: Front Immunol. 2025 Nov 6;16:1668167. doi: 10.3389/fimmu.2025.1668167 (PMC12631484; doi:10.3389/fimmu.2025.1668167)
Supplement: Supplementary Figure 8 — Autofluorescence (AF) spectra of coelomocytes revealed by spectral flow cytometry. [file Image8.pdf]

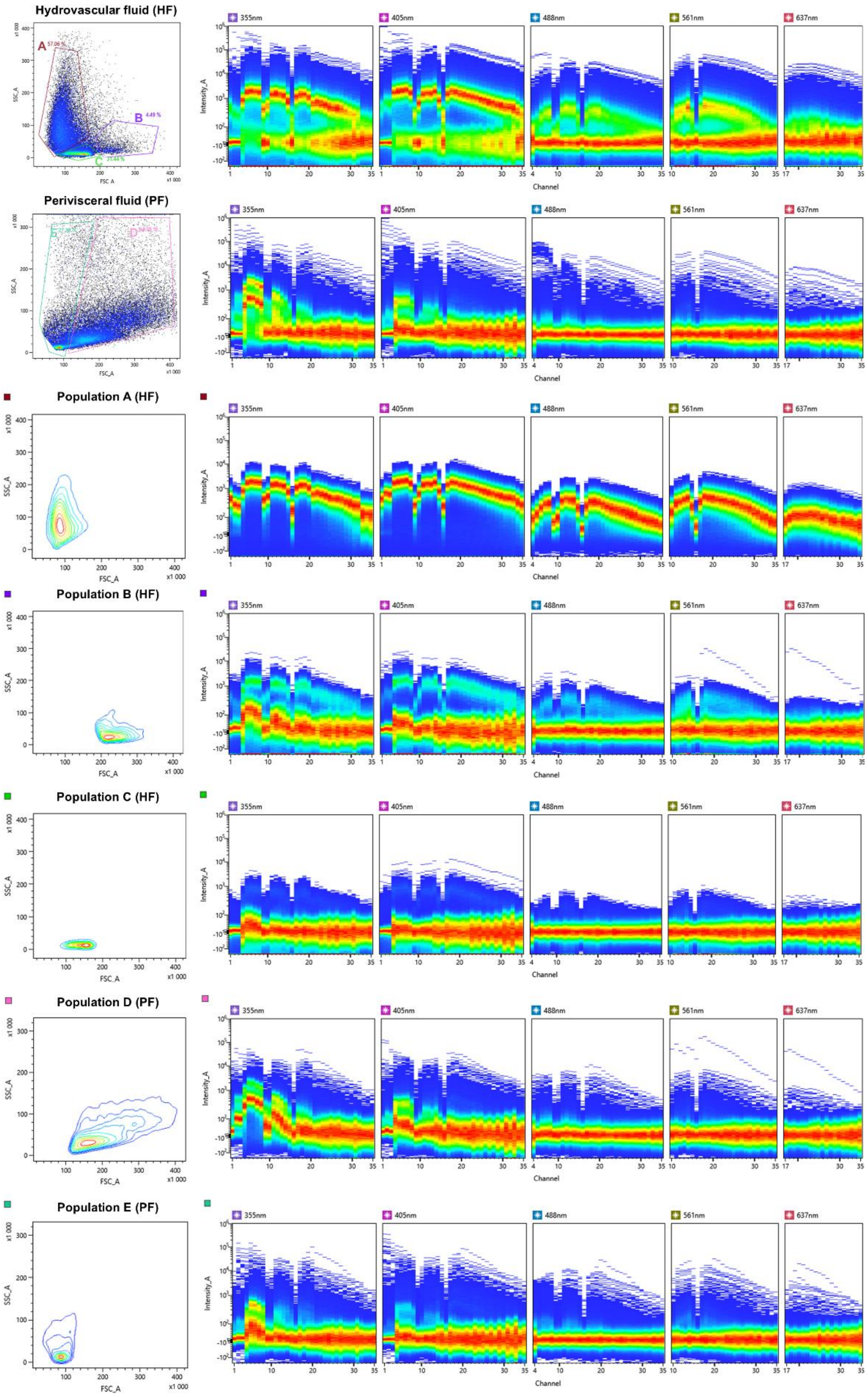

Sup. Fig. 8. Autofluorescence spectra of coelomocytes revealed by spectral flow cytometry. The first two spectra correspond to the two body fluids, respectively, perivisceral fluid (PF) and hydrovascular fluid (HF). The order corresponds to the different defined populations in Fig. 7. It is noticed that HF show a higher proportion of autofluorescent cells, and that among these population A, corresponding to Haemocyte-like cells (HELs), is the most autofluorescent population in all the lasers used.
