# Supplementary material for: Carotenoid-based immune response in sea cucumbers relies on newly identified coelomocytes—the carotenocytes
Source: Front Immunol. 2025 Nov 6;16:1668167. doi: 10.3389/fimmu.2025.1668167 (PMC12631484; doi:10.3389/fimmu.2025.1668167)
Supplement: Supplementary Figure 9 — String protein networks based on genes overexpressed in the perivisceral fluid (PF). [file Image9.pdf]

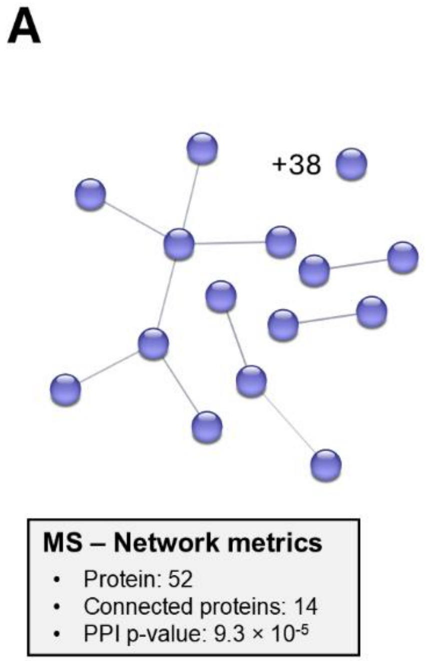

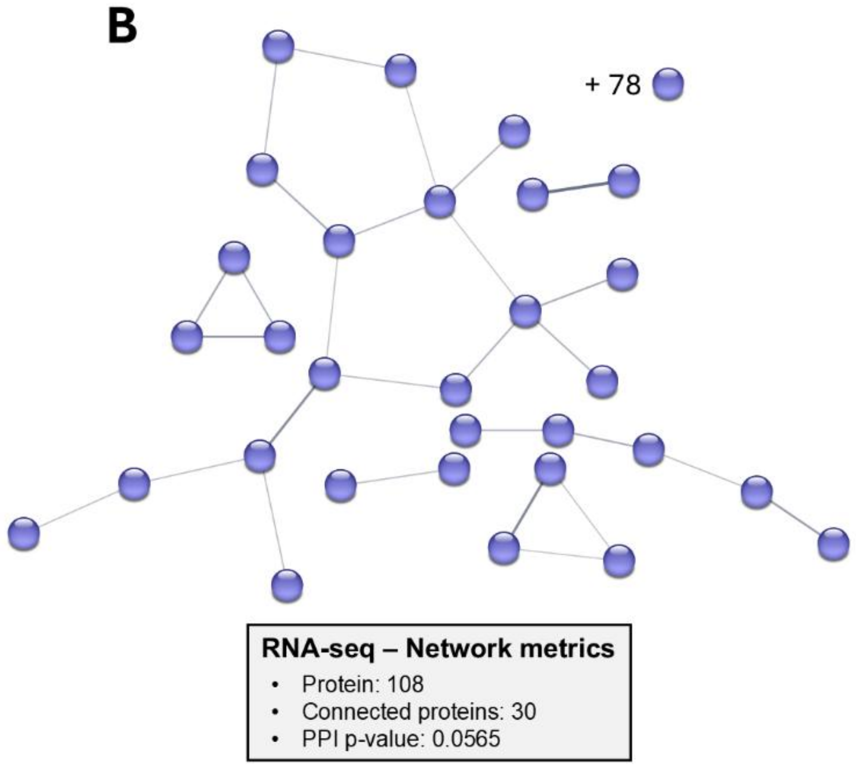

Sup. Fig. 9. String protein networks based on genes overexpressed in the perivisceral fluid (PF), in comparison to the hydrovascular fluid (HF: networks in Fig. 11). **A.** and **B.** are proteins identified based on mass-spectrometry (MS) analysis (fold change (HFvsPF) > 5) and RNA-sequencing (RNA-seq) analysis (fold change (HFvsPF) > 2), respectively. It can be noticed that protein networks are smaller than those of HF, with lower protein-protein interaction p-values.
