# Supplementary material for: Carotenoid-based immune response in sea cucumbers relies on newly identified coelomocytes—the carotenocytes
Source: Front Immunol. 2025 Nov 6;16:1668167. doi: 10.3389/fimmu.2025.1668167 (PMC12631484; doi:10.3389/fimmu.2025.1668167)
Supplement: Supplementary Figure 11 — Validation of DCFH-DA labelling for reactive oxygen species (ROS) detection. [file Image11.pdf]

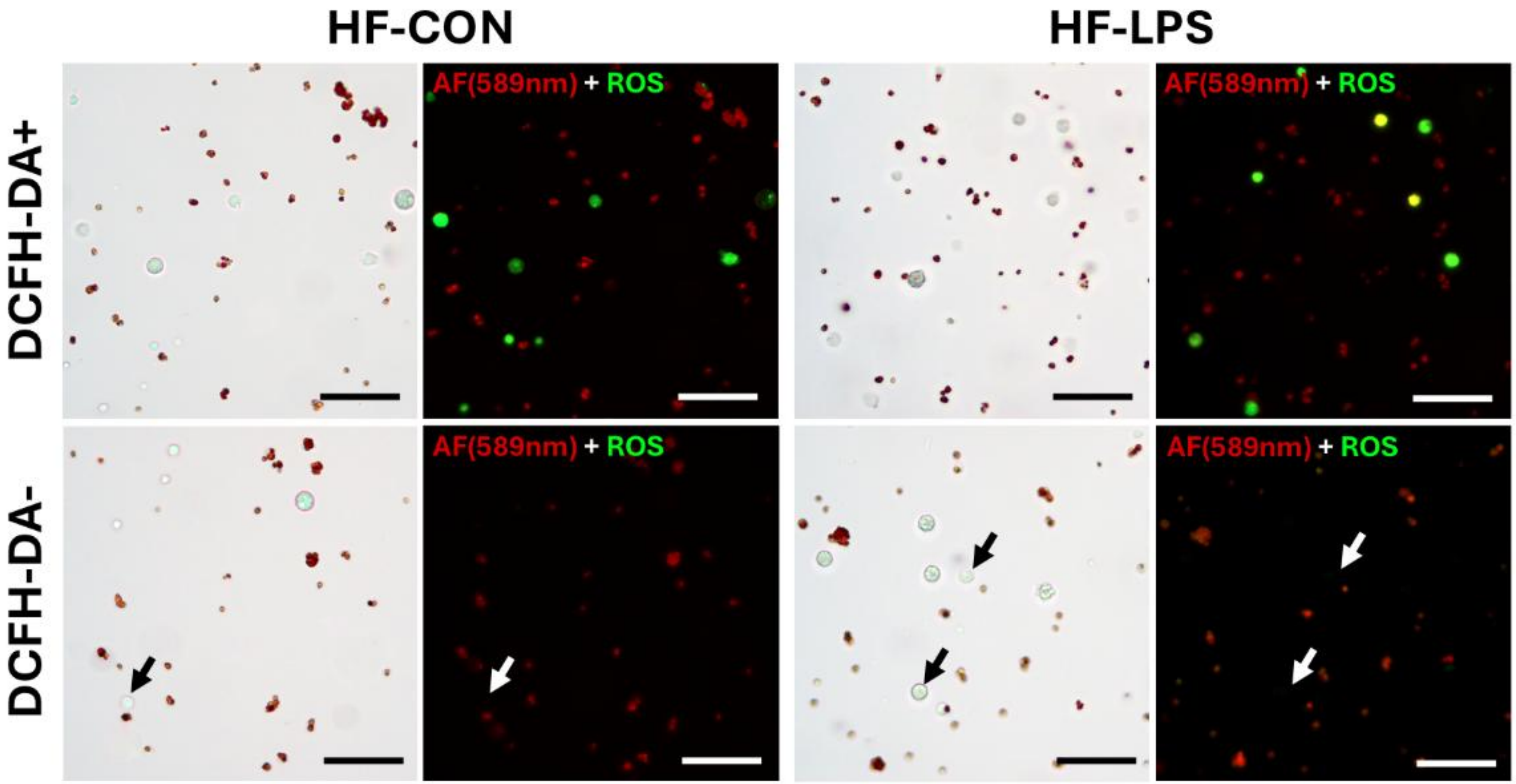


**Sup. Fig. 11.** Validation of DCFH-DA (2′−7′-dichlorodihydrofluorescein diacetate) labelling for reactive oxygen species (ROS) detection. Comparison of the same sample with and without the immunological stress (50µg/ml of lipopolysaccharides), and with the labelling of DCFH-DA (above) and without DCFH-DA (negative control – below). Positive cells (in green) are only observed in the presence of DCFH-DA. Autofluorescence (AF) at 589 nm was used to highlight the presence of haemocyte-like cells (HELS – in red). Note that these are not positive to ROS (the arrow indicates where cells are when they are not visible in fluorescent microscopy). The scale bar represents 50 µm (CON – control; HF – hydrovascular fluid; LPS – lipopolysaccharide-exposure).
