# Supplementary material for: Carotenoid-based immune response in sea cucumbers relies on newly identified coelomocytes—the carotenocytes
Source: Front Immunol. 2025 Nov 6;16:1668167. doi: 10.3389/fimmu.2025.1668167 (PMC12631484; doi:10.3389/fimmu.2025.1668167)
Supplement: Supplementary Figure 12 — Flow cytometry gating strategy of reactive oxygen species (ROS) production analysis. [file Image12.pdf]

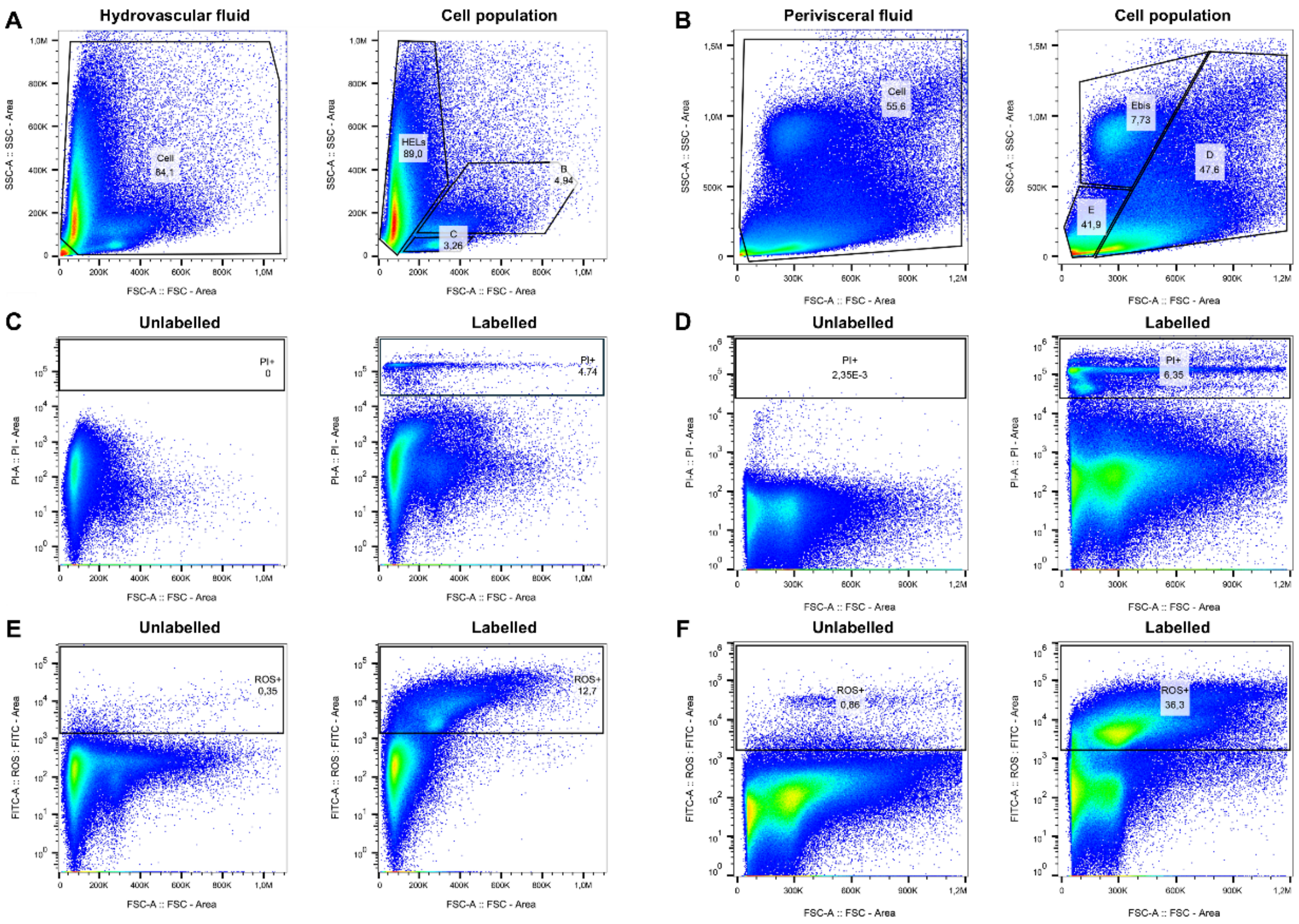

Sup. Fig. 12. Flow cytometry gating strategy of ROS production analysis. A. and B. Flow cytometry profile of hydrovascular (HF) and perivisceral fluids (PF), respectively. C. and D. Cell mortality analysis using propidium iodide labelling in the HF and the PF, respectively. Negative control (i.e., unlabelled sample) is shown for each fluid. E. and F. ROS production analysis using propidium iodide labelling in the HF and the PF, respectively. Negative control (i.e., unlabelled sample) is shown for each fluid.
