# Supplementary material for: Carotenoid-based immune response in sea cucumbers relies on newly identified coelomocytes—the carotenocytes
Source: Front Immunol. 2025 Nov 6;16:1668167. doi: 10.3389/fimmu.2025.1668167 (PMC12631484; doi:10.3389/fimmu.2025.1668167)
Supplement: Supplementary Figure 13 — Estimation of cell mortality using propidium iodide (PI) labelling and flow cytometry. [file Image13.pdf]

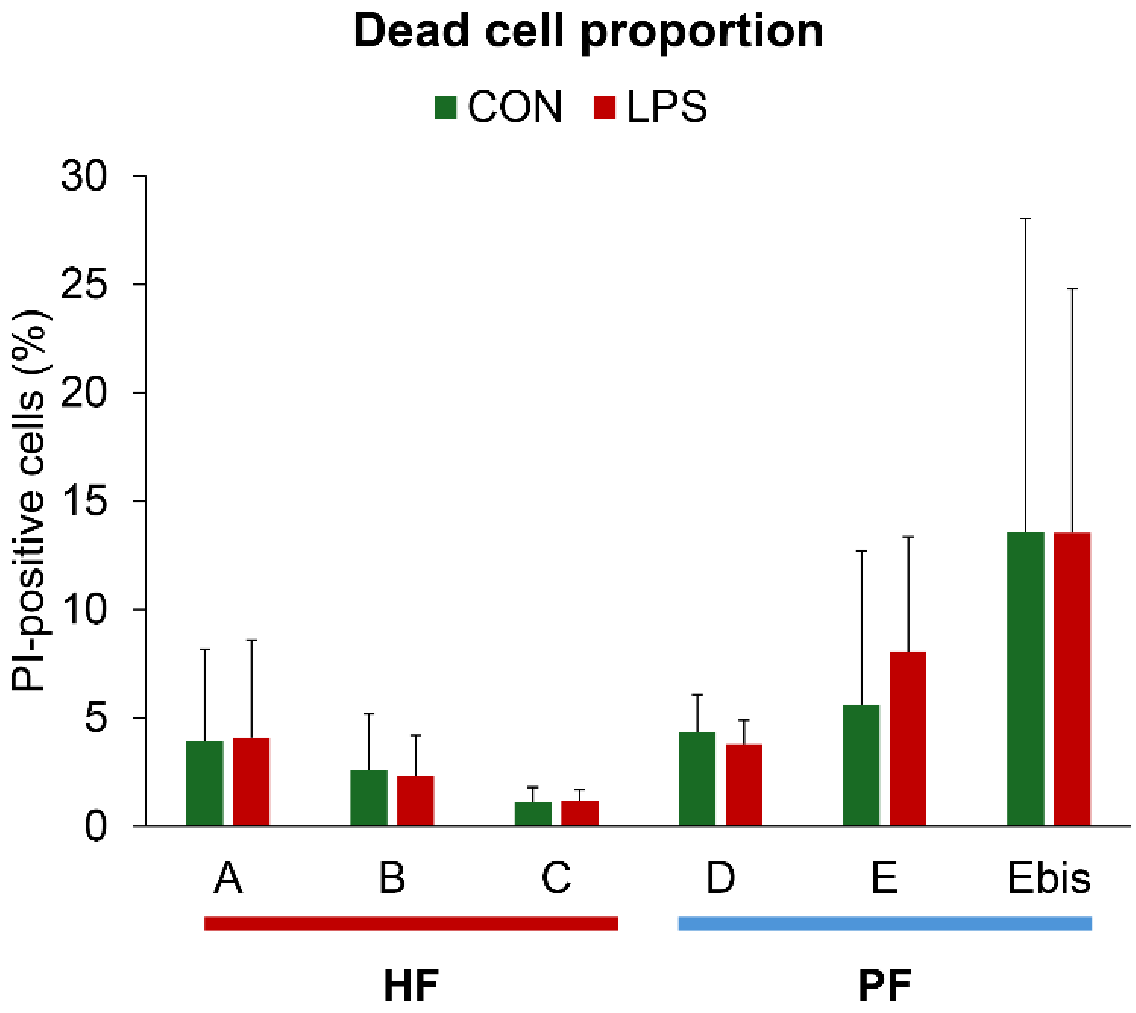

Sup. Fig. 13. Estimation of cell mortality using propidium iodide labelling and flow cytometry. Cell mortality proportion was low, with no clear difference between populations or between coelomocytes exposed or not to lipopolysaccharides. Error bars represent SD. Different populations correspond to those defined in Fig. 13. Legend: CON – control; HF – hydrovascular fluid; LPS – lipopolysaccharide-exposure; PF – perivisceral fluid.
